# GWAS for autoimmune Addison’s disease identifies multiple risk loci and highlights *AIRE* in disease susceptibility

**DOI:** 10.1038/s41467-021-21015-8

**Published:** 2021-02-11

**Authors:** Daniel Eriksson, Ellen Christine Røyrvik, Maribel Aranda-Guillén, Amund Holte Berger, Nils Landegren, Haydee Artaza, Åsa Hallgren, Marianne Aardal Grytaas, Sara Ström, Eirik Bratland, Ileana Ruxandra Botusan, Bergithe Eikeland Oftedal, Lars Breivik, Marc Vaudel, Øyvind Helgeland, Alberto Falorni, Anders Palmstrøm Jørgensen, Anna-Lena Hulting, Johan Svartberg, Olov Ekwall, Kristian Johan Fougner, Jeanette Wahlberg, Bjørn Gunnar Nedrebø, Per Dahlqvist, The Norwegian Addison Registry Study Group, Helge Ræder, Nevena Jovanovic, Sigfrid Christine Reisegg, Geir Hølleland, Siri Carlsen, Tore Julsrud Berg, Jan Bertil Eggesbø, Thomas Svendsen, Kari Lima, Ingrid Nermoen, Rolf Whitfield, Stina Sollid, Dagfinn Aarskog, Elin Korsgaard, Solveig Sæta, Trine Finnes, Susanna Fonneland Valland, Christian Fossum, Eli Brevik, Ragnar Bekkhus Moe, Margrethe Svendsen, Aleksandra Debowska, Petya Milova, Synnøve Holte, Aneta Eva Tomkowicz, Dag Eirik Sørmo, Anders Svare, Marthe Landsverk Rensvik, Randi Revheim, Thor Haug, Ivar Blix, Lars Petter Jensen, The Swedish Addison Registry Study Group, Anna-Karin Åkerman, Anna-Lena Hulting, Bengt Lindberg, Berit Kriström, Erik Waldenström, Gudmundur Johannsson, Jakob Skov, Jeanette Wahlberg, Karel Duchen, Magnus Isaksson, Maria Elfving, Maria Halldin Stenlid, Ola Nilsson, Olle Kämpe, Olov Ekwall, Per Dahlqvist, Ragnhildur Bergthorsdottir, Ricard Nergårdh, Sigridur Björnsdottir, Sophie Bensing, Tommy Olsson, Per Morten Knappskog, Anette Susanne Bøe Wolff, Sophie Bensing, Stefan Johansson, Olle Kämpe, Eystein Sverre Husebye

**Affiliations:** 1 Centre for Molecular Medicine, Department of Medicine (Solna), Karolinska Institutet, Stockholm, Sweden; 2 Department of Clinical Genetics, Uppsala University Hospital, Uppsala, Sweden; 3 Department of Immunology, Genetics and Pathology, Uppsala University, Uppsala, Sweden; 4 Department of Clinical Science, University of Bergen, Bergen, Norway; 5 K.G. Jebsen Center for Autoimmune Diseases, University of Bergen, Bergen, Norway; 6 Department of Medical Genetics, Haukeland University Hospital, Bergen, Norway; 7 Science for Life Laboratory, Department of Medical Sciences, Uppsala University, Uppsala, Sweden; 8 Department of Medicine, Haukeland University Hospital, Bergen, Norway; 9 Department of Endocrinology, Metabolism and Diabetes, Karolinska University Hospital, Stockholm, Sweden; 10 Department of Molecular Medicine and Surgery, Karolinska Institutet, Stockholm, Sweden; 11 Center for Diabetes Research, Department of Clinical Science, University of Bergen, Bergen, Norway; 12 Department of Genetics and Bioinformatics, Domain of Health Data and Digitalisation, Institute of Public Health, Oslo, Norway; 13 Department of Medicine, University of Perugia, Perugia, Italy; 14 Section of Specialized Endocrinology, Department of Endocrinology, Oslo University Hospital, Oslo, Norway; 15 Tromsø Endocrine Research Group, Department of Clinical Medicine, UiT The Arctic University of Norway, Tromsø, Norway; 16 Division of Internal Medicine, University Hospital of North Norway, Tromsø, Norway; 17 Department of Pediatrics, Institute of Clinical Sciences, Sahlgrenska Academy, University of Gothenburg, Gothenburg, Sweden; 18 Department of Rheumatology and Inflammation Research, Institute of Medicine, Sahlgrenska Academy, University of Gothenburg, Gothenburg, Sweden; 19 Department of Endocrinology, St. Olavs Hospital, Trondheim, Norway; 20 Department of Endocrinology, Linköping University, Linköping, Sweden; 21 Department of Health, Medicine and Caring Sciences, Linköping University, Linköping, Sweden; 22 Department of Internal Medicine, Haugesund Hospital, Haugesund, Norway; 23 Department of Public Health and Clinical Medicine, Umeå University, Umeå, Sweden; 24 Department of Pediatrics, Haukeland University Hospital, Bergen, Norway; 25 Department of Medicine, Haraldsplass Diaconess Hospital, Bergen, Norway; 26 Førde Central Hospital, Førde, Norway; 27 Department of Endocrinology, Stavanger University Hospital, Stavanger, Norway; 28 Institute of Clinical Medicine, University of Oslo, Oslo, Norway; 29 Lovisenberg Diakonale Hospital, Oslo, Norway; 30 Diakonhjemmet Hospital, Oslo, Norway; 31 Department of Pediatric Medicine, Oslo University Hospital, Oslo, Norway; 32 Department of Endocrinology, Akershus University Hospital, Lørenskog, Norway; 33 Vestre Viken Hospital Trust, Bærum, Norway; 34 Vestre Viken Hospital Trust, Drammen, Norway; 35 Vestre Viken Hospital Trust, Kongsberg, Norway; 36 Vestre Viken Hospital Trust, Ringerike, Norway; 37 Section of Endocrinology, Innlandet Hospital Trust, Hamar, Norway; 38 Department of Medicine, Innlandet Hospital, Gjøvik, Norway; 39 Akershus University Hospital, Division Kongsvinger Hospital, Kongsvinger, Norway; 40 Østfold Hospital Trust, Grålum, Norway; 41 Department of Medicine, Vestfold Hospital Trust, Tønsberg, Norway; 42 Telemark Hospital Trust, Notodden, Norway; 43 Department of Medicine, Sørlandet Hospital, Arendal, Norway; 44 Department of Medicine, Sørlandet Hospital, Kristiansand, Norway; 45 Nord-Trøndelag Hospital Trust, Levanger, Norway; 46 Nord-Trøndelag Hospital Trust, Namsos, Norway; 47 Ålesund Hospital, Ålesund, Norway; 48 Volda Hospital, Volda, Norway; 49 Molde Hospital, Molde, Norway; 50 Kristiansund Hospital, Kristiansund, Norway; 51 Nordland Hospital Trust, Bodø, Norway; 52 Department of Medicine, Örebro University Hospital, Örebro, Sweden; 53 Department of Clinical Sciences, Lund University Clinical Research Centre, Skåne University Hospital, Malmö, Sweden; 54 Institute of Clinical Science/Pediatrics, Umeå University, Umeå, Sweden; 55 Department of Endocrinology, Skåne University Hospital, Lund, Sweden; 56 Department of Internal Medicine and Clinical Nutrition, Institute of Medicine, Sahlgrenska Academy, University of Gothenburg, Gothenburg, Sweden; 57 Department of Endocrinology, Sahlgrenska University Hospital, Gothenburg, Sweden; 58 Department of Medicine, Karlstad Central Hospital, Karlstad, Sweden; 59 Division of Children’s and Women’s Health, Department of Biomedical and Clinical Sciences, Linköping University, Linköping, Sweden; 60 Department of Medical Sciences, Uppsala University, Uppsala, Sweden; 61 Department of Clinical Sciences, Pediatrics, Skåne University Hospital Lund, Lund University, Lund, Sweden; 62 Department of Women’s and Children’s Health, Karolinska Institutet, Stockholm, Sweden; 63 School of Medical Sciences, Örebro University, Örebro, Sweden; 64 Department of Pediatric Endocrinology, Akademiska University Hospital, Uppsala, Sweden

**Keywords:** Disease genetics, Adrenal gland diseases

## Abstract

Autoimmune Addison’s disease (AAD) is characterized by the autoimmune destruction of the adrenal cortex. Low prevalence and complex inheritance have long hindered successful genetic studies. We here report the first genome-wide association study on AAD, which identifies nine independent risk loci (*P* < 5 × 10^−8^). In addition to loci implicated in lymphocyte function and development shared with other autoimmune diseases such as *HLA*, *BACH2*, *PTPN22* and *CTLA4*, we associate two protein-coding alterations in *Autoimmune Regulator* (*AIRE*) with AAD. The strongest, p.R471C (rs74203920, OR = 3.4 (2.7–4.3), *P* = 9.0 × 10^−25^) introduces an additional cysteine residue in the zinc-finger motif of the second PHD domain of the AIRE protein. This unbiased elucidation of the genetic contribution to development of AAD points to the importance of central immunological tolerance, and explains 35–41% of heritability (*h*^2^).

## Abstract (web summary)

Autoimmune Addison’s disease is a rare complex disease, which has not yet been characterized by non-biased genetic studies. Here, the authors perform the first GWAS for the disease, identifying nine loci including two coding variants in the gene Autoimmune Regulator (*AIRE*).

## Introduction

Autoimmune Addison’s disease (AAD) is the most common cause of primary adrenal failure in the Western world^1^. It is a rare disease, affecting from five individuals per million in Japan, to more than 200 per million in the Nordic countries^2,3^. The disease requires lifelong steroid hormone replacement therapy and is fatal if untreated. Autoimmune etiology is often apparent from the presence of other associated autoimmune diseases^4^, and is confirmed by the presence of autoantibodies against the adrenal enzyme 21-hydroxylase^5^.

Despite the high heritability of AAD, amounting to 97% in a Swedish twin study (95% CI 0.88–0.99)^6^, genetic factors contributing to disease development have remained poorly defined. Due to the limited size of previously studied cohorts, candidate gene studies have for long been the only feasible option, even though the approach is known to be biased and many results fail to replicate. Targeted investigations have associated AAD with variation in the human leukocyte antigen (HLA) region on chromosome 6p21^7–9^, and have also implicated other well-established autoimmune disease susceptibility genes such as *PTPN22*^10^, *CTLA4*^11–13^, and *CLEC16A*^14^. Targeted sequencing studies have further identified *BACH2*^15^ and *AIRE*^16^ as risk loci in AAD, but were limited to preselected gene panels and small sample sizes. A genome-wide association study (GWAS) in AAD has hitherto not been possible due to the insufficient size of available cohorts.

Here we utilize the two largest Addison’s disease biobanks in the world^17,18^, enabling us to uncover both known and novel associations. Most intriguingly, we link AAD to protein-coding risk variants in *AIRE*, a gene crucial for antigen presentation in the thymus and for central immunological tolerance.

## Results

### GWAS of autoimmune Addison’s disease

Our initial sample of 1457 unrelated cases was further filtered to ensure a homogenous cohort of patients with autoimmune adrenal failure. We selected only cases with serum autoantibodies against 21-hydroxylase, and removed cases with clinical manifestations indicating other disease etiologies. Individuals with autoimmune polyendocrine syndrome type-1 (APS-1)^4^ were identified and excluded using clinical criteria, cytokine autoantibodies, and *AIRE* gene sequencing. The main analysis encompassed 1223 cases with AAD and 4097 healthy controls (Supplementary Table 1 and Supplementary Note 1). Genotyping was performed on a single occasion on the Illumina Infinium Global Screening Array followed by phasing and imputation. We imputed genotypes from the Haplotype Reference Consortium, retaining more than 7 million variants with minor allele frequency (MAF) ≥ 1%. The case–control association was performed using logistic regression on allele dosages, with sex and the first five principal components as covariates (Supplementary Fig. 1). The genomic inflation factor (*λ*_*GC*_) was 1.05 and the linkage disequilibrium (LD) score regression coefficient 1.02 (SE 0.007) indicating a low inflation in test statistics, mostly due to polygenicity (Supplementary Fig. 2).

We assessed the proportion of heritability explained by additive effects of SNPs using the genome-wide complex trait analysis GCTA (genome-wide complex trait analysis) software^19^. To account for ascertainment bias, i.e., the enrichment of cases in our sample compared to the general population, we included disease prevalence in the calculation. Reports from Scandinavia have indicated a prevalence between 13 and 22 cases per 100,000 inhabitants, which corresponded to an SNP heritability rate for AAD between 34 and 40%^3,17,20^. In other words, 35–41% of the heritability estimated in twins (*h*^2^ ≈ 0.97) is explained by the SNPs covered in this GWAS^6^.

### Genome-wide significant risk loci

**Fig. 1:**
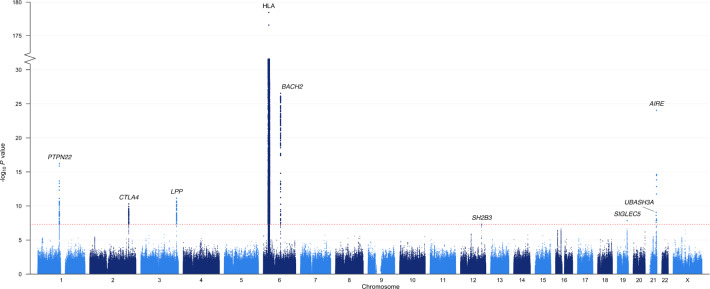
Manhattan plot for the genome-wide association study of autoimmune Addison’s disease with 1223 cases and 4097 controls. The –log_10_ *P* values from logistic regression on the *y*-axis are plotted against their physical chromosomal position on the *x*-axis for all SNPs across chromosomes 1–22 and X. Labels correspond to the prioritized or nearest genes. The dotted red bar marks the genome-wide significance level (*P* ≤ 5 × 10^−8^). The *y-*axis has been gapped to include the top SNP in the HLA region.

Our genome-wide analysis identified nine risk loci that exceeded the genome-wide significance (*P* ≤ 5 × 10^−8^; Fig. 1 and Table 1). Besides the HLA region, which stood out as the major risk locus (top SNP rs3998178, *P* < 10^−179^), we discovered AAD associations with variants in or adjacent to *PTPN22*, *CTLA4*, *LPP*, *BACH2*, *SH2B3*, *SIGLEC5*, *UBASH3A*, and *AIRE* (Supplementary Fig. 3). Of these associated loci, five had previously been implicated in AAD (*PTPN22*, *CTLA4*, HLA, *AIRE*, and *BACH2*), underlining the reliability of our results, whereas four loci were novel: *LPP*, *SH2B3*, *SIGLEC5*, and *UBASH3A*. To identify any further independent signals within the association peaks, we performed conditional regression analysis on the most significant SNP in each peak.

**Table 1:** Autoimmune Addison’s disease risk loci.

					Risk allele frequency		Association	
Locus^a^	Chr: Position^b^	SNP	Type^c^	Risk/alt allele^d^	Cases	Controls	OR (95% CI)	*P*
*PTPN22*	1: 114377568	rs2476601	p.R620W	A/G	0.17	0.11	1.74 (1.53–1.98)	6.3 × 10^−17^
*CTLA4*	2: 204707138	rs11571303	25 kb	G/A	0.69	0.61	1.39 (1.26–1.53)	5.0 × 10^−11^
*LPP*	3: 188112554	rs1464510	intronic	A/C	0.51	0.42	1.37 (1.25–1.5)	7.3 × 10^−12^
*HLA-DQB1*	6: 32623371	rs3998178	4 kb	T/C	0.51	0.19	5.98 (5.29–6.76)	3.5 × 10^−179^
*BACH2*	6: 90926612	rs10806425	5′ UTR	A/C	0.50	0.37	1.69 (1.53–1.85)	2.8 × 10^−27^
*SH2B3*	12: 111932800	rs7137828	43 kb	C/T	0.53	0.46	1.3 (1.18–1.42)	4.9 × 10^−8^
*SIGLEC5*	19: 52204248	rs8112143	70 kb	A/G	0.073	0.047	1.88 (1.51–2.34)	1.4 × 10^−8^
*UBASH3A*	21: 43836186	rs11203203	intronic	A/G	0.42	0.35	1.35 (1.22–1.48)	8.6 × 10^−10^
*AIRE*	21: 45714294	rs74203920	p.R471C	T/C	0.065	0.020	3.42 (2.71–4.32)	9.0 × 10^−25^
*AIRE*^e^	21: 45709153	rs2075876	intronic	G/A	0.95	0.90	2.17 (1.77–2.66)	7.8 × 10^−14^

Odds ratios and *P* values were estimated using logistic regression in 1223 cases diagnosed with autoimmune Addison’s disease and 4097 controls. Only results with *P* < 5 × 10^−8^ were reported to adjust for multiple testing.

^a^ The association peaks in chromosomes 1, 2, 12, and 19, span more than one gene, and the prioritized genes are reported. For HLA, the gene closest to the top SNP is reported.

^b^ Base-pair coordinates according to human reference genome GRCh37.

^c^ Functional characterization of SNPs overlapping prioritized genes, or distance from the SNP to the prioritized gene.

^d^ The risk allele indicates the effect allele for the OR, the second position gives the alternative allele.

^e^ Results for *AIRE* rs2075876 are from an analysis conditioning on the genotypes of the top SNP in *AIRE*, rs74203920.

We also carried out a fine-mapping analysis of each association peak, bar that centered on HLA, for which the results are summarized in Supplementary Data 1. Most loci had only one credible configuration, and those that had several had highly overlapping ones (2:3 SNPs common to all configurations). When limited to a single causal SNP, most loci had many SNPs in the credible set (range 7–43). Only SNPs with a log_10_(Bayes Factor) > 2, indicating strong support for causality versus the null hypothesis, are reported in Supplementary Data 1.

### Association with the *Autoimmune Regulator* gene

**Fig. 2:**
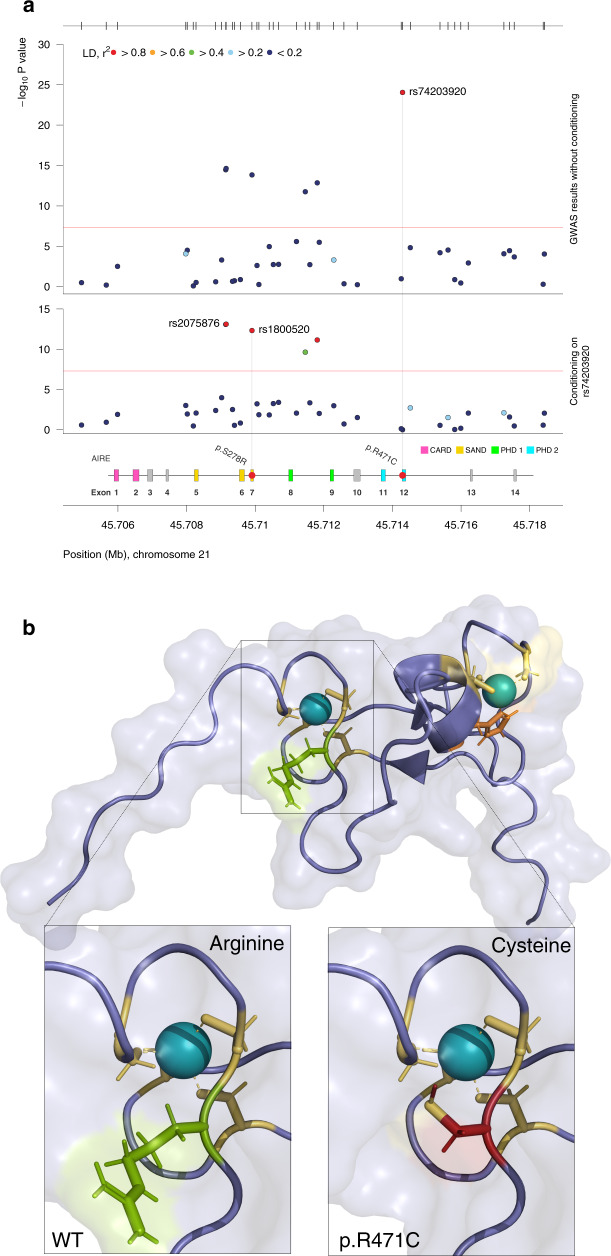
Two coding variants in the Autoimmune Regulator gene (*AIRE*) are independently associated with autoimmune Addison’s disease. **a** GWAS results without (upper panel) and with (lower panel) conditioning, on the top SNP rs74203920. The secondary association peak, including rs1800520, remains equally significant after conditioning reflecting its independent association. The –log_10_ *P* values from logistic regression of 1223 cases and 4097 controls are plotted against their physical chromosomal position. The red bars represent the genome-wide significance level (5 × 10^−8^). **b** The location and consequences of the coding change p.R471C in the PHD2 domain of AIRE. The additional charge from the cysteine residue (red) is in close proximity to the zinc ion (teal). Arginine is marked in green, histidine in orange, and wildtype (WT) cysteines in yellow.

Given that mutations in *AIRE* cause the monogenic disease APS-1 (OMIM #240300), of which AAD is a major component, this association peak was investigated in particular detail. Conditioning on the top *AIRE* SNP rs74203920, we found a second independent signal in *AIRE* (rs2075876, *P*_cond._ < 7.8 × 10^−14^), which was not in LD with the covariate rs74203920 (*r*^2^ < 0.01) (Fig. 2). Of these two independent associations, the top SNP rs74203920 was a novel association, whereas rs2075876 has been investigated previously^16^.

As we had carefully excluded cases of APS-1 using clinical data, serology, and genetic information, the strong association with the lead SNP in *AIRE* was a striking finding (rs74203920, OR = 3.4 (2.7–4.3), *P* = 9.0 × 10^−25^). Comparing carriers with non-carriers of the p.R471C variant in our group of cases (*n* = 1223), we could not detect any differences in age of disease onset or presence of autoantibodies (Supplementary Table 2). Furthermore, to test whether the effect of rs74203920-T was associated with AAD alone or with autoimmune comorbidities, we divided cases into isolated AAD (*n* = 443), and those with AAD and type 1 diabetes or autoimmune thyroid disease, i.e., Autoimmune Polyendocrine Syndrome type-2 (*n* = 682) (Table 2)^4^. The risk allele rs74203920-T was equally enriched in both categories, and exceeded genome-wide significance regardless of autoimmune comorbidity.

**Table 2:** Risk allele frequencies (RAF) in isolated autoimmune Addison’s disease and autoimmune polyendocrine syndrome type 2 (APS-2).

Locus^a^	SNP	RAF controls	RAF isolated AAD	OR	*P*	RAF APS-2	OR (95% CI)	*P*
*PTPN22*	rs2476601	0.11	0.18	1.72 (1.42–2.08)	1.8 × 10^−8^	0.17	1.72 (1.46–2.02)	7.9 × 10^−11^
*CTLA4*	rs11571303	0.61	0.67	1.28 (1.11–1.48)	8.8 × 10^−4^	0.70	1.46 (1.29–1.66)	4.4 × 10^−9^
*LPP*	rs1464510	0.43	0.53	1.46 (1.27–1.67)	6.4 × 10^−8^	0.50	1.33 (1.19–1.5)	8.1 × 10^−7^
*HLA-DQB1*	rs3998178	0.19	0.51	5.72 (4.81–6.81)	6.5 × 10^−86^	0.52	6.31 (5.42–7.35)	2.6 × 10^−123^
*BACH2*	rs10806425	0.37	0.50	1.66 (1.44–1.91)	2.0 × 10^−12^	0.51	1.71 (1.52–1.93)	1.7 × 10^−18^
*SH2B3*	rs7137828	0.46	0.52	1.29 (1.12–1.49)	4.0 × 10^−4^	0.52	1.28 (1.13–1.44)	5.9 × 10^−5^
*SIGLEC5*	rs8112143	0.047	0.077	2.04 (1.49–2.8)	8.3 × 10^−6^	0.071	1.76 (1.33–2.31)	5.7 × 10^−5^
*UBASH3A*	rs11203203	0.35	0.40	1.24 (1.07–1.43)	3.6 × 10^−3^	0.44	1.4 (1.24–1.58)	5.9 × 10^−8^
*AIRE*	rs74203920	0.020	0.072	3.73 (2.74–5.09)	8.0 × 10^−17^	0.063	3.24 (2.43–4.31)	7.7 × 10^−16^
*AIRE*	rs2075876	0.90	0.94	1.85 (1.38–2.47)	3.5 × 10^−5^	0.96	2.67 (2.01–3.55)	9.7 × 10^−12^

Odds ratios and *P* values were estimated using logistic regression in isolated AAD (*n* = 443) and APS-2 (*n* = 682), respectively, compared to 4097 controls. Only loci with *P* < 5 x 10^−8^ in the overall analysis were tested.

^a^ The association peaks in chromosomes 1, 2, 12, and 19, span more than one gene, and the prioritized genes are reported. For HLA, the gene closest to the top SNP is reported.

Since APS-1 is a recessive disorder, we formally tested but could not find support for rs74203920 and/or rs2075876 causing AAD with recessive inheritance (Supplementary Table 3). Rather, the risk effects of both SNPs were best described by an additive model. Lastly, we tested for differential association with other loci in carriers versus non-carriers of rs74203920 and rs2075876, respectively, but found no differences between the groups (Supplementary Tables 4 and 5). Taken together, the associated *AIRE* variants exert their risk effect independently from each other and from other risk loci.

The risk allele rs74203920-T was uncommon in our Swedish and Norwegian controls (2.0%) and in the non-Finnish European population (1.4% GnomAD v2.1.1), but was strongly enriched among cases with AAD (MAF = 6.5%). The SNP is located in exon 12 and the risk allele encodes an arginine to cysteine substitution at amino acid residue 471 in the well-conserved zinc ion binding motif of the second PHD domain (PHD2) (Fig. 2a). The PHD2 domain of AIRE is stabilized by a zinc finger with two zinc ions, one of which is coordinated by amino acid residues C446, C449, C472, and C475^21^. Each zinc-binding residue is essential, as exemplified by the missense mutation p.C446G found in patients with APS-1, which destroys the structural fold of the PHD2 domain. By introducing an additional cysteine in the zinc-binding motif, it is possible that p.R471C alters the binding of the zinc ion and the structure of the PHD2 domain (Fig. 2b and Supplementary Fig. 4).

Within the second, independent association peak in *AIRE*, the top SNP rs2075876 was in LD with the coding SNP rs1800520 (*r*^2^ = 0.83). This variant, a serine to arginine substitution of amino acid residue 278 (p.S278R), is located in the SAND domain. Hence, two coding changes were independently associated with AAD. In a functional assay of AIRE function, neither .R471C nor p.S278R interfered with AIRE-dependent transcription (Supplementary Fig. 5 and Supplementary Note 2)^22^. With two independent associations with *AIRE*, AIRE-dependent antigen presentation, and central immune tolerance appears to play an important role in the development of AAD.

### Dissection of the HLA association

**Fig. 3:**
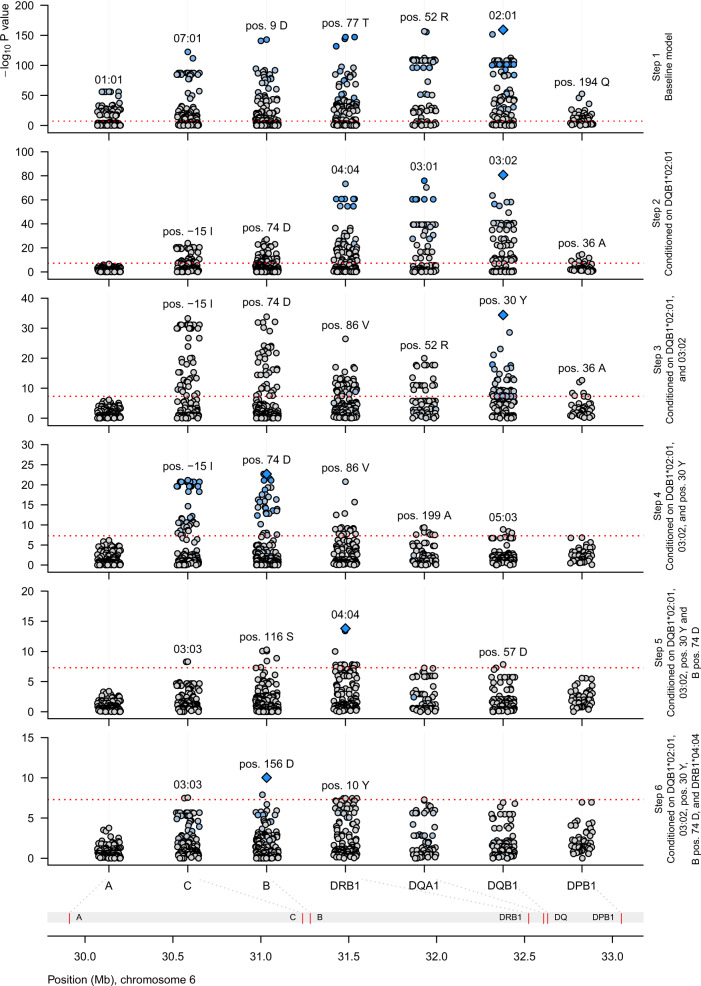
Stepwise regression of the HLA association identifies the major genetic determinants of autoimmune Addison’s disease. The figure displays the results from the first six steps of regression modeling of the HLA risk effects—alleles and amino acids. Starting with a baseline model comprising sex and five principal components as covariates, we tested every allele and amino acid in turn for association with AAD (Supplementary Note). Additive, recessive, dominant, overdominant, and general variable encodings were compared with likelihood ratio tests and/or Bayesian information criterion. The allele or amino acid residue with most compelling evidence for association was included in the model at every step, and reconsidered at all subsequent steps. Downstream regression models were conditioned on the effects selected from previous models. The *y-*axes show the –log_10_ *P* values from stepwise logistic regressions of 1223 cases and 4097 controls. The dashed horizontal lines indicate genome-wide significance (*P* < 5 × 10^−8^). Diamonds mark the most significant effect. Blue color indicates strong linkage disequilibrium (*r*^2^) with the most significant effect, gray color indicates no correlation.

The HLA region is by far the strongest risk locus in AAD, but due to long-range LD and genetic heterogeneity, the dissection of risk within the region is challenging. To define the key components of genetic risk attributable to HLA, we imputed classical HLA alleles and their corresponding amino acids across HLA class I and class II, and constructed a general logistic model for AAD risk (Fig. 3 and Supplementary Note 3). We report seven independent alleles and amino acids associated with AAD at the genome-wide significance level (*P* value <5 × 10^−8^; Table 3). Consistent with previous studies, we found that the risk was dominated by HLA-DQB1*02:01 (OR = 7.3, *P* = 1.9 × 10^−45^, under the full model including all reported effects), and HLA-DQB1*03:02 (OR = 2.3, *P* = 1.4 × 10^−21^), that tag the well-established risk haplotypes corresponding to serotypes DR3-DQ2 and DR4-DQ8, respectively^9,16,17^. We also found largely additive risks for DQB1 position 30 Tyr, B pos. 74 Asp, B pos. 156 Asp, and DQA1*01:04. The tyrosine residue in position 74 of HLA-B was the first representation of HLA class I to be included in the model, and had only a weak correlation with HLA class II (*r*^2^ = 0.22 with HLA-DQB1*02:01). Comparing cases (*n* = 232) and controls (*n* = 2719) that carry neither of the major two risk haplotypes, the two strongest of remaining risk haplotypes contained DQB1*03:01 and DQB1*04:02, both of which encode a tyrosine residue in DQB1 position 30.

**Table 3:** HLA alleles associated to autoimmune Addison’s disease.

		Frequency ^a^		Association		
HLA allele or amino acid	Parameter	Cases	Controls	OR (95% CI)^b^	*P* value^c^	HLA alleles in LD^d^
DQB1*02:01	Additive effect	0.39	0.12	7.29 (5.54–9.6)	1.9 × 10^−45^	DQA1*05:01, DRB1*03:01 (*r*^2^ > 0.95), B*08:01, C*07:01 (*r*^2^ > 0.5)
DQB1*03:02	Additive effect	0.28	0.14	2.25 (1.91–2.66)	1.4 × 10^−21^	DQA1*03:01 (*r*^2^ > 0.95), DRB1*04:04 (*r*^2^ = 0.43)
DQB1 pos. 30 Tyr	Additive effect	0.54	0.52	3.64 (2.9–4.59)	3.5 × 10^−28^	DQB1*06:02 (*r*^2^ = 0.16), DRB1*15:01 (*r*^2^ = 0.16)
B pos. 74 Asp	Additive effect	0.61	0.37	1.97 (1.71–2.26)	1.5 × 10^−21^	B*08:01 (*r*^2^ = 0.30), C*07:02 (*r*^2^ = 0.23)
DRB1*04:04	Allelic interaction with DQB1*02:01	0.16	0.044	6.66 (4.55–9.74)	1.4 × 10^−22^	DQA1*03:01 (*r*^2^ = 0.43), DQB1*03:02 (*r*^2^ = 0.43)
B pos. 156 Asp	Additive effect	0.46	0.25	1.69 (1.45–1.97)	2.9 × 10^−11^	B*08:01 (*r*^2^ > 0.5), C*07:01 (*r*^2^ = 0.41)
DQA1*01:04	Additive effect	0.012	0.021	3.85 (2.39–6.2)	3.0 × 10^−8^	DQB1*05:03 (*r*^2^ > 0.95), DRB1*14:01 (*r*^2^ > 0.95)

Odds ratios and *P* values were estimated using stepwise logistic regression in 1223 cases and 4097 controls. Only results with *P* < 5 × 10^−8^ were reported to adjust for multiple testing.

^a^ Allele frequency and amino acid frequency, respectively.

^b^ Estimated odds ratio from the full model.

^c^ *P* value from the full model. Alleles and amino acids are presented in order of inclusion.

^d^ HLA alleles with LD *r*^2^ > 0.5 are presented in categories of *r*^2^ > 0.5, >0.8, >0.9, and >0.95. Maximum 2 HLA alleles with *r*^2^ ≤ 0.5 are presented.

After defining the major risk effects, we next investigated the presence of interactions between HLA alleles and amino acids included in the model, and all other alleles and residues in the dataset. We identified a strong risk effect for DRB1*04:04 in the presence of DQB1*02:01 (OR = 6.7, *P* = 1.4 × 10^−22^). Beside this interaction, no other pairs of alleles and/or residues passed the significance threshold for inclusion in the full model.

**Fig. 4:**
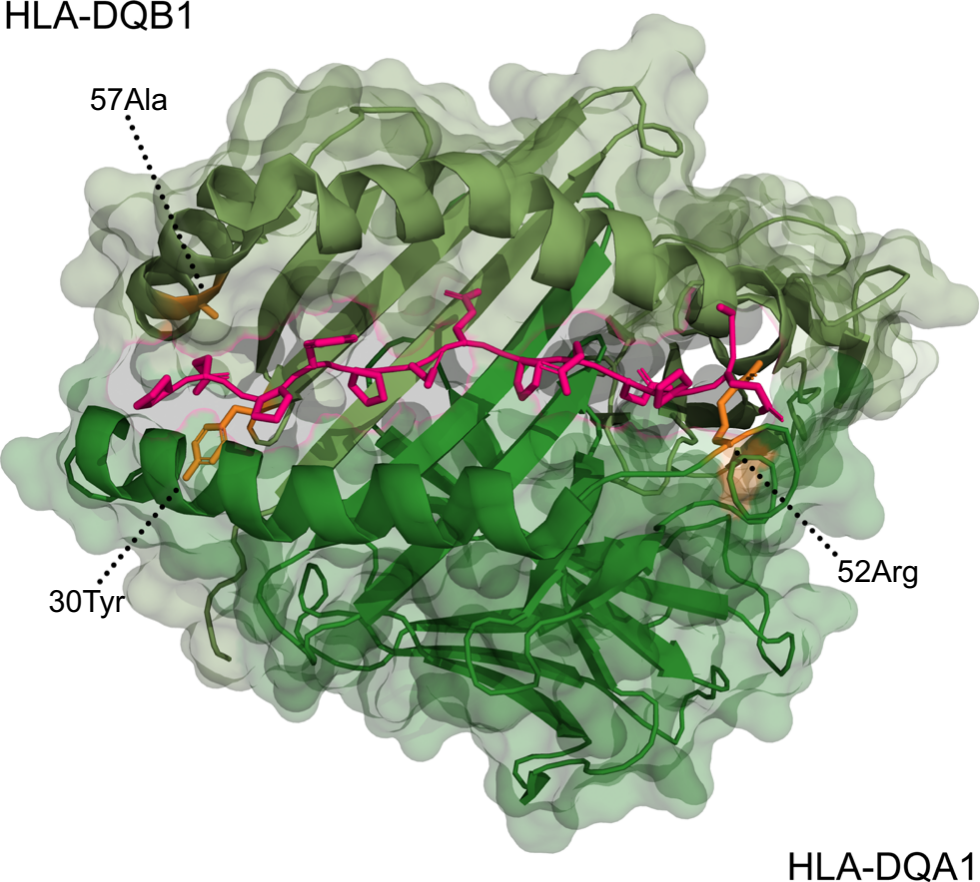
Associated amino acids in the HLA-DQ heterodimer. Two amino acids in HLA-DQB1 and one amino acid in HLA-DQA1 were found to be associated with autoimmune Addison’s disease. A tyrosine at the 30th position and an alanine at the 57th position of HLA-DQB1 (top) and an arginine at the 52nd position of HLA-DQA1 (bottom) have been marked in orange. To visualize the binding pocket, a peptide ligand (gliadin) from the original crystal structure has been marked in pink.

To avoid the risk of conditioning out critical amino acids in local LD with major risk alleles, we extracted and investigated the most significantly associated residues from each round of the stepwise regression (Supplementary Table 6). The emerging amino acids were the arginine in position 52 of DQA1 (OR = 7.8, *P* = 2 × 10^−157^) and the alanine in position 57 of DQB1 (OR = 4.3, *P* = 2.6 × 10^−152^). The latter is a distinctive feature of the allele DQB1*02:01. Even though the long-ranging LD in the HLA region makes it difficult to pinpoint causal variation, it is striking that also the third independent amino acid resides in the binding pocket of the HLA-DQ heterodimer (DQB1 pos. 30 tyrosine, OR = 3.6, *P* = 3.8 × 10^−35^) (Fig. 4).

### Established loci in AAD

**Fig. 5:**
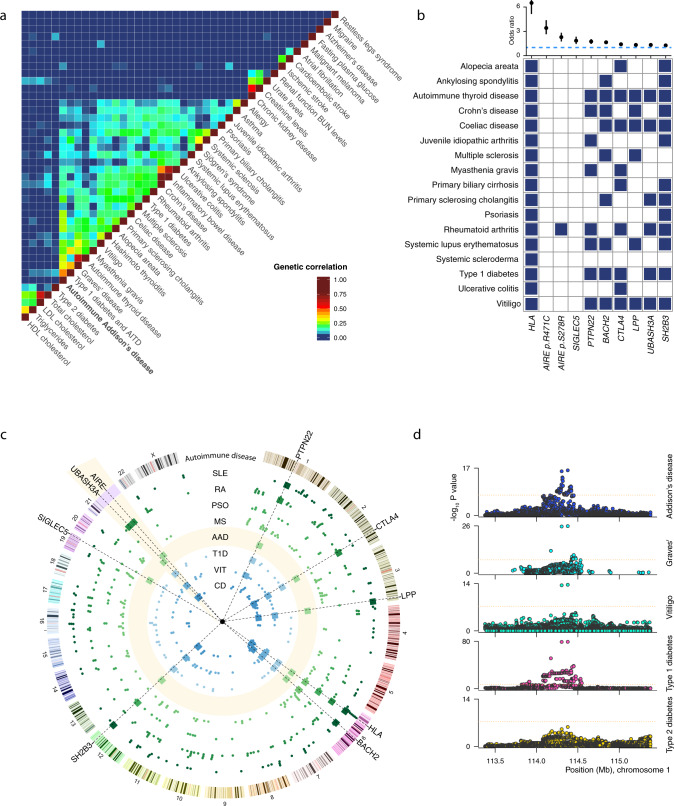
Shared genetic features. **a** Human diseases and traits studied in GWAS were clustered to reveal shared genetic risk factors. Diseases/traits are ordered by unsupervised hierarchical clustering, and color scale indicates genetic correlation. **b** Loci implicated in autoimmune Addison’s disease in order of decreasing effect size (odds ratio and 95% CI), 1223 cases, and 4097 controls. The horizontal, dashed line marks OR = 1. Blue squares indicate genome-wide significant associations for the diseases and loci/variants, respectively. **c** Circos plot representing the loci associated with AAD (boxes) and other autoimmune diseases (dots). The AAD track is highlighted in yellow, and the yellow wedge on chromosome 21 is magnified ×15. SLE-systemic lupus erythematosus, RA-rheumatoid arthritis, PSO-psoriasis, MS-multiple sclerosis, T1D-type 1 diabetes, VIT-vitiligo, CD-coeliac disease. **d** Side-by-side comparison of association statistics at the *PTPN22* locus across a selection of autoimmune diseases. AAD statistics were calculated using logistic regression for 1223 cases and 4097 controls.

The *PTPN22*, *CTLA4*, and *BACH2* loci are well-known drivers of autoimmune disease and we identified the variants and haplotype blocks that have previously been described in AAD and common autoimmune comorbidities (Fig. 5 and Supplementary Figs. 6 and 7). The fine-mapping analysis largely confirmed the missense variant in *PTPN22*, thought to be causal in a range of different autoimmune diseases. The credible set also included two eQTLs for BCL2L15, a weakly proapoptotic protein associated with autoimmune thyroid disease and type 1 diabetes^23,24^, in T helper cells. This differential regulation might constitute a secondary causal effect in addition to the canonical p.R620W variant in *PTPN22*.

For the chromosome 2 peak, no obviously immune relevant signals were identified in the credible set/configuration, though a couple of variants were located in enhancer-like sequences, and a GTEx eQTL gene for our prioritized *CTLA4*. In chromosome 6, almost all the variants in the credible set and configurations were *BACH2* eQTLs for naive T-cell populations in particular, several also have enhancer-like signatures and exhibit H3K27Ac-marks in some ENCODE cell lines, strengthening the postulation that AAD is driven by differential regulation of *BACH2* in cases versus controls.

### Novel loci in AAD

We also discovered four novel loci that achieved genome-wide significance for association with AAD. The ORs were lower than for loci detected previously, commensurate with the improved statistical power in this study. All the SNPs in the credible set and configuration for the chromosome 3 peak were located in introns of *LPP*, as is the lead SNP from the GWAS. With the exception of some weak transcription factor binding sites, there were no functional categories located at any of these SNPs.

The association peaks in chromosomes 12 and 19 encompassed several genes, but were ascribed to *SH2B3* and *SIGLEC5* after gene prioritization. The lead SNPs were barely genome-wide significant, and the most significant genotyped markers, as opposed to imputed markers, had *P* values of 7.8 × 10^−8^ and 4.3 × 10^−7^ for *SH2B3* and *SIGLEC5*, respectively.

The broad association at the *SH2B3* locus looked similar in studies of type 1 diabetes and vitiligo, giving credibility to the result, but making it challenging to appoint a single candidate gene. The credible set and configuration for chromosome 12 were eQTLs for a number of tissues and a handful of genes (mostly the same for each variant) and H3K27Ac-marks in several cell lines, but none that appeared particularly relevant to autoimmunity. However, one of the credible set SNPs is both located in an enhancer-like sequence and is itself a missense variant in *SH2B3*. This variant (p.W262R, MAF ≈ 0.5) is not predicted to be deleterious (by SIFT/PolyPhen). In the chromosome 19 credible set/configuration, whole blood eQTLs were present in one variant each for *SIGLEC14* (a MAPK/AKT-activating SIGLEC, as opposed to the DEPICT-prioritized*SIGLEC5*) and *SPACA6*.

In contrast to the above broad association peaks, the peak at the *UBASH3A* locus was well-defined and in a haplotype block containing no other genes but *UBASH3A*. eQTLs for *UBASH3A* exist for all credible configuration SNPs for various T-cell subpopulations, but all the variants in this locus’s credible configurations and sets are yet more significant eQTLs for all T cells in *TMPRSS3*.

Interestingly, most AAD GWAS peaks harbor a gene with a role in or near the immunological synapse, the connection between antigen-presenting cells and lymphocytes (*P* = 0.003) (Supplementary Fig. 8)^25^.

### Genetic correlations and loci shared with other autoimmune traits

Many autoimmune diseases co-occur in affected individuals and families, and share numerous genetic risk factors^26^. To explore the genetic architecture underlying AAD at large, we investigated the overlap of risk loci with diseases and traits previously investigated in other well-powered GWAS. By unsupervised clustering of overlapping risk loci, an extensive and complex sharing of risk loci among immunological diseases clearly emerged (Fig. 5a). Systemic autoimmune diseases, inflammatory bowel diseases, and organ-specific autoimmune diseases, respectively, formed distinct groups within this major cluster. The majority of patients with AAD develop at least one additional autoimmune disease, such as Hashimoto’s thyroiditis, type 1 diabetes, vitiligo, or Graves’ disease^18^. It was therefore interesting to note that AAD displayed a significant overlap of genetic risk loci with its most common comorbidities, reflecting the genetic risk factors shared between organ-specific autoimmune diseases.

We searched the National Human Genome Research Institute—European Bioinformatics Institute (EBI) GWAS catalog using our genome-wide significant AAD risk loci for associations with other autoimmune diseases (Fig. 5b, c). The overlapping loci included *UBASH3A* and *SH2B3* in type 1 diabetes and celiac disease, and *LPP* in autoimmune thyroid disease and vitiligo. In general, surveying autoimmune diseases with summary statistics available through the GWAS catalog and PhenoScanner, risk variants and haplotypes were strikingly often shared across diseases (Supplementary Figs. 6 and 7)^27,28^. In the case of *PTPN22*, which had data available for the largest number of diseases, confidence intervals of effect estimates were overlapping between diseases, indicating equivalent effects (Fig. 5d).

Notably, the strongest of our novel risk alleles, our lead SNP in *AIRE* (rs74203920), has not been linked to other autoimmune diseases in GWAS. Although our second independent signal in *AIRE* has previously been associated with AAD, and also with rheumatoid arthritis in East Asia^16,29,30^, the allele that increases the susceptibility to AAD (rs2075876-G), appears associated with decreased risk of rheumatoid arthritis. Thus, risk alleles at most loci appear to be general drivers of autoimmunity, whereas the risk alleles in *AIRE* are more specifically associated with AAD.

## Discussion

**Fig. 6:**
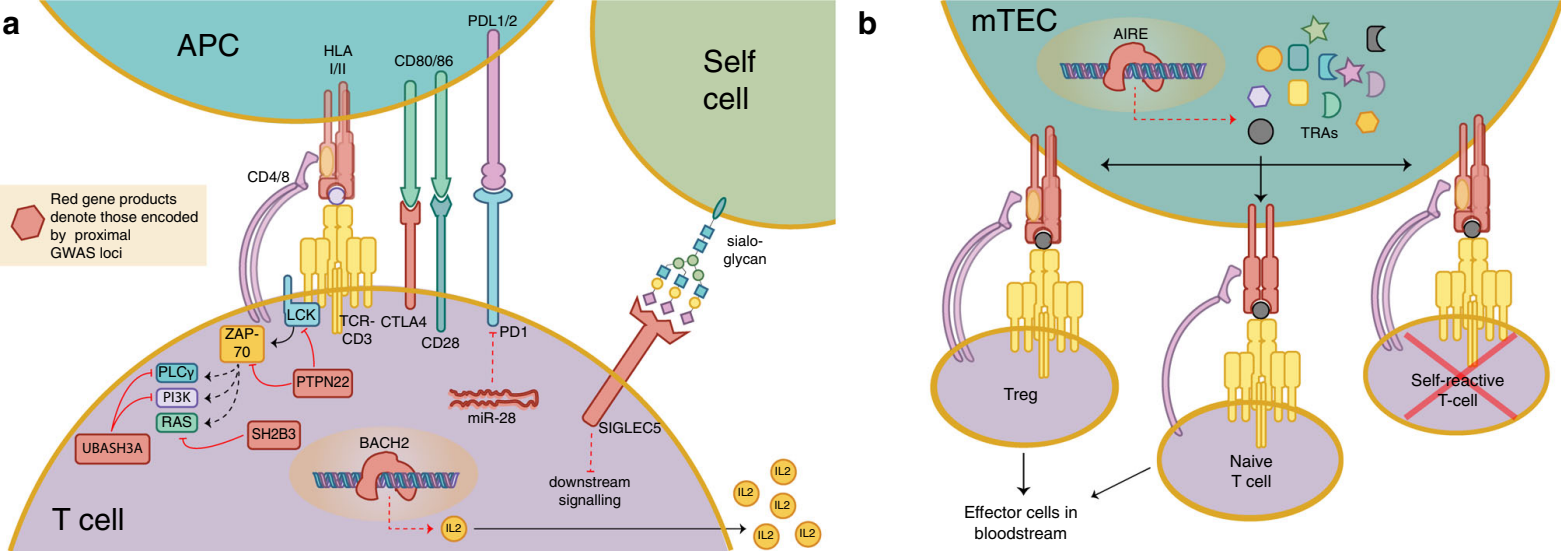
T-cell regulation and AAD GWAS associated regions. **a** Graphic representation of selected aspects of T-cell regulation, with gene products implicated by GWAS association proximity in red (antigen-presenting cell, APC). **b** AIRE activity in medullary thymic epithelial cell (mTEC), promoting expression of tissue-restricted antigens (TRAs) for the education of T cells.

This GWAS of AAD identified nine genome-wide significant associations and explained 35–41% of the additive genetic heritability (*h*^2^). The results point to the complex network of antigen presentation and immunomodulation that underlie autoimmune disease development (Fig. 6). In particular, two independent associations in *AIRE* highlight the importance of central immune tolerance in AAD pathogenesis. *AIRE* is essential for thymic expression of otherwise tissue-specific proteins, and hence important for negative selection of autoreactive thymocytes and prevention of organ-specific autoimmune disease. As strongly deleterious mutations in *AIRE* cause APS-1, it is particularly interesting that we associate two LD-independent protein-coding variants in *AIRE* with sporadic AAD.

Given the allele frequency of 1.5–2.0% in the general population, the effect of the variant with the strongest association, p.R471C, must be subtle compared to mutations known to cause APS-1. p.R471C has previously been reported in single cases of multi-organ autoimmunity, including patients with AAD and type 1 diabetes^31^, or AAD and autoimmune thyroiditis^32^. None of the reported cases had interferon autoantibodies, which would be expected in patients with APS-1^33^. Thus, current evidence does not support p.R471C as a cause of APS-1, but instead points to an increased risk of AAD at the population level.

p.R471 is located near two highly conserved cysteine residues that coordinate a critical zinc ion in the second PHD zinc finger (p.C472 and p.C475). PHD fingers maintain the structural integrity, read methylation states of histones, and regulate gene expression through formation of complexes with chromatin regulators and transcription factors^34^. Our transfection assay did not show an effect of rs74203920 or rs1800520 on AIRE-dependent transcription, for which there may be several reasons. While AIRE is predominantly expressed and exerts its main functions in the thymus, HEK293 cells have despite their renal origin shown large overlap in AIRE-regulated genes with primary thymic cells from mice^35–37^. It is thus likely that p.R471C has an effect on AAD susceptibility that differs from classical deleterious mutations that cause APS-1. Furthermore, though it cannot be excluded that variation in other nearby genes is involved, for instance the inducible T-cell costimulator (*ICOSLG*), the credible set from fine-mapping of the locus includes only p.R471C.

The HLA region is implicated in autoimmune disease and confers by far the largest risk for AAD compared to other known risk loci. We dissected the HLA-mediated risk of AAD in detail, confirming known associations and suggesting additional susceptibility alleles^8,9,15,38^. Our results demonstrate that risk is dominated by alleles in HLA class II, in particular the two major risk haplotypes, and an interaction between the two indicates a shared mechanism. We also identified strong effects mediated by HLA class I. For instance, aspartic acid in residues 74 and 156 of HLA-B are both represented in HLA-B*08:01, one of the few alleles that have been successfully investigated in functional studies on antigen presentation of 21-hydroxylase in AAD patients^39^. Notably, we could not detect any interactions between HLA class I and class II, nor any interactions between pairs of alleles conferring risk and protection. With a larger sample size, however, additional effects could potentially be uncovered and incorporated in a similar model. These results provide a foundation for further work aimed at understanding the exact mechanisms underlying HLA-mediated risk and for functional studies of implicated HLA alleles in antigen presentation.

The two independent associations with *AIRE* point to alterations in central immunological tolerance as an underlying mechanism in AAD development. The importance of a correct expression of *AIRE* for maintaining immunological tolerance is also exemplified by Down Syndrome in which the extra copy of *AIRE*, located on chromosome 21, has been coupled to altered expression of *AIRE* in the thymus, impaired central tolerance and an overrepresentation of autoimmune diseases^40,41^. Many of the other risk loci identified in this study harbor genes involved in antigen presentation and recognition, and hence in thymocyte maturation. Beside HLA class II that presents antigens to developing T cells, the turnover of the T-cell antigen receptor (TCR) complex is regulated by UBASH3A^42^, and the immune checkpoint CTLA4 modulates the co-stimulation required for T-cell activation^43^. Alternatively, or in addition to *UBASH3A*, the putative causal variants identified by fine-mapping suggest a role in AAD for *TMPRSS3* in T cells; the risk alleles are linked to higher expression levels, an effect also seen in type 1 diabetes^44^. The tryptophan substitution in position 620 of *PTPN22* (p.R620W) disrupts the formation of complexes between PTPN22 and CSK, and the inhibitory effect on TCR signaling is attenuated^45^. BACH2 has been shown to stimulate (CD8+) T-cell differentiation by controlling access of transcription factors to their enhancers and to promote differentiation of regulatory T cells^46^. SH2B3, suggested to be the causal entity behind the common autoimmune ATXN2/SH2B3 association^47^, like UBASH3A above, is an inhibitor of signaling cascades in lymphocytes^47^. While the most highly associated variants lie nearer the 5′ end of the gene, *LPP* harbors a microRNA, miR-28, that appears to be involved in posttranscriptional regulation of PD1^48,49^ which has an important role in self-tolerance, restraining autoreactive T cells and promoting Tregs^50^.

The association peak on chromosome 19 provides three different potentially causal units: *SIGLEC5* (prioritized by DEPICT), *SIGLEC14* (whole blood eQTL in the credible configuration), and *SPACA6* (whole blood eQTL in the credible set). The latter harbors miR-125a, a miRNA that appears to be involved in posttranscriptional regulation of *KLF13*^51^, which in turn regulates *CCL5*, a chemokine that affects the activation, migration, and proliferation of T cells^52^. *SIGLEC14*, an activating receptor highly homologous to the nearby *SIGLEC5*, is hard to rule out, though the interpretation is further complicated by the fact that a common polymorphism leads to a *SIGLEC14/5* fusion gene and effectively a *SIGLEC14*-null phenotype^53^. *SIGLEC5*, as it recognizes self-cell surface sialoglycans and mediates inhibitory signaling in T cells^54^, is the most likely candidate from a cell biology standpoint. Figure 6 summarizes and highlights these T-cell-related effects of the most likely functional gene products associated with our GWAS hits.

We identified robust association signals despite relatively modest sample sizes compared to many other autoimmune disorders, which indicate a rather homogenous disease etiology with relatively low polygenicity compared to other diseases, underpinning the high heritability estimates from epidemiological studies. We believe that a strength of this study was the opportunity to recruit the majority of AAD patients in Norway and Sweden through national registries with geographically matched controls, using stringent exclusion criteria and only including those with 21-hydroxylase autoantibodies.

To conclude, our results highlight the importance of central immune tolerance in the development of AAD. Dysregulation of antigen presentation in the setting of negative selection in the thymus may be one of the factors that makes AAD exceptional among organ-specific autoimmune diseases, and the pathways identified should be explored in the development of preventive treatment strategies.

## Methods

### Subjects

Cases were recruited from the Swedish and Norwegian Addison Registries and fulfilled clinical diagnostic criteria for primary adrenal insufficiency, i.e., low serum cortisol with a compensatory increase in plasma adrenocorticotropic hormone^1,17,18^. In case of doubt, a corticotropin stimulation test was performed. Autoimmune etiology was confirmed by the presence of highly specific autoantibodies targeting the adrenal-specific enzyme 21-hydroxylase, the major autoantigen in AAD^5^. Cases were screened for APS-1 using clinical criteria, autoantibodies against interferon-α, interferon-ω, or interleukin 22, and/or *AIRE* gene sequencing^55,56^. Healthy controls were recruited from blood donor centers across Sweden and Norway to match the geographical coverage of registry cases.

All study subjects gave their informed consent. The study was performed in accordance with the Declaration of Helsinki and approved by the local ethics committees in Stockholm, Sweden (dnr 2008/296-31/2), and Western Norway (biobank 2013-1504, project 2017-624).

### DNA extraction

Blood samples were kept at −80 °C until processed at the HUNT Laboratory (Levanger, Norway). DNA was isolated using the MasterPure™ DNA purification kit version II B1 (Epicenter^®^, Madison WI), and normalized to 50 ng/µl. In total, 200 ng of each sample was pipetted by robot to 96-well plates (Abgene Storage Plates, ThermoFisher). Swedish/Norwegian and case/control samples were distributed in equal proportions in the plates. Technical replicates were included to facilitate quality control and genotype concordance between plates.

### Genotyping, imputation, and quality control

Genome-wide genotyping of 692,367 markers was performed using the Illumina Infinum Global Screening Array 1.0 by the Human Genomics Facility at Erasmus MC (Rotterdam, the Netherlands). Markers and samples were filtered iteratively using PLINK version 1.9 (Supplementary Note 1)^57^. In short, markers were first excluded based on call rate <95% or deviation from GenomeStudio genotype clusters. Second, samples were excluded on the basis of sample call rates <98%. Third, in-depth marker quality control excluded markers with call rate <98%, discordant calls in technical replicates, or deviation from Hardy–Weinberg equilibrium (HWE, *P* < 10^**−**6^). For X chromosome markers, HWE tests were performed in females only. Finally, samples with accumulated heterozygosity >0.34 were excluded.

Bi-allelic SNPs that passed the above QC thresholds and that were present in the Haplotype Reference Consortium panel^58^ were used for phasing and imputation. Genotypes were phased in-house to take advantage of available pedigree information (SHAPEIT version 2.r837)^59^, and non-typed variants imputed using the Sanger Imputation Service (PBWT) and the Haplotype Reference Consortium release 1.1^58^. Markers with imputation quality score >0.5 and MAF > 0.01 were included in the GWAS.

Global ancestry was inferred using the LASER/TRACE software^60^ with the Human Genome Diversity Project reference panel^61^. Samples estimated to be non-European were excluded. Genetic relatedness was evaluated using high-quality markers pruned for LD in PLINK. For each pair of related samples ($\hat \pi$ > 0.1), cases and males were preferentially selected, otherwise the sample with the highest call rate was retained. In total, data from 5320 samples and 7.1 million markers were kept for association testing.

### GWAS

Main axes of genetic variation, as a proxy for population substructure, were assessed using principal component analysis of high-quality markers with MAF > 0.05, pruned for LD (*r*^2^ < 0.2), and excluding the extended HLA region. Association statistics were calculated using logistic regression of disease status on genotype dosages. Sex and the top five principal components were included as covariates to account for potential sex differences and confounding population stratification.

### Conditional analysis

Loci passing the genome-wide significance threshold for association were subject to conditional analysis to identify any independent associations. This was done by stepwise inclusion of imputed genotype dosages for the index variants as covariates in logistic regression.

### Gene prioritization at associated loci

Enrichment of association signals in physiological systems, tissues, and cell types, as well as prioritization of genes for each association, was performed using the computational tool DEPICT from GWAS summary statistics (Data-driven Expression Prioritized Integration for Complex Traits, https://broadinstitute.org/mpg/depict/)^25^. We used default settings with 500 permutations to adjust for gene length differences, and 50 repetitions to compute false discovery rates. The false discovery rate was set to 5%. Associated loci were plotted with LocusZoom^62^.

### Fine-mapping

Fine-mapping was carried out using FINEMAP^63^, in two runs. In total, 1 Mb windows around the lead SNPs for each genome-wide association peak (except that of HLA) were assessed, allowing maximum one or three causal SNPs per window per run, and otherwise default settings. It must, however, be noted that for small studies, such as ours is in a modern GWAS context, the benefits of such stochastic fine-mapping are likely to be small^64^.

### HLA imputation and dissection

Classical HLA alleles were imputed from directly genotyped and phased SNPs using HIBAG kernel version 1.4^65^ and SNP2HLA version 1.0.3^66^. The classifiers and reference panels for European samples provided with each software were used to impute two field (four digit) alleles in *HLA-A*, -*B*, -*C*, -*DQA1*, -*DQB1*, and -*DRB1*. To improve the precision of *DRB1*-allele calls, we built a model for HLA imputation using a reference panel of 699 healthy Norwegian controls. The DRB1 alleles were called by combining predictions from the default and in-house model, both weighted by the size of their training data. Genotypes were kept and treated as fixed if their posterior probability was at least 0.5 and more than twice as likely as the second most probable call. For 370 of the case samples, laboratory typing of *HLA-A*, *-B* (most single field), -*DQB1*, and -*DRB1* (most two field) was available. Concordance for the three former was 92–98% for both HIBAG and SNP2HLA, while it was 90% for DRB1.

We aimed at identifying the main drivers of risk mediated by classical HLA alleles and amino acids across HLA class I and class II, with a method adapted from Moutsianas et al.^67^. For every HLA allele in turn, we constructed several logistic regression models and used Bayesian information criterion and likelihood ratio test to help decide whether the effect was best described as additive, recessive, dominant, overdominant, or general. See the Supplementary Note 3 for details of the selection procedure. In short, the allele or amino acid residue effect from the most significant model was considered for inclusion in the full model. Five PCs and sex were included as covariates in all models. PCs were calculated from SNPs genome-wide, not including the HLA region, i.e., the same covariates as in the GWAS analysis. We only considered the inclusion of alleles/amino acids that met genome-wide significance *P* < 5 × 10^−8^, both at time of inclusion, and in the final model. Backward elimination was performed by leaving previous variables out of the current model, one by one, and by subsequently testing the goodness of fit.

### Loci shared with common diseases and traits

Genetic overlap was investigated using a method adapted from Farh et al.^68^. In short, GWAS catalog data were obtained from the EMBL-EBI website (https://ebi.ac.uk/gwas/), current as of December 2019^69^. Diseases/traits with at least six reported associations (*P* ≤ 1 × 10^−6^) were included. Because many diseases/traits have been subject to more than one independent GWAS, they had multiple associated SNPs at the same locus. For these loci, defined as a window of 500 kb, only the most significant index SNP was considered. In total, 1990 diseases/traits and 5349 SNPs fulfilled the criteria and were used to find associations where index SNPs of two diseases/traits were within 500 kb of each other. Overlapping risk loci were used to compute a disease-by-disease correlation matrix for every pair of diseases/traits. We selected 22 autoimmune and 19 representative non-autoimmune diseases/traits for comparison and visualization in Fig. 5a as well as summary statistics from four autoimmune diseases for in-depth locus comparison^70–73^.

We also used LD score regression to calculate genetic correlation between our GWAS results and 844 predefined traits at the LD Hub (http://ldsc.broadinstitute.org)^74^. LD score regression uses GWAS summary statistics of complex traits and diseases but excludes the HLA region, limiting its capacity for estimating genetic correlations between traits with a large proportion of their heritability explained by variation in the HLA.

### 3D models of the second PHD domain in AIRE

To visualize the associated amino acids in the HLA-DQA1 and HLA-DQB1 genes, the x-ray diffraction model of HLA-DQ2.3 (DQA1*03:01/DQB1*02:01) was used (Protein Data Bank 20 id: 4D8P)^75^. In order to generate protein models of the PHD2 domain and the potential structural impact of *AIRE* variants associated in this GWAS, we used the previously published NMR structure for PHD2 as a template (Protein Data Bank 20 id: 2LRI)^21^. This domain structure was subsequently mutated at the p.R471 position to a cysteine and visualized using PyMOL (https://pymol.org/).

### Reporting summary

Further information on research design is available in the Nature Research Reporting Summary linked to this article.

## Supplementary information

Supplementary Information

Peer Review File

Reporting Summary

Description of Additional Supplementary Files

Supplementary Data 1

## Data Availability

Summary statistics that support the findings of this study have been deposited in the NHGRI-EBI GWAS catalog with the accession code GCST90011871. The individual level genotype data are not publicly available since they contain information that could compromise research participant privacy and consent.
