# Supplementary material for: GWAS for autoimmune Addison’s disease identifies multiple risk loci and highlights AIRE in disease susceptibility
Source: Nat Commun. 2021 Feb 11;12:959. doi: 10.1038/s41467-021-21015-8 (PMC7878795; doi:10.1038/s41467-021-21015-8)
Supplement: Supplementary file 1 — Supplementary Information [file 41467_2021_21015_MOESM1_ESM.pdf]

SUPPLEMENTARY INFORMATION FOR:

GWAS for autoimmune Addison's disease identifies multiple risk loci and highlights AIRE in disease susceptibility

Table of contents

Supplementary Table 1, Demographics and comorbidities....3
Supplementary Figure 1, Principal components....4
Supplementary Figure 2, QQ-plot....6
Supplementary Figure 3, Locus plots....7
Supplementary Table 2, Clinical characteristics associated with p.R471C....11
Supplementary Table 3, Additive and recessive models for the two top-SNPs in *AIRE*....11
Supplementary Table 4, Genetic associations stratified by *AIRE* genotypes, I....12
Supplementary Table 5, Genetic associations stratified by *AIRE* genotypes, II....13
Supplementary Figure 4, 3D models of the PHD2 domain in AIRE....14
Supplementary Figure 5, *AIRE*-dependent transcription....15
Supplementary Table 6, HLA amino-acids associated to autoimmune Addison’s disease....17
Supplementary Figure 6, Effect size estimates for autoimmune diseases associated with AAD risk loci.19
Supplementary Figure 7, Side-by-side comparison of association statistics across autoimmune diseases for all AAD risk loci....20
Supplementary Table 7, Loci previously implicated in AAD....24
Supplementary Figure 8, Gene set enrichment analysis....25
Supplementary Note 1, Data processing and quality control....26
Supplementary Figure 9, Overview of the raw data processing....26
Supplementary Table 8, Raw data.... 27
Supplementary Table 9, Marker quality control, part 1....28
Supplementary Table 10, Sample quality control, part 1....29
Supplementary Table 11, Identification of samples with sex chromosome disorders....30
Supplementary Table 12, identification of global ancestry....30
Supplementary Table 13, Identification of samples from related subjects and identical samples....31
Supplementary Table 14, Marker quality control, part 2....32
Supplementary Table 15, Sample quality control, part 2....33
Supplementary Table 16, Post QC dataset....34

Supplementary Table 17, Selection of data for phasing..35
Supplementary Table 18, Data for phasing..35
Supplementary Table 19, Imputation preparation..36
Supplementary Table 20, Selection of data for GWAS..37
Supplementary Table 21, Samples eligible for association studies..38
Supplementary Table 22, The GWAS dataset..38
Supplementary Note 2, Functional assay of AIRE function..39
Supplementary Note 3, Building a model of HLA risk for autoimmune Addison's disease........................41
Supplementary Figure 10, Q-Q plots for interaction term P values..44
Supplementary References..45

Supplementary Table 1, Demographics and comorbidities

**Sex and country of residence**	
Subjects	1223 AAD cases with 21-hydroxylase autoantibodies<br>4097 blood donor controls
Sex	2764 male (52%) and 2556 female (48%)
Country of residence	2899 Swedish (54%) and 2421 Norwegian (46%)
**Associated autoimmune comorbidities in AAD cases**	
**Disease**	**Number of cases (%)**
APS-1	0 (0%), NA=0
APS-2	682 (56%), NA=0
Hypothyroidism	516 (42%), NA=24
Type 1 diabetes	147 (12%), NA=33
Vitiligo	110 (9%), NA=35
Hyperthyroidism	93 (8%), NA=34
Pernicious anemia	90 (7%), NA=42
Primary hypogonadism	53 (4%), NA=236
Celiac disease	52 (4%), NA=37
Alopecia areata	26 (2%), NA=38
Any of the above	780 (64%)

NA, not available

Supplementary Figure 1, Principal components

PCA plot displaying Eigenvectors of genetic variation. We used principal component analysis of high-quality markers with MAF > 0.05, pruned for LD ($r^2 < 0.2$), and excluding the extended HLA region (chr 6: 25.5-33.5 Mb).

Colored by nationality **Colored as cases and controls**

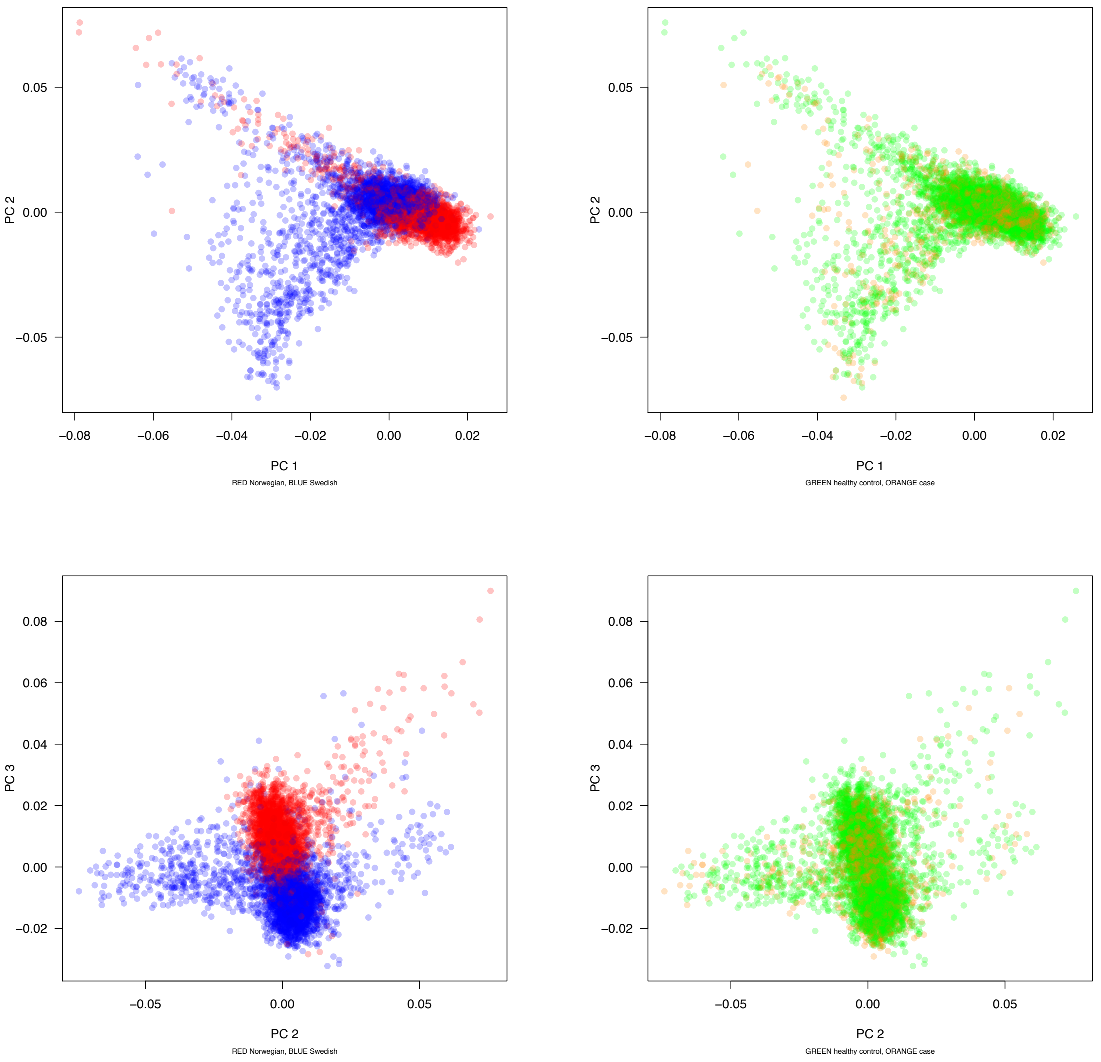

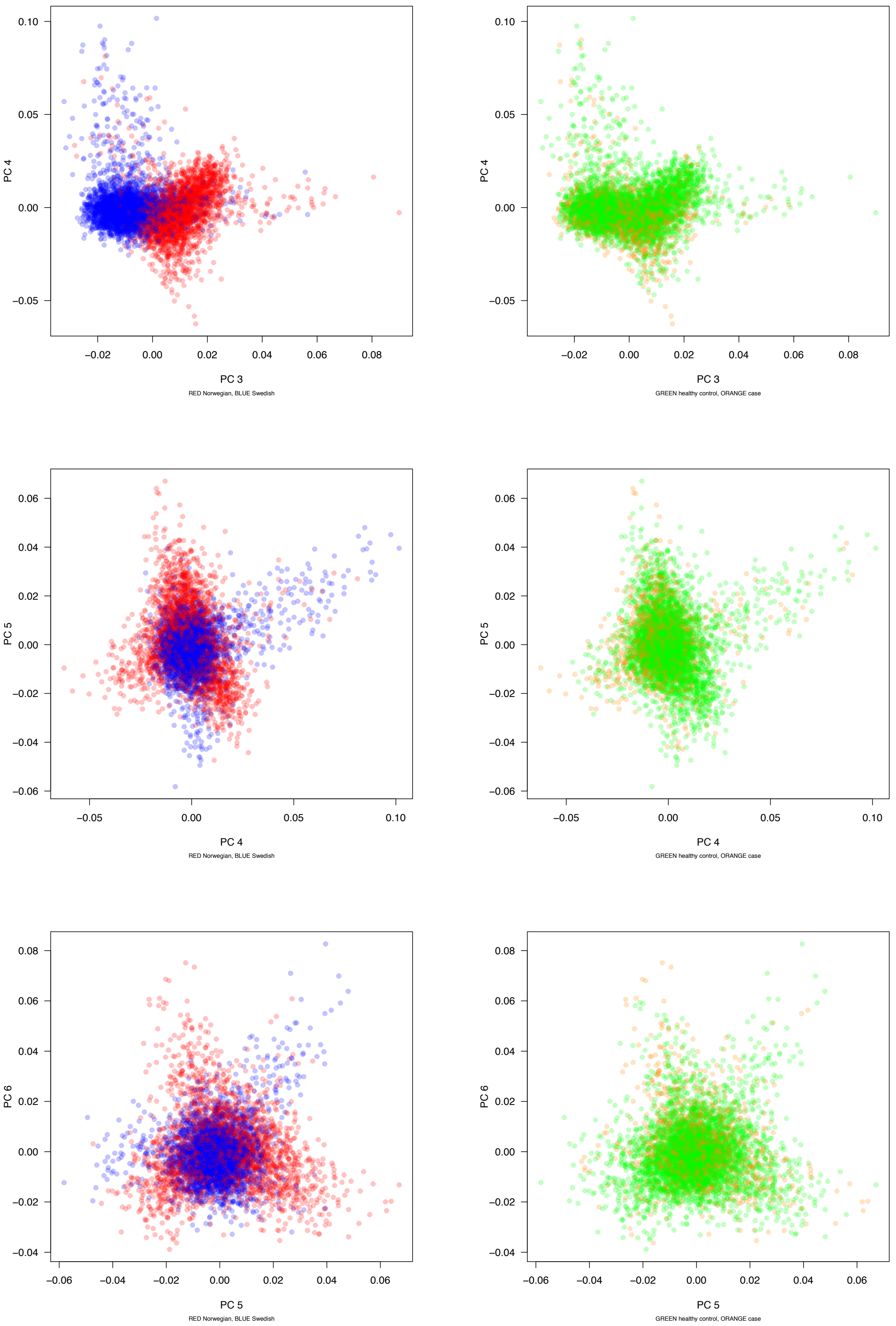
PC 4
PC 3
RED Norwegian, BLUE Swedish
PC 4
PC 3
GREEN healthy control, ORANGE case
PC 5
PC 4
RED Norwegian, BLUE Swedish
PC 5
PC 4
GREEN healthy control, ORANGE case
PC 6
PC 5
RED Norwegian, BLUE Swedish
PC 6
PC 5
GREEN healthy control, ORANGE case

Supplementary Figure 2, QQ-plot

Quantile-quantile plot displaying P values from logistic regression of allele dosages for 1,223 cases and 4,097 controls, and a random uniform distribution. The HLA region and the BACH2 locus have been excluded from the plot to increase readability. Genome-wide summary statistics uploaded to LD hub generated the following: Observed scale h^2 0.4916 (0.1218), λ_{GC} 1.0527, mean χ^2 1.0705, and LD score regression intercept: 1.0168 (0.007).

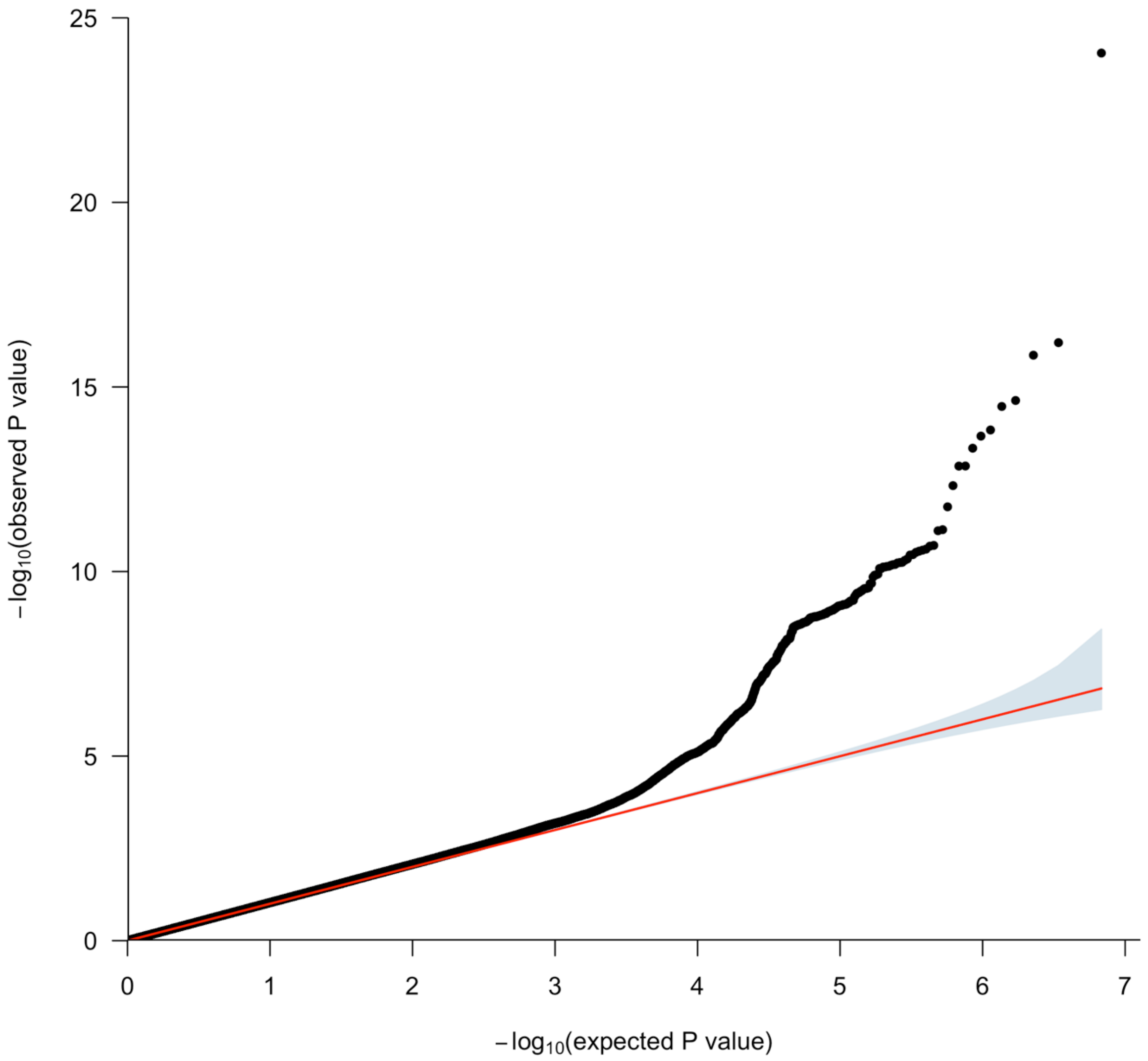

Supplementary Figure 3, Locus plots of genome-wide significant loci

Left-hand plots were made interactively from GWAS summary statistics at the LocusZoom website (http://http://locuszoom.org/)[1]. Right-hand plots were made in-house to indicate which variants were imputed or genotyped . From logistic regression in 1,223 cases diagnosed with autoimmune Addison's disease and 4,097 controls. Prioritized gene in italics: A – chromosome 1 locus (*PTPN22*), B – chromosome 2 locus (*CTLA4*), C- chromosome 3 locus (*LPP*), D – chromosome 6 HLA locus, centred on top SNP (smallest p-value), E – chromosome 6 HLA locus, entire, F – chromosome 6 locus (*BACH2*), G – chromosome 12 locus (*SH2B3*), H – chromosome 19 locus (*SIGLEC5*), I – chromosome 21 locus (*UBASH3A*), J – chromosome 21 locus (*AIRE*), primary signal, K – chromosome 21 locus (*AIRE*), secondary signal

A

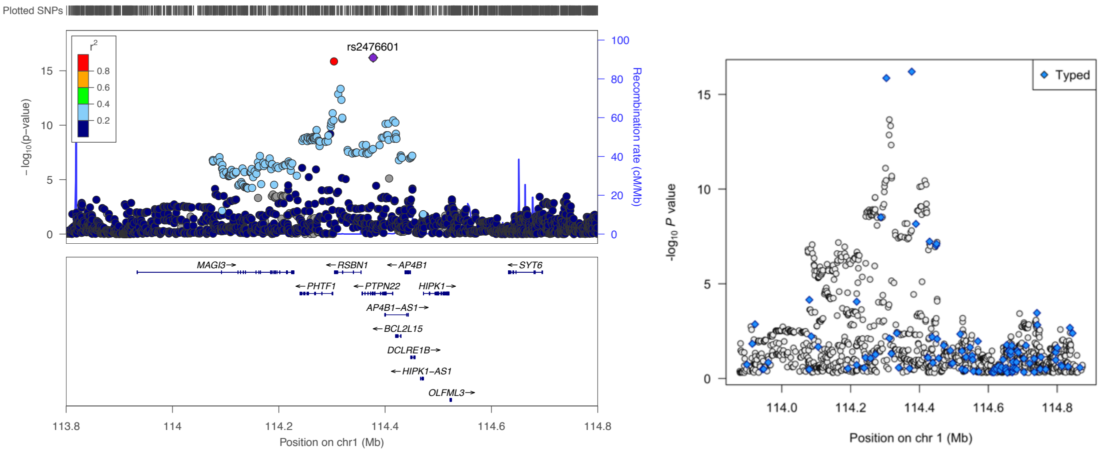

B

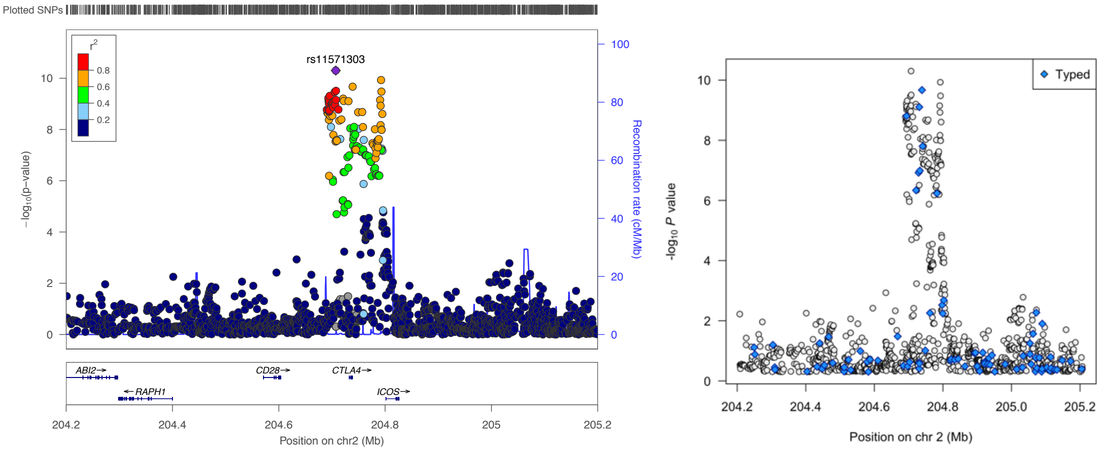

C

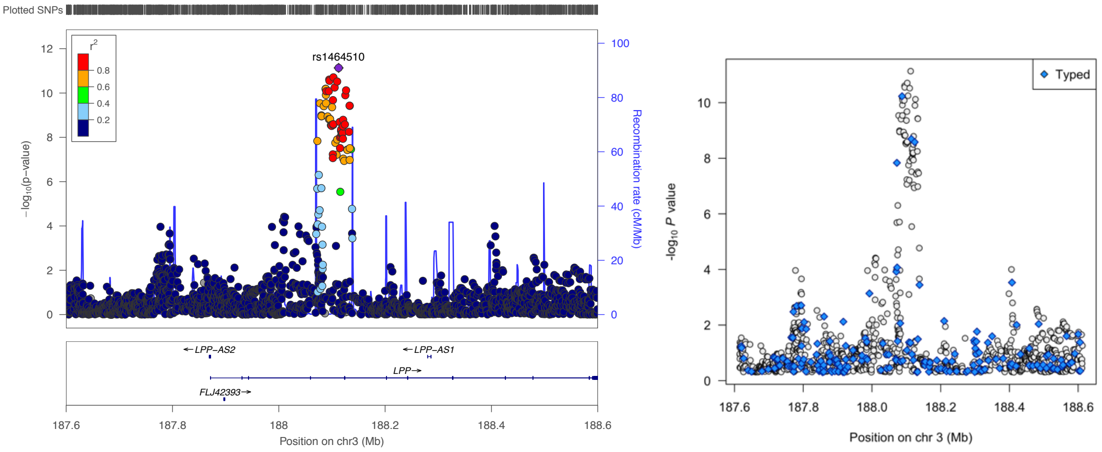

Plotted SNPs
rs1464510
r2
0.8
0.6
0.4
0.2
-log10(p-value)
Recombination rate (cM/Mb)
LPP-AS2
LPP-AS1
LPP
FLJ42393
187.6
187.8
188
188.2
188.4
188.6
Position on chr3 (Mb)
Typed
-log10 P value
Position on chr 3 (Mb)

D

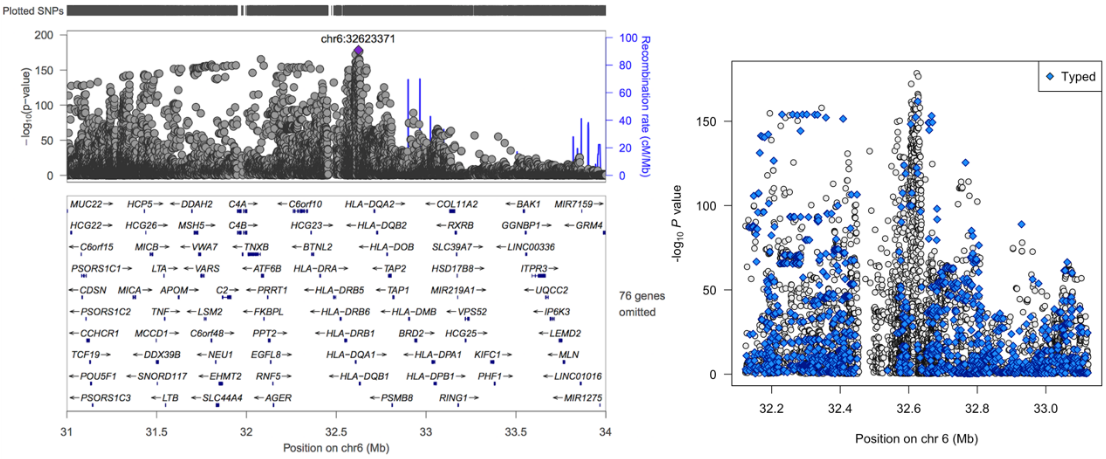

Plotted SNPs
chr6:32623371
-log10(p-value)
Recombination rate (cM/Mb)
MUC22 HCP5 DDAH2 C4A C6orf10 HLA-DQA2 COL11A2 BAK1 MIR7159
HCG22 HCG26 MSH5 C4B HCG23 HLA-DQB2 RXRB GGNBP1 GRM4
C6orf15 MICB VWA7 TNXB BTNL2 HLA-DOB SLC39A7 LINC00336
PSORS1C1 LTA VARS ATF6B HLA-DRA TAP2 HSD17B8 ITPR3
CDSN MICA APOM C2 PRRT1 HLA-DRB5 TAP1 MIR219A1 UQCC2
PSORS1C2 TNF LSM2 FKBPL HLA-DRB6 HLA-DMB VPS52 IP6K3
CCHCR1 MCCD1 C6orf48 PPT2 HLA-DRB1 BRD2 HCG25 LEMD2
TCF19 DDX39B NEU1 EGFL8 HLA-DQA1 HLA-DPA1 KIFC1 MLN
POU5F1 SNORD117 EHMT2 RNF5 HLA-DQB1 HLA-DPB1 PHF1 LINC01016
PSORS1C3 LTB SLC44A4 AGER PSMB8 RING1 MIR1275
76 genes omitted
Position on chr6 (Mb)
Typed
-log10 P value
Position on chr 6 (Mb)

E

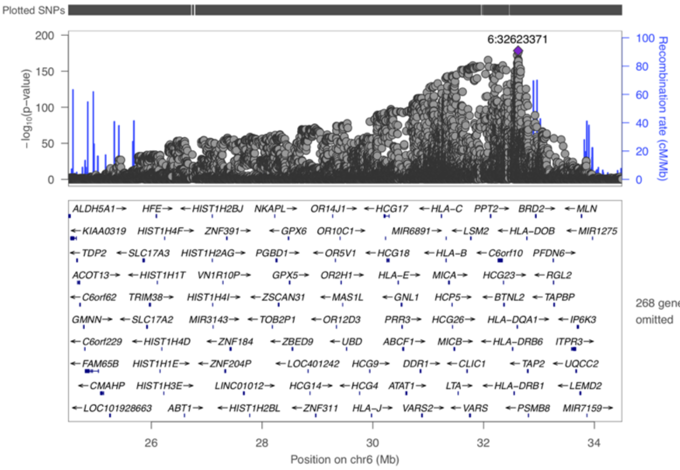

Plotted SNPs
6:32623371
-log10(p-value)
Recombination rate (cM/Mb)
ALDH5A1 HFE HIST1H2BJ NKAPL OR14J1 HCG17 HLA-C PPT2 BRD2 MLN
KIAA0319 HIST1H4F ZNF391 GPX6 OR10C1 MIR6891 LSM2 HLA-DOB MIR1275
TDP2 SLC17A3 HIST1H2AG PGBD1 OR5V1 HCG18 HLA-B C6orf10 PFDN6
ACOT13 HIST1H1T VN1R10P GPX5 OR2H1 HLA-E MICA HCG23 RGL2
C6orf62 TRIM38 HIST1H4I ZSCAN31 MAS1L GNL1 HCP5 BTNL2 TAPBP
GMNN SLC17A2 MIR3143 TOB2P1 OR12D3 PRR3 HCG26 HLA-DQA1 IP6K3
C6orf229 HIST1H4D ZNF184 ZBED9 UBD ABCF1 MICB HLA-DRB6 ITPR3
FAM65B HIST1H1E ZNF204P LOC401242 HCG9 DDR1 CLIC1 TAP2 UQCC2
CMAHP HIST1H3E LINC01012 HCG14 HCG4 ATAT1 LTA HLA-DRB1 LEMD2
LOC101928663 ABT1 HIST1H2BL ZNF311 HLA-J VARS2 VARS PSMB8 MIR7159
268 genes omitted
Position on chr6 (Mb)

F

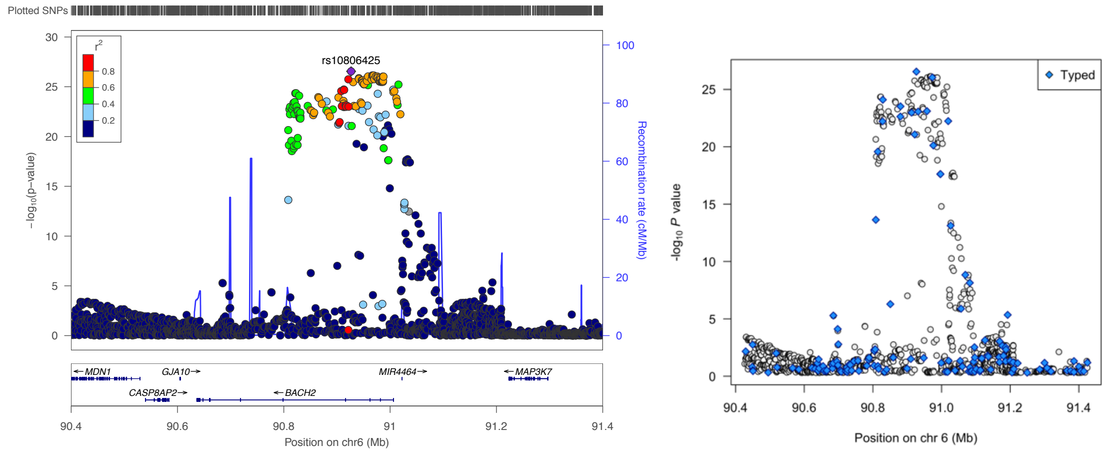
Plotted SNPs
rs10806425
r2
0.8
0.6
0.4
0.2
-log10(p-value)
Recombination rate (cM/Mb)
MDN1
GJA10
MIR4464
MAP3K7
CASP8AP2
BACH2
Position on chr6 (Mb)
Typed
-log10 P value
Position on chr 6 (Mb)

G

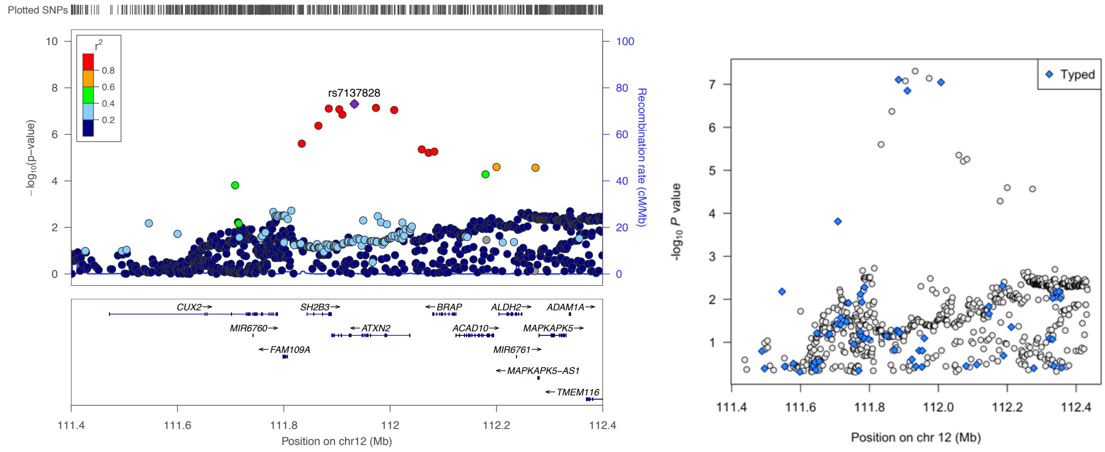
Plotted SNPs
rs7137828
r2
0.8
0.6
0.4
0.2
-log10(p-value)
Recombination rate (cM/Mb)
CUX2
SH2B3
BRAP
ALDH2
ADAM1A
MIR6760
ATXN2
ACAD10
MAPKAPK5
FAM109A
MIR6761
MAPKAPK5-AS1
TMEM116
Position on chr12 (Mb)
Typed
-log10 P value
Position on chr 12 (Mb)

H

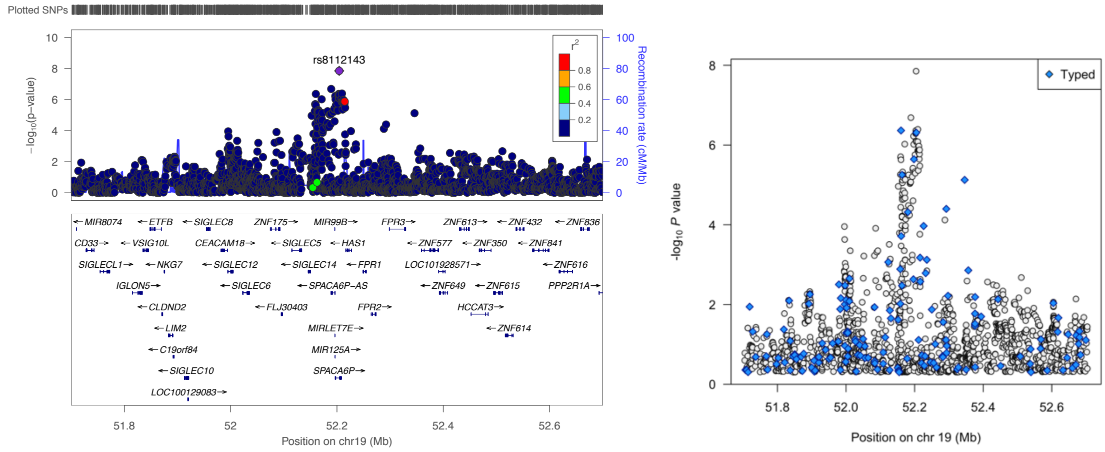
Plotted SNPs
rs8112143
r2
0.8
0.6
0.4
0.2
-log10(p-value)
Recombination rate (cM/Mb)
MIR8074
ETFB
SIGLEC8
ZNF175
MIR99B
FPR3
ZNF613
ZNF432
ZNF836
CD33
VSIG10L
CEACAM18
SIGLEC5
HAS1
ZNF577
ZNF350
ZNF841
SIGLECL1
NKG7
SIGLEC12
SIGLEC14
FPR1
LOC101928571
ZNF616
IGLON5
SIGLEC6
SPACA6P-AS
ZNF649
ZNF615
PPP2R1A
CLDND2
FLJ30403
FPR2
HCCAT3
LIM2
MIRLET7E
ZNF614
C19orf84
MIR125A
SIGLEC10
SPACA6P
LOC100129083
Position on chr19 (Mb)
Typed
-log10 P value
Position on chr 19 (Mb)

I

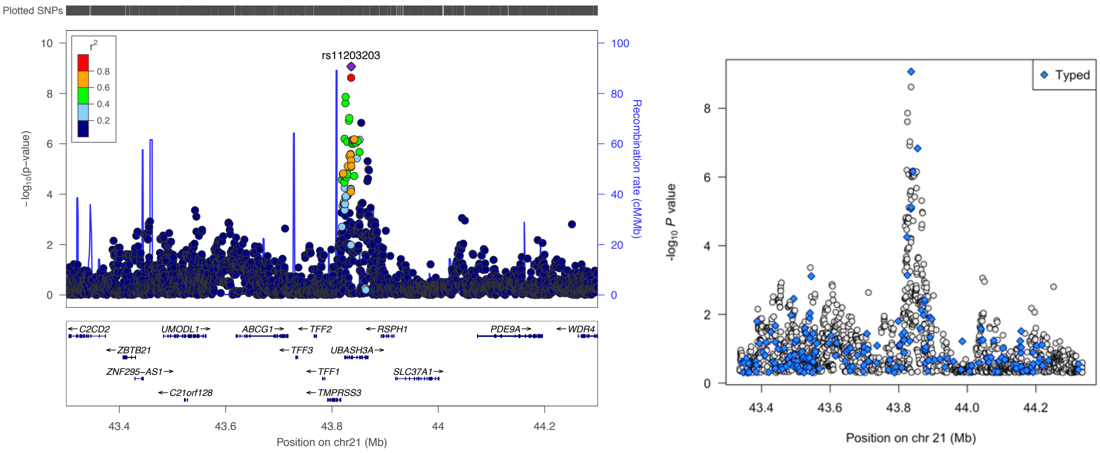

Plotted SNPs
rs11203203
r²
0.8
0.6
0.4
0.2
− log10(p−value)
Recombination rate (cM/Mb)
C2CD2
UMODL1
ABCG1
TFF2
RSPH1
PDE9A
WDR4
ZBTB21
TFF3
UBASH3A
ZNF295−AS1
TFF1
SLC37A1
C21orf128
TMPRSS3
Position on chr21 (Mb)
Typed
-log10 P value
Position on chr 21 (Mb)

J

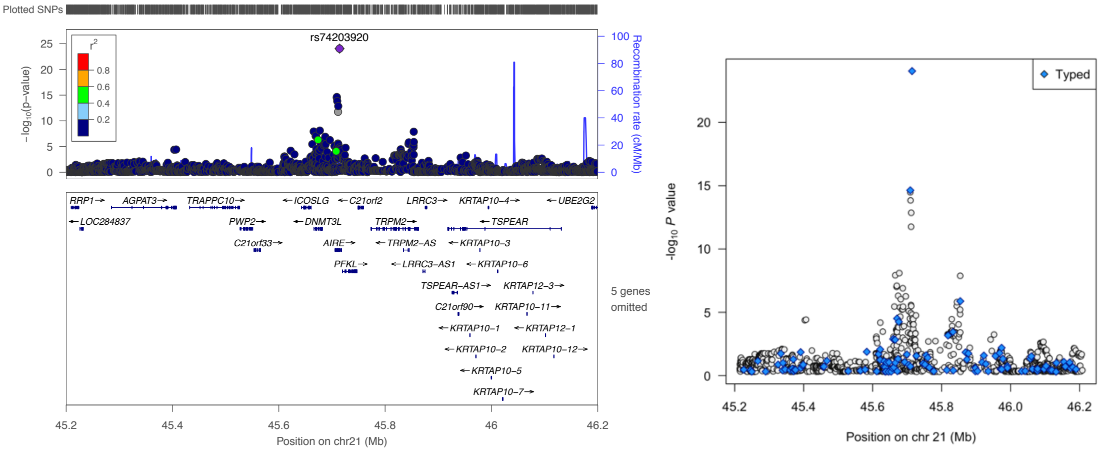

Plotted SNPs
rs74203920
r²
0.8
0.6
0.4
0.2
− log10(p−value)
Recombination rate (cM/Mb)
RRP1
AGPAT3
TRAPPC10
ICOSLG
C21orf2
LRRC3
KRTAP10−4
UBE2G2
LOC284837
PWP2
DNMT3L
TRPM2
TSPEAR
C21orf33
AIRE
TRPM2−AS
KRTAP10−3
PFKL
LRRC3−AS1
KRTAP10−6
TSPEAR−AS1
KRTAP12−3
C21orf90
KRTAP10−11
KRTAP10−1
KRTAP12−1
KRTAP10−2
KRTAP10−12
KRTAP10−5
KRTAP10−7
5 genes omitted
Position on chr21 (Mb)
Typed
-log10 P value
Position on chr 21 (Mb)

K

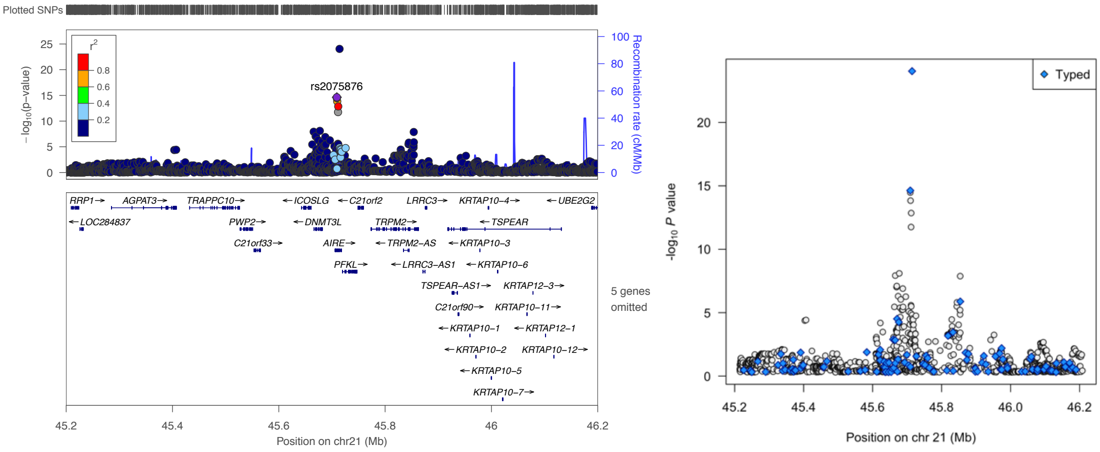

Plotted SNPs
rs2075876
r²
0.8
0.6
0.4
0.2
− log10(p−value)
Recombination rate (cM/Mb)
RRP1
AGPAT3
TRAPPC10
ICOSLG
C21orf2
LRRC3
KRTAP10−4
UBE2G2
LOC284837
PWP2
DNMT3L
TRPM2
TSPEAR
C21orf33
AIRE
TRPM2−AS
KRTAP10−3
PFKL
LRRC3−AS1
KRTAP10−6
TSPEAR−AS1
KRTAP12−3
C21orf90
KRTAP10−11
KRTAP10−1
KRTAP12−1
KRTAP10−2
KRTAP10−12
KRTAP10−5
KRTAP10−7
5 genes omitted
Position on chr21 (Mb)
Typed
-log10 P value
Position on chr 21 (Mb)

Supplementary Table 2, Clinical characteristics associated with p.R471C

Fractions were tested with Pearson's chi square test, and average age of onset with t-test. Subjects homozygote for p.R471C were left out due to small sample size (n=4).

**Parameter**	**Homozygous p.R471 (n=1068)**	**Heterozygous p.R471C (n=151)**	**P value**
Age of onset (mean)	34.8	32.6	0.079
Female	62.6%	59.6%	0.30
APS-2	54.7%	50.3%	0.32
Hypothyroidism	43.4%	40.7%	0.60
Type 1 diabetes	12.8%	10.4%	0.42
Vitiligo	9.3%	9.7%	0.88
Hyperthyroidism	8.0%	6.9%	0.67
Pernicious anemia	7.7%	7.0%	0.75
Primary hypogonadism	5.3%	6.1%	0.70

Supplementary Table 3, Additive and recessive models for the two top-SNPs in *AIRE*

The risk effects of rs74203920 and/or rs2075876 were best described by additive models, and we found no support for recessive inheritance of AAD linked to these SNPs. Odds ratios and P values were estimated using logistic regression in 1,223 cases diagnosed with autoimmune Addison's disease and 4,097 controls.

	**Genotype frequency [a]**						**Risk allele frequency**					
	**Cases**			**Controls**			**Cases**	**Controls**	**Additive model**		**Recessive model**	
**SNP**	**AA**	**Aa**	**aa**	**AA**	**Aa**	**aa**	**a**	**a**	**OR (95% CI)**	**P**	**OR (95% CI)**	**P**
rs74203920	0.8724	0.1243	0.0033	0.9607	0.0391	0.0002	0.065	0.020	3.42 (2.7-4.3)	9×10^{-25}	10.36 (1.1-95.7)	0.039
rs2075876	0.0025	0.0899	0.9076	0.0112	0.1860	0.8028	0.950	0.900	2.27 (1.9-2.8)	2.32×10^{-15}	4.17 (1.3-13.5)	0.018
rs2075876, conditioned on the genotypes of rs74203920									2.17 (1.8-2.7)	7.8×10^{-14}	4.16 (1.3-13.5)	0.018

[a] AA: homozygote for non-risk allele, Aa: heterozygote, aa: homozygote for risk allele.

Supplementary Table 4, Genetic associations stratified by *AIRE* genotypes, I

In association analysis of 156 cases carrying rs74203920-p.R471C versus 1067 cases that do not, no differential association to the GWAS-associated loci could be found, in line with the independent inheritance of these loci. rs2075876 could not be analyzed in the same way due to the low number of cases not carrying the risk allele. Odds ratios and P values were estimated using logistic regression

			Allele frequency[c]			
**Locus[a]**	**SNP**	**Risk/alt allele[b]**	**Carriers**	**Non-carriers**	**OR (95% CI)**	**P**
PTPN22	rs2476601	A/G	0.20	0.17	1.22 (0.99-1.49)	0.2
CTLA4	rs11571303	G/A	0.64	0.69	0.79 (0.62-1.01)	0.064
LPP	rs1464510	A/C	0.51	0.51	1 (0.80-1.26)	0.95
HLA-DQB1	rs3998178	T/C	0.50	0.51	0.89 (0.67-1.17)	0.40
BACH2	rs10806425	A/C	0.50	0.50	0.98 (0.77-1.24)	0.86
SH2B3	rs7137828	C/T	0.57	0.52	1.24 (0.97-1.57)	0.082
SIGLEC5	rs8112143	A/G	0.072	0.073	1.02 (0.60-1.72)	0.95
UBASH3A	rs11203203	A/G	0.37	0.43	0.77 (0.6-0.99)	0.042
AIRE	rs74203920	T/C	0.51	0.00	-	-
AIRE	rs2075876	G/A	0.96	0.95	1.4 (0.74-2.63)	0.30

[a] Reported genetic variants and prioritized genes in the main GWAS.

[b] The risk allele indicates the effect allele for the OR in the main GWAS, the second position gives the alternative allele.

[c] 156 rs74203920-p.R471C carriers with AAD, and 1067 non-carrier AAD cases.

Supplementary Table 5, Genetic associations stratified by *AIRE* genotypes, II

This table presents in parallel the results from two association tests: Association analysis of 156 cases carrying rs74203920-p.R471C versus healthy controls, as well as 1067 cases not carrying rs74203920-p.R471C versus healthy controls. The overlapping effect estimates show no differential association to the GWAS-associated loci, in line with the independent inheritance of these loci. Odds ratios and P values were estimated using logistic regression

			Allele frequency[c]				Allele frequency[d]			
**Locus[a]**	**SNP**	**Risk/alt allele[b]**	**p.R471C+**	**Healthy controls**	**OR (95% CI)**	**P**	**p.R471C-**	**Healthy controls**	**OR (95% CI)**	**P**
PTPN22	rs2476601	A/G	0.20	0.11	2.05 (1.52-2.76)	2.4×10^{-6}	0.17	0.11	1.7 (1.48-1.95)	1.9×10^{-14}
CTLA4	rs11571303	G/A	0.64	0.61	1.12 (0.89-1.42)	0.33	0.69	0.61	1.43 (1.3-1.59)	1.4×10^{-11}
LPP	rs1464510	A/C	0.51	0.42	1.38 (1.1-1.72)	5×10^{-3}	0.51	0.42	1.37 (1.24-1.5)	8.6×10^{-11}
HLA-DQB1	rs3998178	T/C	0.50	0.19	5.4 (4.12-7.08)	2.6×10^{-34}	0.51	0.19	6.01 (5.29-6.83)	8.9×10^{-166}
BACH2	rs10806425	A/C	0.50	0.37	1.64 (0.77-1.24)	2.7×10^{-5}	0.50	0.37	1.69 (1.53-1.86)	5.9×10^{-25}
SH2B3	rs7137828	C/T	0.57	0.46	1.55 (1.23-1.96)	2.4×10^{-4}	0.52	0.46	1.26 (1.14-1.4)	3.6×10^{-6}
SIGLEC5	rs8112143	A/G	0.072	0.047	1.87 (1.11-3.16)	0.018	0.073	0.047	1.87 (1.49-2.35)	6.2×10^{-8}
UBASH3A	rs11203203	A/G	0.37	0.35	1.08 (0.85-1.37)	0.52	0.43	0.35	1.39 (1.26-1.53)	1.3×10^{-10}
AIRE	rs74203920	T/C	0.51	0.02	-	-	0.00	0.02	-	-
AIRE	rs2075876	G/A	0.96	0.90	3.04 (1.65-5.6)	3.6×10^{-4}	0.95	0.90	2.18 (1.76-2.7)	4.5×10^{-13}

[a] Reported genetic variants and prioritized genes in the main GWAS.

[b] The risk allele indicates the effect allele for the OR in the main GWAS, the second position gives the alternative allele.

[c] 156 rs74203920-R471C carriers with AAD and 4097 healthy controls.

[d] 1067 rs74203920-R471C non-carriers with AAD and 4097 healthy controls.

Supplementary Figure 4, 3D models of the PHD2 domain in AIRE

3D models of the PHD2 domain. Mesh model of the PHD2 domain with zinc ions in teal. A - the wildtype arginine residue in the 471 position has a surface in green. B - the variant with a cysteine residue in the 471 position with a surface in red.

A. p.R471

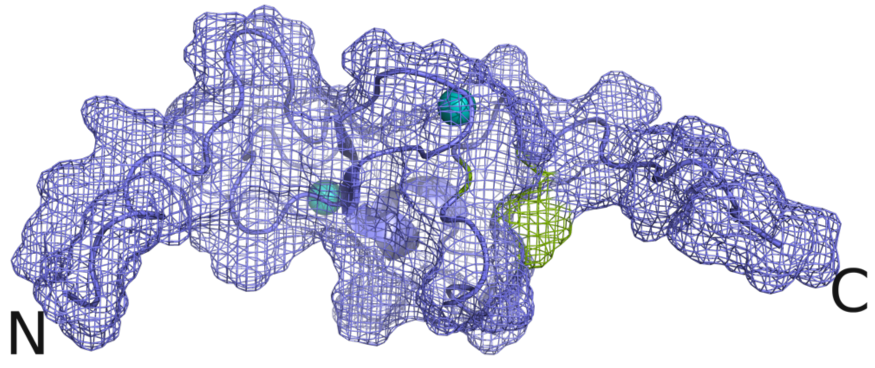

B. p.R471C

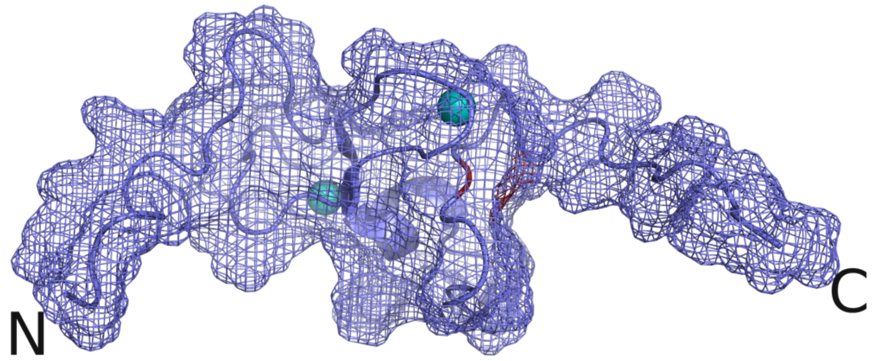

Supplementary Figure 5, *AIRE*-dependent transcription

We transfected HEK293FT cells with AIRE variants p.R471C, p.S278R, and the known deleterious variant p.R257X, and evaluated the transcription of genes with known AIRE-dependent expression; *KRT14* and *S100A8*, and *AIRE* itself. As previously shown, the p.R257X-mutation demonstrated reduced AIRE-dependent transcription when compared to wildtype. In contrast, p.R471C and p.S278R did not interfere with AIRE-dependent transcription of the tested genes.

These results are commensurate with the polymorphic status of these variants in the general population. However, the lack of effect of either rs74203920 or rs1800520 on AIRE-dependent gene expression in this assay could also have other interpretations. First, because AIRE is predominantly expressed and exerts its main functions in the thymus, the renal origin of the selected cell line may not be relevant for AIRE, or not relevant for the aspect of AIRE function that is altered by the studied genetic variation. With the linkage disequilibrium surrounding the associated SNPs, it is also possible that other nearby genes are involved.

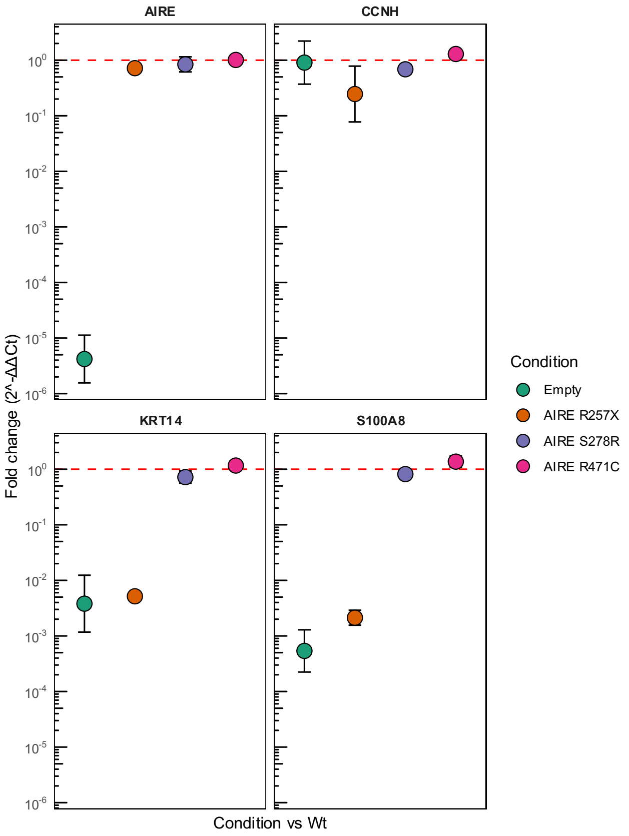

RNA from cells transfected with empty pSF-UB plasmid or pSF-UB plasmids containing wildtype *AIRE* or the *AIRE* mutants p.R257X, p.S278R and p.R471C were analyzed using qPCR. TaqMan probes were used for *AIRE*, the negative control gene *CCNH*, and the purported AIRE regulated genes *KRT14* and *S100A8*. p.R257X was used as a known mutation with negative effect. Results were normalized against the housekeeping gene *GAPDH*. The ΔΔCt method was used to compare the AIRE mutants and empty plasmid with the AIRE wildtype and the difference of expression was calculated as Fold Change (2^- ΔΔCt), the plotted centre being the average of biological replicate measures with the error bars showing 95% confidence interval. These are the results of two independent experiments each with three biological replicates and three technical controls, all showing the same trend. The red dashed line denotes the reference *AIRE* wildtype population expression levels at 10^0. Using two-sided ANOVA with Tukey multiple comparisons of means, the expression of *AIRE* in all *AIRE* transfected populations was significantly different from Empty (FDR < 0.001). In the expression of *CCNH* no population was significantly different from *AIRE* wildtype or Empty, although *AIRE* p.R257X was significantly different from *AIRE* p.R471C (FDR < 0.05). Finally, in the AIRE target genes *KRT14* and *S100A8* both AIRE p.S278R and p.R471C populations were significantly different from Empty and *AIRE* R257X (FDR < 0.001), but not significantly different from each other or *AIRE* wildtype.

Supplementary Table 6, HLA amino-acids associated to autoimmune Addison's disease **Odds ratios and P values were estimated using stepwise logistic regression in 1,224 cases and 4,097 controls. Only results with P<5e-8 were reported to adjust for multiple testing.**

Regression	Amino-acid	Frequency [a] Cases	Controls	Association OR (95% CI)[b]	P value[c]	HLA alleles in LD[d]
Step 1 – Baseline model						
	DQA1 pos. 52 Arg	78.9	44.1	7.82 (6.73-9.1)	2.2×10^{-157}	DQB1*02:01 (r^2 = 0.23), DQA1*05:01 (r^2 = 0.23)
	DQB1 pos. 57 Ala	66.9	31.3	4.25 (3.82-4.73)	2.6×10^{-152}	DQB1*02:01 (r^2 = 0.45), DQA1*05:01 (r^2 = 0.45)
	DRB1 pos. 77 Thr	52.5	76.4	0.13 (0.11-0.15)	8.0×10^{-148}	C*07:01 (r^2 > 0.5), B*08:01 (r^2 > 0.5), DQB1*02:01 (r^2 > 0.8), DQA1*05:01 (r^2 > 0.8), DRB1*03:01 (r^2 > 0.8)
Step 2 – Conditioned on DQB1*02:01						
	DQA1 pos. 52 Arg	78.9	44.1	4.53 (3.83-5.35)	5.1×10^{-71}	DQB1*02:01 (r^2 = 0.23), DQA1*05:01 (r^2 = 0.23)
	DQB1 pos. 30 Tyr	53.9	52.2	5.33 (4.39-6.47)	2.6×10^{-64}	DQB1*06:02 (r^2 = 0.16), DRB1*15:01 (r^2 = 0.16)
	DRB1 pos. 33 Asn	62.1	71.8	0.31 (0.27-0.35)	2.1×10^{-61}	DRB1*04:01 (r^2 > 0.5), DQA1*03:01 (r^2 > 0.5), DQB1*03:02 (r^2 > 0.5)
Step 3 – Conditioned on DQB1*02:01, and 03:02						
	DQB1 pos. 30 Tyr	53.9	52.2	3.62 (2.95-4.44)	3.8×10^{-35}	DQB1*06:02 (r^2 = 0.16), DRB1*15:01 (r^2 = 0.16)
	B pos. 74 Asp	61.5	36.5	2.26 (1.99-2.58)	1.5×10^{-34}	B*08:01 (r^2 = 0.3), C*07:02 (r^2 = 0.23)
	B pos. 45 Glu	61.7	37.2	2.21 (1.94-2.51)	7.9×10^{-33}	B*08:01 (r^2 = 0.29), C*07:02 (r^2 = 0.22)
Step 4 - Conditioned on DQB1*02:01, 03:02, and pos. 30 Y						
	B pos. 74 Asp	61.5	36.5	1.99 (1.74-2.28)	2.0×10^{-23}	B*08:01 (r^2 = 0.3), C*07:02 (r^2 = 0.23)
	B pos. 116 Ser	9.8	23.2	0.41 (0.34-0.49)	2.1×10^{-23}	B*15:01 (r^2 = 0.45), B*35:01 (r^2 = 0.18)
	B pos. 45 Glu	61.7	37.2	1.95 (1.7-2.23)	3.1×10^{-22}	B*08:01 (r^2 = 0.29), C*07:02 (r^2 = 0.22)
Step 5 - Conditioned on DQB1*02:01, 03:02, pos. 30 Y, and B pos. 74 D						
	DRB1 pos. 86 V	68.9	42.9	1.82 (1.56-2.13)	3.9×10^{-14}	DRB1*03:01 (r^2 = 0.23), DQB1*02:01 (r^2 = 0.23)
	B pos. 116 Ser	9.8	23.2	0.52 (0.43-0.63)	5.3×10^{-11}	B*15:01 (r^2 = 0.45), B*35:01 (r^2 = 0.18)
	B pos. 45 Met	6.6	15.1	0.48 (0.39-0.6)	8.7×10^{-11}	B*15:01 (r^2 > 0.5), C*03:03 (r^2 = 0.2)
Step 6 - Conditioned on DQB1*02:01, 03:02, pos. 30 Y, B pos. 74 D, and DRB1*04:04						
	B pos. 156 Asp	46.4	24.8	1.66 (1.43-1.94)	9.5×10^{-11}	B*08:01 (r^2 > 0.5), C*07:01 (r^2 = 0.41)
	C pos. 91 Gly	96.2	91.0	2.68 (1.89-3.79)	3.4×10^{-8}	C*07:01 (r^2 = 0.02), B*08:01 (r^2 = 0.02)
	DRB1 pos. 10 Tyr	49.3	37.0	2.31 (1.71-3.11)	3.6×10^{-8}	DRB1*03:01 (r^2 = 0.2), DQB1*02:01 (r^2 = 0.2)

[a] Amino acid frequency.
[b] Estimated odds ratio from the stepwise regression, i.e. not from the full model of HLA risk.
[c] P value from the stepwise regression, i.e. not from the full model of HLA risk.

[d] HLA alleles with LD $r^2 > 0.5$ are presented in categories of $r^2 > 0.5$, > 0.8, > 0.9, and > 0.95. Maximum 2 HLA alleles with $r^2 \leq 0.5$ are presente

Supplementary Figure 6, Effect size estimates for autoimmune diseases associated with AAD risk alleles.

Effect size estimates for autoimmune diseases associated with AAD risk alleles were retrieved from PhenoScanner [2 3]. The results are summarized in the heatmap below. For all autoimmune diseases, except Crohn's disease, the risk allele in *PTPN22* was consistent across the investigated diseases. We also note the inverse effect of rs2075876-G on the risk of AAD and rheumatoid arthritis, respectively. The allele that increases the susceptibility to AAD (rs2075876-G), appears associated with decreased risk of rheumatoid arthritis.

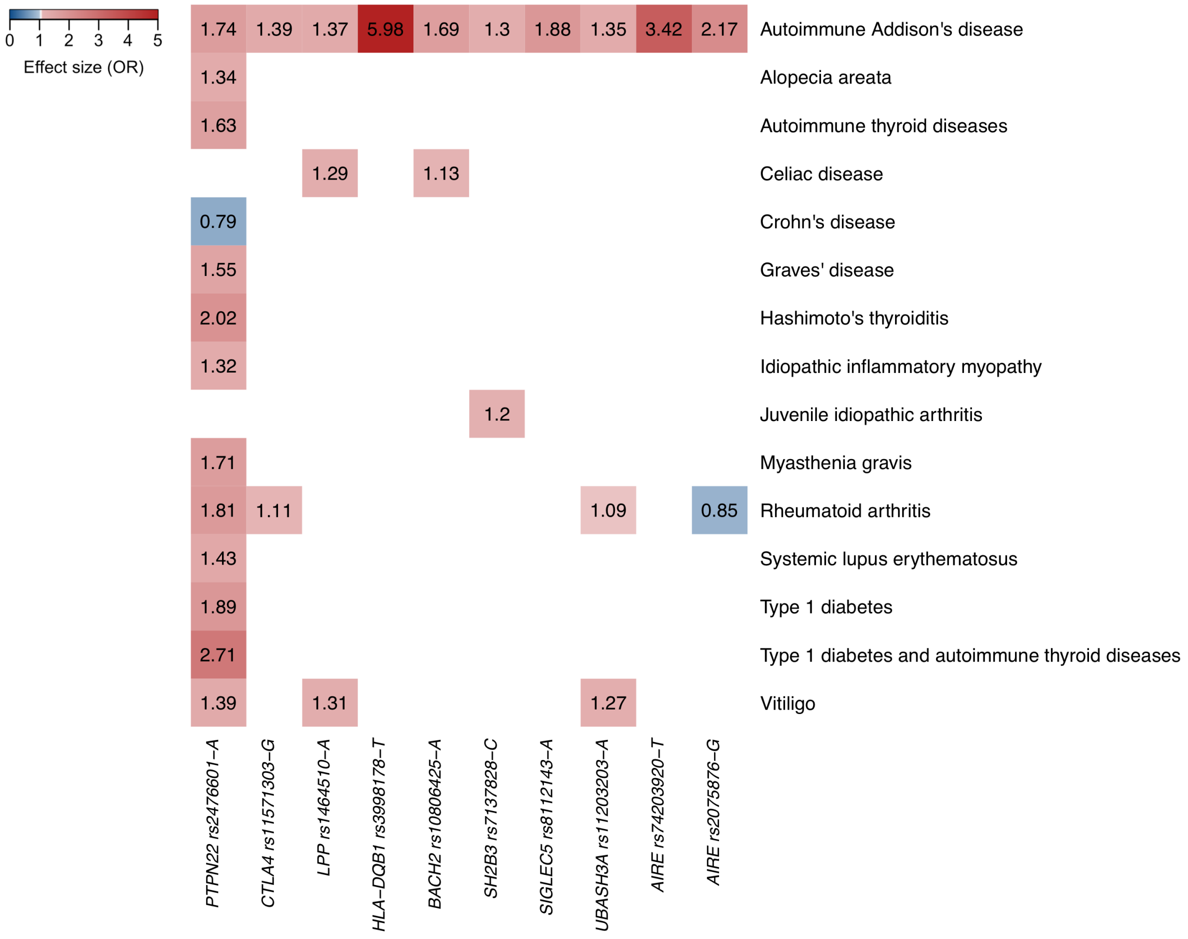

Supplementary Figure 7, Side-by-side comparison of association statistics across autoimmune diseases for all AAD risk loci.

Association signals visualized for autoimmune diseases with GWAS and/or immunochip summary statistics available through the National Human Genome Research Institute (NHGRI) - European Bioinformatics Institute (EBI) GWAS catalog, as of August 21, 2020. Plots appear on the following pages.

Summary statistics from the GWAS catalog included GCST000612, GCST001198, GCST001255, GCST002548, GCST003044, GCST003129, GCST004030, GCST004785, GCST005524, GCST005527, GCST005528, GCST005529, GCST005536, GCST005569, GCST005831, and GCST006867. Statistical methods used for the referred case-control studies may vary from study to study. The genome-wide cutoff (dashed horizontal) is set at 5e-8 to adjust for multiple testing.

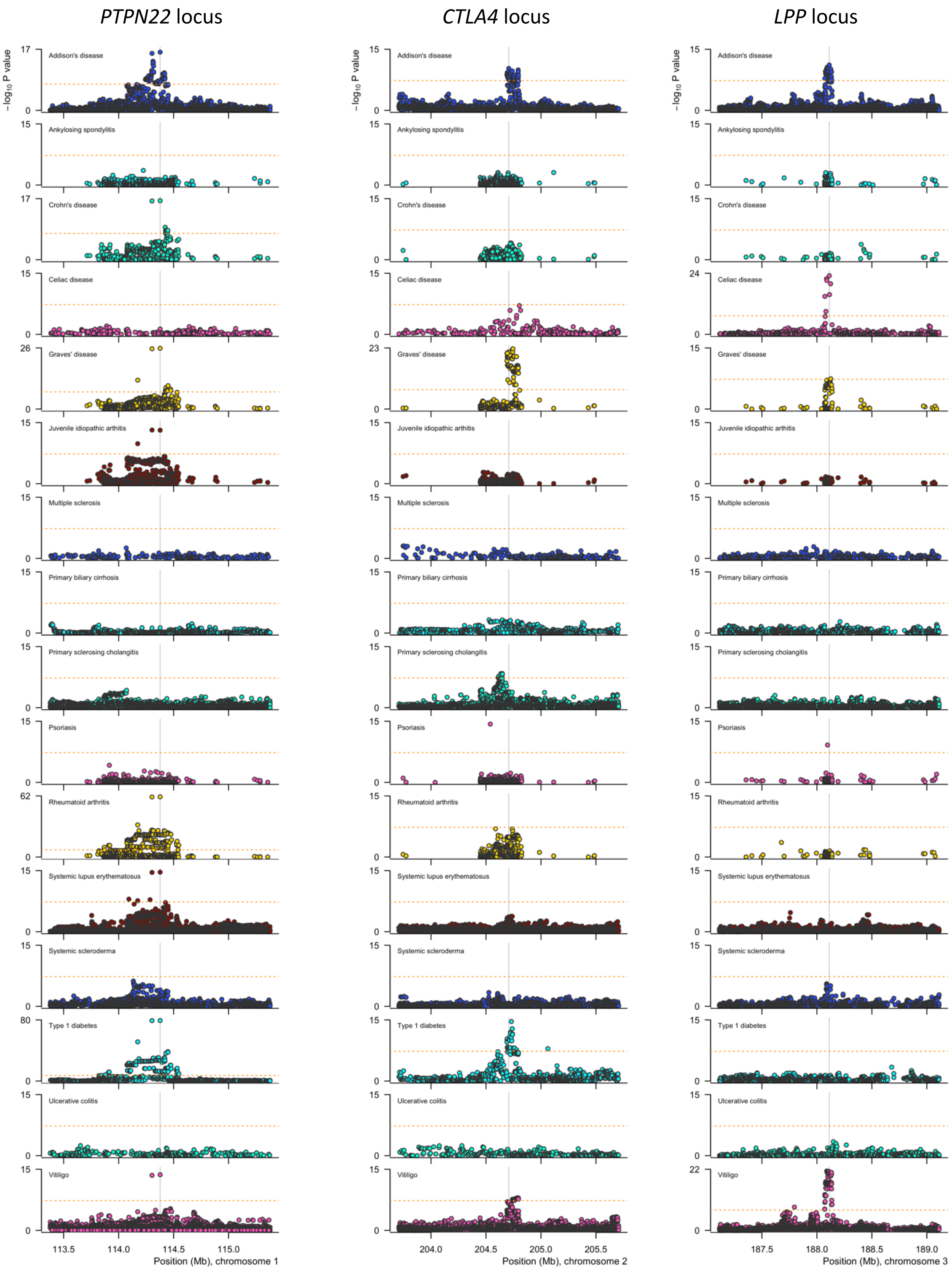

PTPN22 locus
CTLA4 locus
LPP locus
−log10 P value
Addison's disease
Ankylosing spondylitis
Crohn's disease
Celiac disease
Graves' disease
Juvenile idiopathic arthritis
Multiple sclerosis
Primary biliary cirrhosis
Primary sclerosing cholangitis
Psoriasis
Rheumatoid arthritis
Systemic lupus erythematosus
Systemic scleroderma
Type 1 diabetes
Ulcerative colitis
Vitiligo
Position (Mb), chromosome 1
Position (Mb), chromosome 2
Position (Mb), chromosome 3

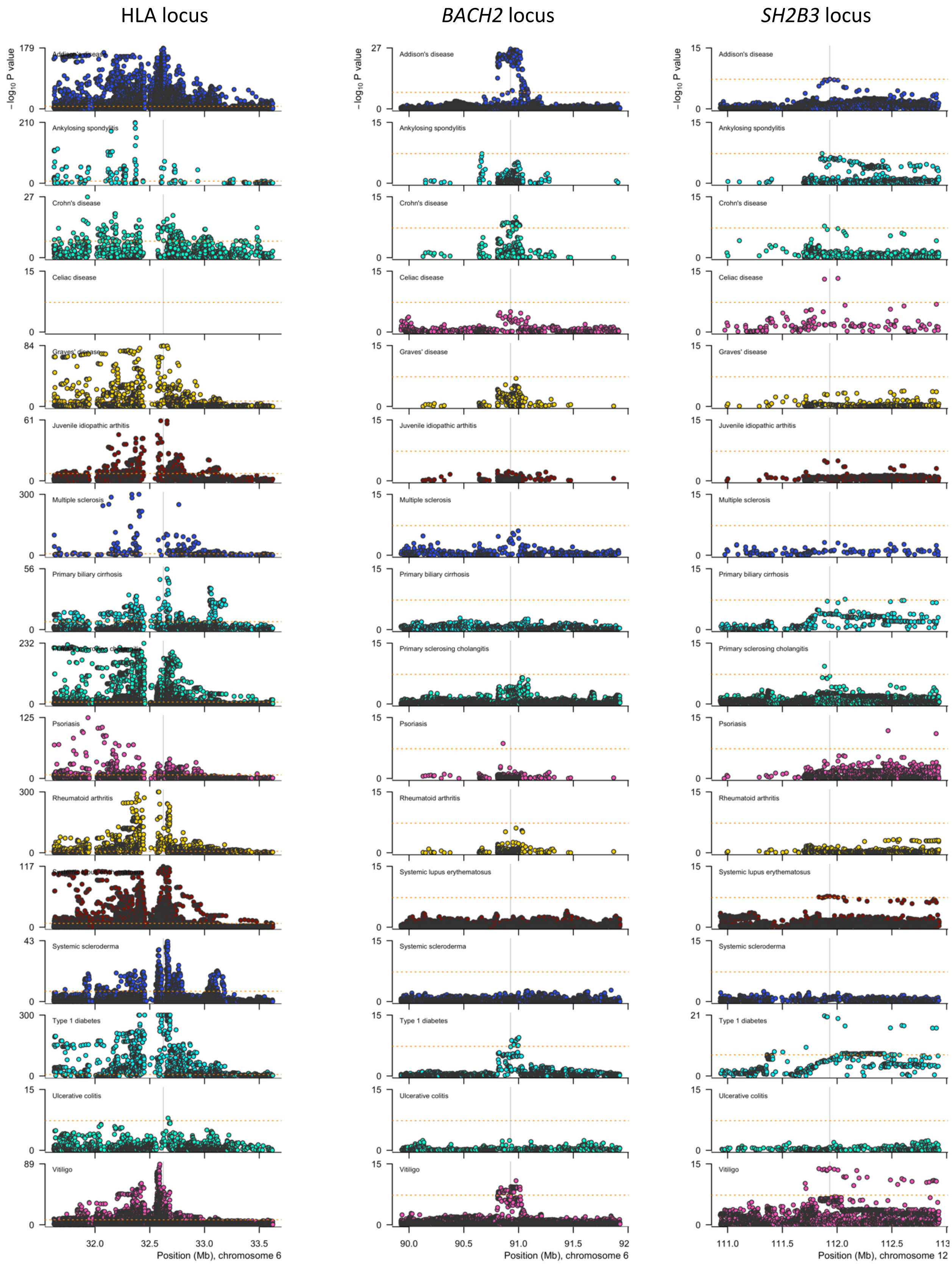
HLA locus
BACH2 locus
SH2B3 locus
$-\log_{10}$ P value
Addison's disease
Ankylosing spondylitis
Crohn's disease
Celiac disease
Graves' disease
Juvenile idiopathic arthritis
Multiple sclerosis
Primary biliary cirrhosis
Primary sclerosing cholangitis
Psoriasis
Rheumatoid arthritis
Systemic lupus erythematosus
Systemic scleroderma
Type 1 diabetes
Ulcerative colitis
Vitiligo
Position (Mb), chromosome 6
Position (Mb), chromosome 6
Position (Mb), chromosome 12

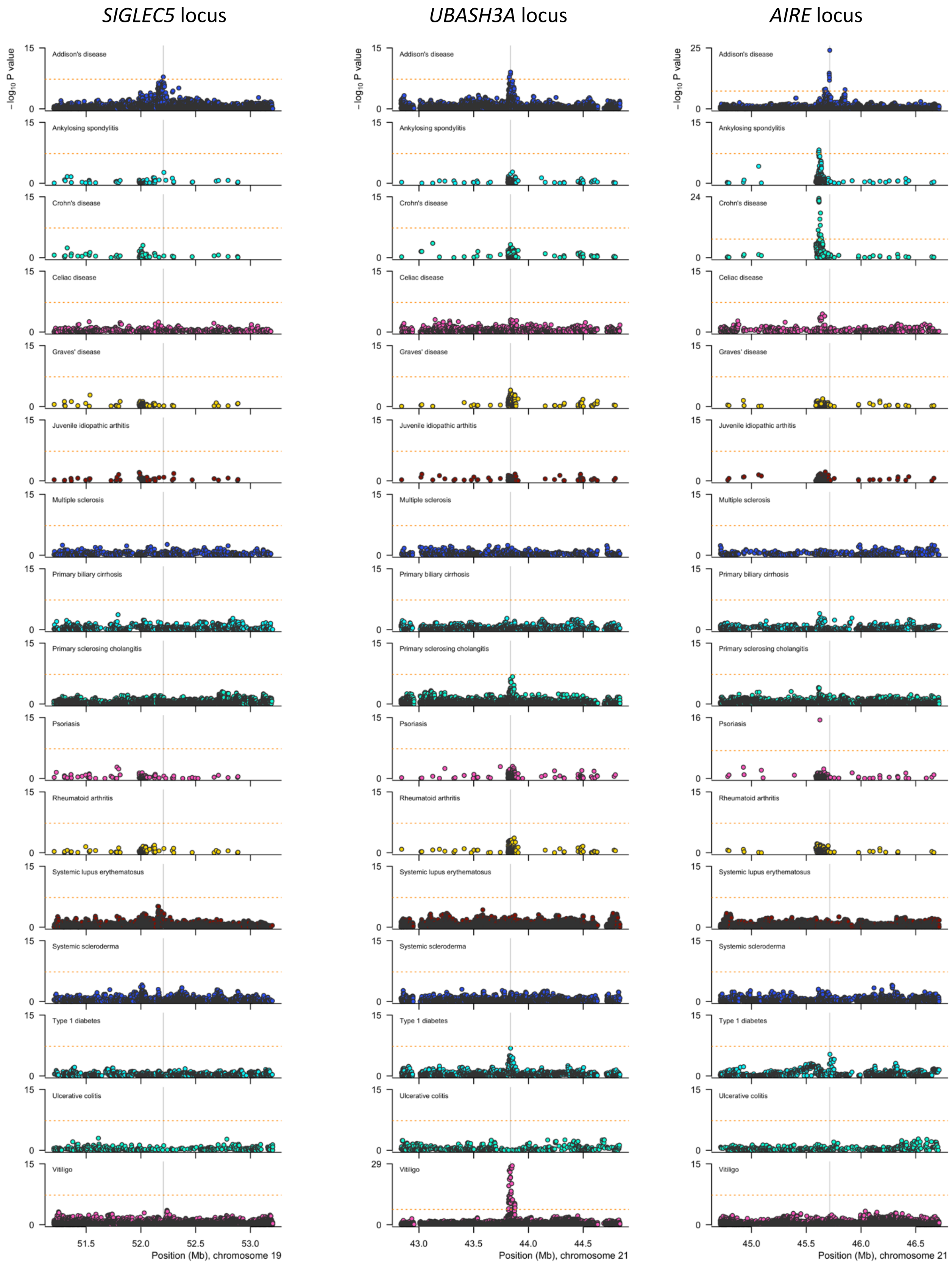
SIGLEC5 locus
UBASH3A locus
AIRE locus
−log10 P value
Addison's disease
Ankylosing spondylitis
Crohn's disease
Celiac disease
Graves' disease
Juvenile idiopathic arthritis
Multiple sclerosis
Primary biliary cirrhosis
Primary sclerosing cholangitis
Psoriasis
Rheumatoid arthritis
Systemic lupus erythematosus
Systemic scleroderma
Type 1 diabetes
Ulcerative colitis
Vitiligo
Position (Mb), chromosome 19
Position (Mb), chromosome 21
Position (Mb), chromosome 21

Supplementary Table 7, Loci previously implicated in AAD

Position is given on reference genome GRCh37. The odds ratio (OR) represents the effect of the minor allele (Min) on the odds of observing AAD, under an additive logistic regression model, such that an OR greater than one implies that the minor allele increases odds. Odds ratios and P values were estimated using logistic regression in 1,223 cases diagnosed with autoimmune Addison's disease and 4,097 controls.

**Chr: Position**	**Locus**	**SNP**	**Min**	**Alt**	**Cases**	**Controls**	**OR**	**P**
1: 114377568	PTPN22	rs2476601	A	G	0.17	0.11	1.74 (1.53-1.98)	6.3e-17
1: 157670816	FCRL3	rs7528684	A	G	0.57	0.56	1.04 (0.94-1.14)	4.5e-01
2: 191954852	STAT4	rs10931481	G	A	0.41	0.38	1.12 (1.02-1.24)	1.7e-02
2: 191958656	STAT4	rs4274624	C	T	0.28	0.25	1.19 (1.08-1.32)	7.2e-04
2: 204709349	CTLA4	rs231806	G	C	0.69	0.64	1.37 (1.22-1.52)	2.7e-08
2: 204732714	CTLA4	rs231775	G	A	0.51	0.45	1.28 (1.17-1.41)	1.0e-07
2: 204738888	CTLA4	rs34031880	C	T	0.036	0.032	1.31 (0.91-1.89)	1.5e-01
2: 204742934	CTLA4	rs11571302	G	T	0.71	0.64	1.28 (1.17-1.41)	6.2e-08
2: 204743409	CTLA4	rs7565213	G	A	0.71	0.64	1.29 (1.17-1.41)	6.0e-08
2: 204745003	CTLA4	rs11571297	T	C	0.68	0.61	1.28 (1.17-1.41)	6.2e-08
4: 123369776	IL2	rs3136534	G	T	0.40	0.37	1.11 (1.01-1.22)	3.2e-02
6: 90957463	BACH2	rs3757247	T	C	0.55	0.43	1.61 (1.47-1.77)	7.8e-24
6: 90976609	BACH2	rs62408233	A	G	0.44	0.32	1.69 (1.53-1.86)	1.3e-26
9: 5459419	PD-L1	rs1411262	C	T	0.78	0.74	1.2 (1.08-1.35)	1.2e-03
10: 8102272	GATA3	rs3802604	A	G	0.64	0.61	1.15 (1.05-1.26)	2.8e-03
10: 8108592	GATA3	rs569421	T	C	0.77	0.76	1.05 (0.94-1.17)	3.9e-01
12: 58157988	CYP27B1	rs4646536	A	G	0.68	0.67	1.03 (0.93-1.14)	5.8e-01
16: 11179873	CLEC16A	rs12708716	A	G	0.72	0.67	1.27 (1.15-1.41)	3.6e-06
16: 11189148	CLEC16A	rs12917716	G	C	0.64	0.59	1.25 (1.13-1.37)	9.8e-06
17: 5488352	NLRP1	rs925598	C	T	0.66	0.64	1.09 (0.99-1.2)	8.5e-02
18: 77245000	NFATC1	rs1667667	G	A	0.54	0.52	1.09 (0.97-1.23)	1.6e-01
18: 77246406	NFATC1	rs754093	T	G	0.56	0.55	1.06 (0.94-1.2)	3.3e-01
21: 45711808	AIRE	rs9983695	T	C	0.94	0.89	2.23 (1.8-2.75)	1.4e-13
X: 78426988	GPR174	rs3827440	T	C	0.53	0.58	1.04 (0.94-1.16)	4.2e-01

Supplementary Figure 8, Gene set enrichment analysis

Enrichment analysis for cell types, physiological systems, and tissues, using the DEPICT software [4]. Bars with false discovery rate less than or equal to 5% are highlighted in red. The peak in the musculoskeletal system represents synovia.

With a false discovery rate of 5%, the DEPICT software highlighted the immune system, lymphoid tissue, and in particular antigen-presenting cells as enriched for expression of our GWAS hits (P = 0.003).

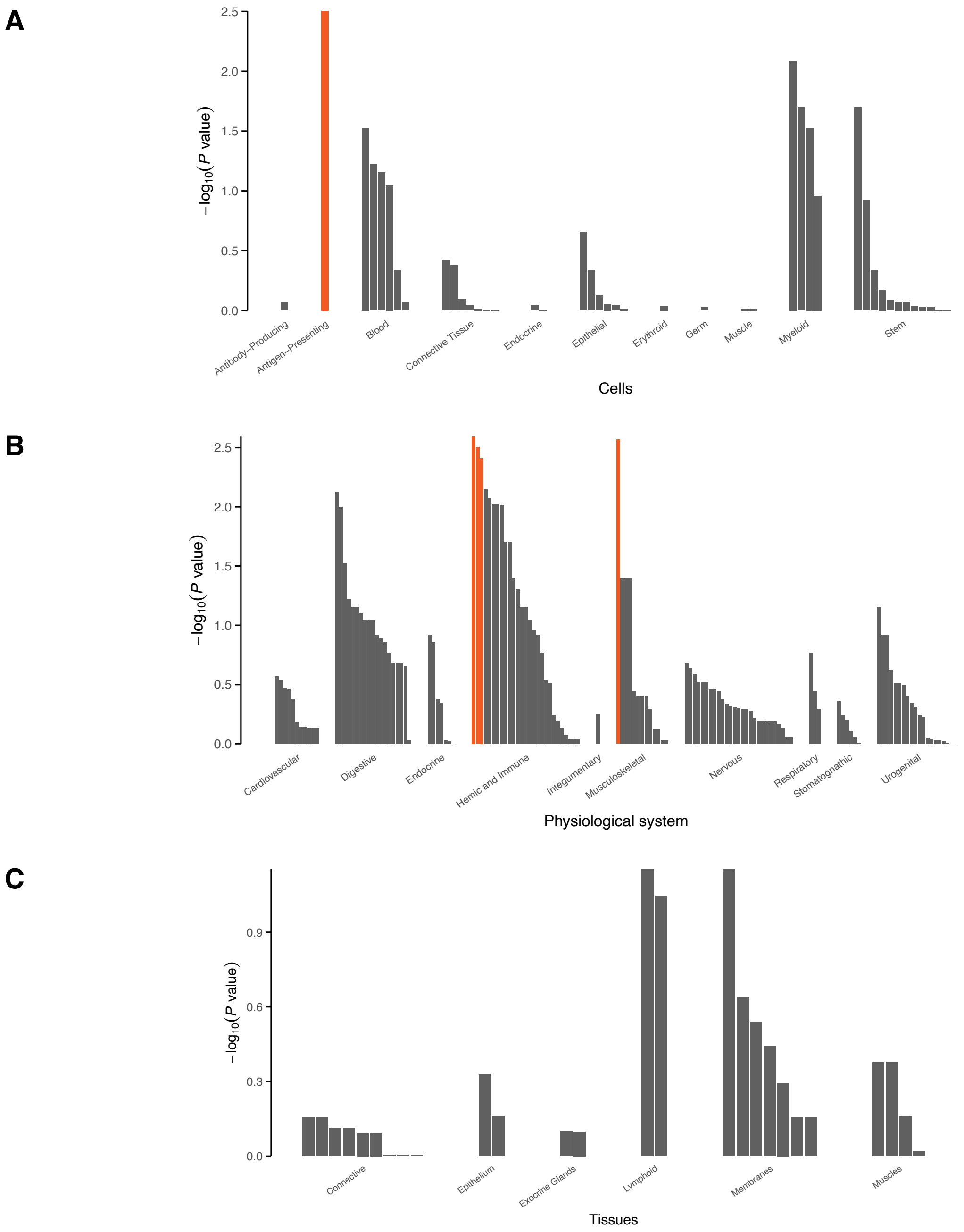

Supplementary Note 1, Data processing and quality control

Supplementary Figure 9, Overview of the raw data processing

Flowchart of sample and marker selection. Exclusion thresholds are detailed in the tables below.

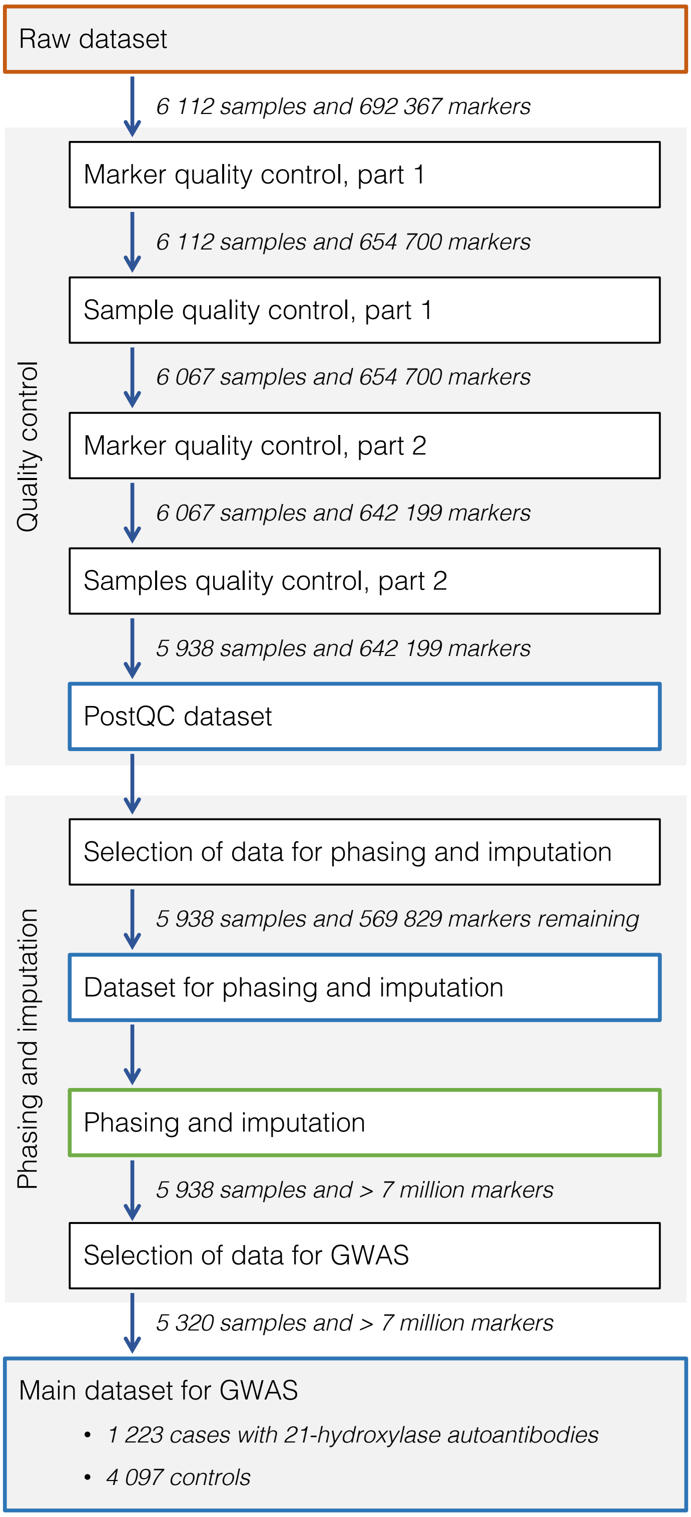

Supplementary Table 8, Raw dataset

■ Characteristics of the samples and markers before quality control.

**Samples**	6 112	
**Markers**	692 367 incl. technical<br>685 761 excl. technical	
**Nationality**	2 729 Norwegian	
	3 376 Swedish	
	7 European HapMap	
**Case-control status**	1 525 Cases	
	4 471 Controls	
	116 Neither case/control[1]	
**Sex**	3 153 Male	
	2 959 Female	
**Sex and case-control status**	2 520 male controls (42%)	588 male cases (10%)
	1 951 female controls (32%)	937 female cases (16%)

[1] Technical replicates, cases where Addison's disease was not autoimmune, and cases where other diagnosis was suspected after inclusion.

Supplementary Table 9, Marker quality control, part 1

List of criteria that were used to filter markers. The number of markers identified and removed are indicated for each criterium. 6 112 samples and 654 700 markers remained after marker quality control part 1

Filters	Data	Identified	Lost
**PAR3**	Markers on chromosome X, from position 88395830 to position 92583067 bp, were moved to the pseudoautosomal chromosome XY.	478	0
**Missing data > 5%**		3 532	3 532
**Duplicate SNPs**	Excluded the duplicate with more missing data	499	496
**AA theta mean > 0.3**		4 294	4 037
**AB theta mean > 0.8 or < 0.2**		523	498
**BB theta mean < 0.7**		3 511	3 346
**AA theta deviation > 0.06**		2 233	1 465
**AB theta deviation > 0.07**		5 602	4 945
**BB theta deviation > 0.06**		1 993	1 561
**Cluster separation < 0.35**		8 825	3 537
**10% Gene call score < 0.25**		11 929	5 644
**Blacklisted markers CHARGE**		172	114
**Blacklisted markers PCHIP**		2 143	1 886
**TOTAL LOST**		**31 061**	**31 061**

.

Supplementary Table 10, Sample quality control, part 1

List of criteria that were used to filter markers. 6 067 of the 6 112 samples remained after sample quality control part 1.

Filters	Data	Identified	Lost
**Missing data**	Chromosomes 1-22<br>Cut-off: 0.02	45	45
**Discordant sex**	Chromosome X, markers independent at r^2 0.5<br><br>Cut-off: <0.2 females, >0.8 males	0	0
**TOTAL LOST**		**45**	**45**

Supplementary Table 11, Identification of samples with sex chromosome disorders

**Filters**	**Data**	**Identified**
**Indeterminable sex**	Chromosome X, markers independent at r^2 0.5<br>Cut-off: >0.2 & <0.8	11
**Klinefelter**	Chromosome X and Y, markers independent at r^2 0.5	1
**Trisomy X**	Chromosome X and Y, markers independent at r^2 0.5	1
**XYY**	Chromosome Y. Male samples	0
**TOTAL IDENTIFIED**		13
**TOTAL LOST**		**0**

Supplementary Table 12, identification of global ancestry

Global ancestry was inferred with the LASER/TRACE software. Non-European samples were excluded from GWAS but kept for phasing and imputation due to appropriate genotyping quality.

**Filters**	**Data**	**Identified**
**Non-European ancestry**	Chromosomes 1-22, markers that overlap with HGDP	169
**TOTAL IDENTIFIED**		169
**TOTAL LOST**		**0**

Supplementary Table 13, Identification of samples from related subjects and identical samples

Identical samples represent a subset of related samples, including repeated registrations of the same subject, technical replicates of the same sample, and monozygotic twins. Identical and related samples were detected by investigating independent markers with low missingness and high allele frequency. Cases, males, and samples with higher call rates, were prioritized for inclusion.

Filters	Data	Identified
**Duplicated / related / replicated**	Autosomal markers with:<br>- missingness < 0.03<br>- MAF > 0.05<br>- independent at r^2 0.2<br>Cut-off: Pi-hat 0.1<br>Priority:<br>1) Case-Control pairs: controls excluded<br>2) Female-Male pairs: females excluded<br>3) Other pairs: low call rate excluded	309 identified for subsequent exclusion from GWAS
**TOTAL IDENTIFIED**		309
**TOTAL LOST**		**0**

Supplementary Table 14, Marker quality control, part 2

List of criteria that were used to filter markers. The number of markers identified and removed are indicated for each criterium. 642 199 markers remained after marker quality control part 2.

Filters	Data	Identified	Lost
**Missing data**	All samples. Call rate calculated in females and males, respectively, for chromosome X and Y.<br>Cut-off 0.02 for MAF ≥ 0.02. Cut-off 0.01 for MAF < 0.02.	6 490	6 490
**Discordant SNPs in identical samples**		1 621	1 380
**Discordant SNPs in HAPMAP controls**	7 duplicate HAPMAP samples compared to whole genome sequencing.	158	23
**HWE in autosomes**	Autosomal chromosomes of control subjects.<br>Left out: Non-European samples, and relatives.<br>Cut-off: 1e-6.	1 342	927
**HWE in X**	X-chromosome of control subjects.<br>Left out: Male samples, Non-European samples, samples with sex chromosome disorders, samples with indeterminable SNP-sex.<br>Cut-off: 1e-6.	2	2
**Markers with heterozygote males in chr X**	Male samples, chr X.<br>Left out: samples with sex chromosome disorders.	485	468
**Markers with heterozygote females in chr Y**	Female samples, chr Y.<br>Left out: samples with sex chromosome disorders.	565	564
**Markers with heterozygote males in chr Y**	Male samples, chr Y.<br>Left out: samples with sex chromosome disorders.	8	0
**Differential missingness cases/controls**	Autosomal chr.<br>Cut-off: 1e-5.	23	5
**Non-random missing haplotypes**	Autosomal chr.<br>Cut-off: 1e-8.	3 349	2 146
**Mendelian errors**			496
**TOTAL LOST**		**12 501**	**12 501**

Supplementary Table 15, Sample quality control, part 2

Samples with an abnormal level of heterozygosity as well as identical samples were removed. 5 938 samples remained after sample quality control part 2.

**Filters**	**Data**	**Identified**	**Lost**
**Heterozygosity and runs of homozygosity**	Chromosomes 1-22, MAF ≥ 0.05. Visual inspection. Cut-off: max 0.34 in European samples	6	6
**Identical samples**	Exclude one of each pair/series of identical samples: duplicate registrations, technical replicates and monozygotic twins. Keep the sample with the best call rate.	123	123
**TOTAL LOST**			**129**

Supplementary Table 16, Post QC dataset

- Characteristics of samples and markers that passed the quality controls.

**Samples**	5 938	
**Markers**	642 199	
**Nationality**	2 658 Norwegian	
	3 279 Swedish	
	1 European HapMap	
**Case-control status**	1 506 Cases	
	4 326 Controls	
	106 neither case/control	
**Nationality and case-control**	1 900 Norwegian control	696 Norwegian cases
	2 426 Swedish controls	810 Swedish cases
	103 neither case nor control	
**Sex**	3 060 Male	
	2 878 Female	
**Sex and case-control status**	2 434 male controls (41%)	581 male cases (10%)
	1 892 female controls (32%)	924 female cases (16%)

Supplementary Table 17, Selection of data for phasing

All samples remaining after quality controls were included in phasing. Multi-allelic sites, indels and ambiguous markers were removed prior to phasing.

Filters	Identified	Lost
SAMPLES	-	-
MARKERS		
**Multi-allelic sites.**	1 729	1 729
**Markers of insertions and deletions.**	22 401	22 063
**A/T and C/G SNPs with MAF > 0.4.**	409	406
**Markers not in the Human Reference Consortium dataset.**	59 865	48 172
**TOTAL LOST**		**72 370**

Supplementary Table 18, Data for phasing

- Samples and markers kept for phasing

**Samples**	5 938
**Markers**	569 829, of which 207 on chromosome Y

- **PHASING USING SHAPEIT v2.r837**

Supplementary Table 19, Imputation preparation

Preparation for imputation. 5 938 samples, and 567 222 markers remained after imputation preparation.

HRC-1000G-check-bim-v4.2.9 by Will Rayner

https://www.well.ox.ac.uk/~wrayner/tools/#Checking

POSITION MATCHING	**Autosomes**
**Total processed**	569 622
**ID matches HRC**	276 161
**Different position to HRC**	0
**Total position matches**	569 622
INDELS	
**Indels**	0
STRAND ALIGNMENT	
**SNPs to change ref alt**	78 230
**SNPs to change strand**	37 215
MISC	
**Palindromic SNPs with Freq > 0.4**	53
**Total removed for allele Frequency diff > 0.2**	1 566
**Non-matching alleles**	781
**ID and allele mismatching**	763; where HRC is . 553
**Duplicates removed**	0
**TOTAL LOST**	**2 400**

■ IMPUTATION AT SANGER IMPUTATION SERVICE: PBWT, HRC 1.1

Kept markers with INFO-score ≥ 0.5 and MAF ≥ 0.01.

Supplementary Table 20, Selection of data for GWAS

**Filters**	**Identified**	**Lost**
SAMPLES		
**Non-European samples**	158	158
**Samples from related subjects**	185	169
**Neither case nor control status in GWAS:**<br>**- Samples included from pedigrees**<br>**- Other cause of adrenal failure**	106	77
**HapMap control samples**	1	1
**TOTAL LOST**		**405**

Supplementary Table 21, Samples eligible for association studies

■Samples eligible for association studies.

**Samples**	5 533	
**Markers**	7.1 million	
**Nationality**	3 023 Swedish	2 510 Norwegian
**Case-control status**	4 097 Controls	1 436 Cases
**21-OH autoantibody status**	Positive: 1 223 cases<br>Negative: 211 cases<br>NA: 2 cases, 4 097 controls	

Supplementary Table 22, The GWAS dataset

■ 21-hydroxylase positive cases and all controls selected for the main GWAS analysis.

**Samples**	5 320	
**Markers**	7.1 million	
**Nationality**	2 899 Swedish	2 421 Norwegian
**Case-control status**	4 097 Controls	1 223 Cases
**Nationality and case-control**	1 852 Norwegian controls	569 Norwegian cases
	2 245 Swedish controls	654 Swedish cases
**Sex and case-control status**	2 300 male controls (41%)	464 male cases (10%)
	1 797 female controls (32%)	759 female cases (15%)
**Sex**	2 764 Men (52%)	2 556 Women (48%)

Supplementary Note 2, Functional assay of AIRE function

Plasmid amplification and mutagenesis

A custom pSF-UB plasmid containing *AIRE* wildtype was purchased from Oxford Genetics (Oxford Genetics Cat#: OG46). This plasmid was used for all *AIRE* Wt transfections and used as a base for creating the *AIRE* mutations p.R257X, p.S278R and p.R471C as described below. Plasmids were amplified using TOP10 competent *Escherichia coli* cells from Thermo Fisher and purified using a QIAprep Spin Miniprep Kit from Qiagen. Mutations were then created using the QuikChange II Site-Directed Mutagenesis kit from Agilent Technologies (Cat#: 200524-5). The p.R257X mutation was created using mutagenesis primers from Eurogentec with the forward sequence GAA-GCC-TCT-GGT-TTG-AGC-CAA-GGG-AG and the reverse sequence CTC-CCT-TGG-CTC-AAA-CCA-GAG-GCT-TC. While the p.S278R mutation was created using primers with the forward sequence CCA-GCA-GGG-CAG-AGT-TCC-CGC-CCC-TC and the reverse sequence CAG-GGG-CGG-GAA-CTC-TGC-CCT-GCT-GG. Similarly, the p.R471C mutation was created using primers with the forward sequence GGG-ACG-GGC-CTG-TGC-TGC-AGA-TCC-TG and the reverse sequence CAG-CAT-CTG-CAG-CAC-AGG-CCC-GTC-CC. The accuracy of *AIRE* and mutated *AIRE* containing plasmids were confirmed using Sanger sequencing with the Applied Biosystems 3730 DNA analyzer.

Cell culture and transfection

HEK293FT human embryonic kidney cells (RRID: CVCL_6911) were grown in a medium consisting of Dulbecco's Modified Eagle Medium (Cat#: 31966-021) supplemented with 4.5 g/l D-Glucose, Pyruvate, 10% (v/v) Fetal Bovine Serum (FBS), and 1% (v/v) Penicillin-Streptomycin. The cells were incubated at 37°C in a 5% CO2 humidified incubator until reaching 80-100% confluency. The cells were subsequently counted using a Scepter cell counter from Merck and transferred to 6-well plates, where each well was seeded with 3×10^5 cells. After 24h, the cells were transfected using the Lipofectamine 2000 Transfection Reagent from Thermo Fisher with 2.5µg DNA and 12µl Lipofectamine. Transfected cells were allowed to grow for 48 hours before being harvested. Two experiments were done, once with the *AIRE* wildtype, empty plasmid, and the *AIRE* mutants p.S278R and p.R471C, and once with *AIRE* wildtype, empty plasmid and the p.R257X mutant.

RNA isolation

Transfected cells were harvested by flushing, then transferred to 15ml tubes where they were centrifuged at 300g for 7 minutes before being resuspended in cold PBS. RNA was isolated using Qiagen RNeasy Mini Kit (Cat#: 74106) according to the RNeasy Mini Handbook. A Qiagen QIAshredder spin column (Cat#: 79656) was used to homogenize the samples, and a Qiagen RNase free DNase solution (Cat#: 79254) was used in order to digest any genomic DNA. Afterwards, the RNA samples were stored at -80°C. A NanoDrop microvolume spectrophotometer from Thermo Fisher was used to check the RNA concentration.

Real-time quantitative PCR

Previously isolated RNA was analyzed using qPCR in a two-step process. The first step consisted of using a Superscript VI VILO cDNA Synthesis Kit (Cat#: 11754050) to turn mRNA into cDNA according to the Product Info Sheet: SuperScript VILO cDNA Synthesis Kit. To analyze the cDNA a variety of TaqMan probes and a TaqMan Universal PCR Master Mix (Cat#: 4304437) were used according to the TaqMan Universal PCR Master Mix User Guide and subsequently analyzed using an Applied Biosystems 7900HT Fast Real-Time PCR System and the SDS 2.3 software. Three technical controls for each of three biological replicates were used, in addition to no template control (NTC) and no reverse transcriptase control (-RT). TaqMan probes were purchased from Thermo Fisher and consisted of probes for the genes *GAPDH* (Cat#: Hs02758991_g1), *AIRE* (Cat#: Hs00230829_m1), *CCNH* (Cat#: Hs00236923_m1), *KRT14* (Cat#: Hs00265033_m1), and *S100A8* (Cat#: Hs00374264_g1). The qPCR results were processed using the ΔΔCt method in Microsoft Excel 2016, and normalized against the *GAPDH* housekeeping gene, then fold change was calculated between the transfected cell populations and *AIRE* wildtype. Two-sided ANOVA (aov) was run using R (v. 3.5.3) [5] and RStudio (v. 1.1.456) [6] with the model ΔCt ~ group * gene, where 3 ΔCt values were used for each of 5 groups (Empty, AIRE wt, AIRE p.R257X, AIRE p.S278R and AIRE p.R471C), and 95% confidence intervals was calculated from SE of each group. A Tukey multiple

comparisons of means (TukeyHSD) test was then run on the means of the ANOVA. Data was prepared in R using the tidyverse (v. 1.2.1) [7] package, then plotted using ggplot2 (v. 3.2.0) [8] with the additional packages RColorBrewer (v. 1.1-2) [9] and scales (v. 1.0.0) [10].

Supplementary Note 3, Building a model of HLA risk for autoimmune Addison's disease

Stepwise variable selection

For every HLA allele and amino acid residue in turn (variables), we built several logistic regression models. Bayesian Information Criterion (BIC) was used to decide whether the effect was best described as additive, recessive, dominant, overdominant or general. The variable from the most significant model was considered for inclusion in the full model. Nested models with single and multiple terms describing the effect of the selected variables were compared with likelihood ratio tests (LRT). Five PCs and sex were included as covariates in all models. PCs were calculated from SNPs genome-wide, not including the HLA region, i.e. the same covariates as in the GWAS analysis. We proceeded stepwise, including the allele with most compelling evidence for association at every step. Backward elimination was performed by leaving previous variables out of the current model, one by one, and by subsequently testing the goodness of fit. We only included variables that met genome-wide significance $P < 5 \times 10^{-8}$ at inclusion, and in the final model.

We started by testing every HLA allele in turn, together with PC and sex covariates:

$$Model_1 = b_0 + G \times b_1 + PC_1 \times b_{PC1} + PC_2 \times b_{PC2} + PC_3 \times b_{PC3} + PC_4 \times b_{PC4} + PC_5 \times b_{PC5} + Sex \times b_{Sex}$$

and compared models where the effect of an individual's genotype G was encoded as additive, dominant, recessive, and overdominant, respectively. The most significant effect for the variable was brought forward. We subsequently used LRT to compare models with one effect to models with two effects for the same variable:

$$Model_2 = b_0 + G \times b_1 + H \times b_H + PC_1 \times b_{PC1} + PC_2 \times b_{PC2} + PC_3 \times b_{PC3} + PC_4 \times b_{PC4} + PC_5 \times b_{PC5} + Sex \times b_{Sex}$$

where H denotes a second effect for the same allele, encoded as additive, dominant, recessive, or overdominant, depending on the encoding of the primary effect. In practice, only HLA-DQB1*02:01 was described significantly better with two terms, representing deviation from a pure additive effect.

DQB1*02:01. The strongest signal was seen at DQB1*02:01 with P values 10^{-160} and 10^{-149} for the dominant and additive encoding, respectively. DQB1*02:01 has been associated with AAD in several previous studies. We found that DQB1*02:01 was in strong LD ($r^2 > 0.95$) with DQA1*05:01 and DRB1*03:01, i.e. the known risk haplotype. Including a homozygote correction term significantly improved the fit of the additive model ($P_{LRT} = 1.5 \times 10^{-9}$), and a negative b_H indicated a partially dominant effect of DQB1*02:01. In other words, the risk effect of DQB1*02:01 in homozygotes was significantly less than double the effect in heterozygotes. Since both the additive and homozygote correction terms withstood backward elimination and remained significant in the full model, we decided to keep both terms for DQB1*02:01 in the model.

DQB1*03:02. In a 2-variable model, and across all HLA alleles and amino acids, DQB1*03:02 was the most significant variable with $P = 10^{-81}$. The additive effect gave the best fit, and no second term with alternative encoding could improve the model. Since we found no support for deviation from additivity, we assumed an additive effect and included DQB1*03:02 in the model. We note that DQB1*03:02 is in high LD with DQA1*03:01 and DRB1*04:04, and the result is therefore consistent with previous studies that have associated this haplotype with risk of AAD.

DQB1 pos. 30 Tyrosine. For all 3-variable models, including the amino acid residue DQB1 pos. 30 Tyr gave the strongest signal, $P = 10^{-35}$. The additive effect was the strongest, and a homozygote correction term was significant at $P = 3 \times 10^{-4}$. Hence, a significant deviation from additivity was noted and was included as a homozygote correction term when DQB1 pos. 30 Tyr was added to the model. With the inclusion of DQB1 pos. 30 Tyr in the model, the homozygote correction term from step 1, for DQB1*02:01 dropped in significance to P = 0.07, and was excluded in backward elimination.

DQB1 pos. 30 Tyr unite HLA alleles DQB1*03, DQB1*04, DQB1*05:04, DQB1*06:01, DQB1*06:02, and DQB1*06:09.

B pos. 74 Aspartic acid. Across 4-variable models, B pos. 74 Asp showed the strongest association with AAD, $P = 10^{-23}$. We noted no deviation from additive effect and a single term was included in the model to account for the effect from B pos. 74 Asp. An aspartic acid residue in position 74 unites HLA alleles B*08:01 and B*07:02.

DRB1*04:04. In a 5-variable model, a second HLA class II locus was the most significant, $P = 10^{-14}$. DRB1*04:04, with no deviation from additive effects was included in the model. It is worth noting, however, that DRB1*04:04 is in moderate LD with one of the major risk haplotypes, DQA1*03:01 ($r^2 = 0.43$), DQB1*03:02 ($r^2 = 0.43$). Still, inclusion of DRB1*04:04 made a significant improvement of the model and underlines the importance of HLA class II in AAD risk.

B pos. 156 Aspartic acid. Testing all 6-variable models, we found that B pos. 156 Asp showed the strongest association with AAD, $P = 10^{-11}$. Since no deviation from additivity could be detected, the effect was assumed to be additive and B pos. 156 Asp was included in the model. B pos. 156 Asp unite B*08:01 ($r^2 > 0.5$) and B*37:01.

DQA1*01:04. When looking across the other HLA variables for remaining association with AAD, DQA1*01:04 stood out as the most significant, $P = 1.4 \times 10^{-8}$. No departure from additive effects was significant and the effect of DQA1*01:04 was assumed to be additive when included in the model. DQA1*01:04 was in strong LD with DQB1*05:03 ($r^2 > 0.95$) and DRB1*14:01 ($r^2 > 0.95$), neither of which had previously been included in the model, and together represent a rare haplotype. We note, however, that the statistical significance was close to the genome-wide threshold and that the allele frequency was low. Naturally, inclusion of further variables did not improve the model and DQA1*01:04 was the last variable to be included before interaction analysis.

Interactions among classical HLA alleles

We next investigated the presence of interactions among HLA alleles and amino acids included in the model and all other HLA alleles and amino acids (A) in the dataset. For all interaction models,

$$\begin{aligned} Model_{Int-DQB1*02:01} &= b_0 + G_{DQB1*02:01} \times b_1 + H_{DQB1*02:01} \times b_H + G_{DQB1*03:02} \times b_2 \\ &+ G_{DQB1\ pos\ 30\ Tyr} \times b_3 + G_{B\ pos\ 74\ Asp} \times b_4 + G_{DRB1*04:04} \times b_5 + G_{B\ pos\ 156\ Asp} \times b_6 \\ &+ G_{DQA1*01:04} \times b_7 + G_A \times b_{10} + I_{DQB1*02:01} \times G_A \times b_{11} + PC_1 \times b_{PC1} + PC_2 \times b_{PC2} + PC_3 \times b_{PC3} \\ &+ PC_4 \times b_{PC4} + PC_5 \times b_{PC5} + Sex \times b_{Sex} \end{aligned}$$

I_x indicated the presence of variable x in an individual's genotype G_x.

Interaction with DQB1*02:01

Only three variables showed significant interactions at the genome-wide level (dashed red line in QQ-plots, Supplementary Fig. 10); alleles DQB1*02:01, DQB1*03:02, and DRB1*04:04. Interactions of DRB1*04:04 with either DQB1*02:01 or DRB1*03:01 were equally significant. The LD between DQB1*02:01 and DRB1*03:01 was strong ($r^2 > 0.95$) and the interaction signals presumably derive from the same underlying effect. We selected to further analyze the interaction with DQB1*02:01, since DQB1*02:01 was already included in the model.

Inclusion of the interaction term for DQB1*02:01 and DRB1*04:04 significantly improved the fit of the model $P_{LRT} = 5 \times 10^{-11}$, but decreased the significance of the homozygote correction term for DQB1 position 30.Y from 3×10^{-8} to 6×10^{-4}. According to BIC, the model was better without the homozygote correction term at this point, and in backward elimination, the homozygote correction term was excluded.

The interaction term for DQB1*02:01 and DRB1*04:04 also affected the significance of the additive effect for DRB1*04:04, that decreased from 1×10^{-15} to 5×10^{-4}. Logically, the additive term of DRB1*04:04 was also removed in backward elimination. According to BIC, the overall fit of the model improved with the inclusion of the interaction

term, despite the exclusion of both the homozygote correction term for DQB1 position 30.Y and the additive effect of DRB1*04:04. We therefore excluded the additive effect of DRB1*04:04 from the model.

From this point, additional steps of regression could not detect additional alleles that significantly improved the model at the predefined level of significance. We note that the interaction between DQB1*03:02 and DRB1*04:04, that was promising in the first round of interaction modeling, lost its significance after inclusion of the interaction term for DQB1*02:01 and DRB1*04:04, presumably due to the LD between DQB1*03:02 and DRB1*04:04 (r^2 = 0.36). Hence, the final model had the following definition:

$$\begin{aligned} Model \quad = {} & b_0 + G_{DQB1*02:01} \times b_1 + G_{DQB1*03:02} \times b_2 \\ & + G_{DQB1\,pos\,30\,Tyr} \times b_3 + G_{B\,pos\,74\,Asp} \times b_4 + I_{DQB1*02:01} \times G_{DRB1*04:04} \times b_5 + G_{B\,pos\,156\,Asp} \times b_6 \\ & + G_{DQA1*01:04} \times b_7 + PC_1 \times b_{PC1} + PC_2 \times b_{PC2} + PC_3 \times b_{PC3} + PC_4 \times b_{PC4} + PC_5 \times b_{PC5} \\ & + Sex \times b_{Sex} \end{aligned}$$

Supplementary Figure 10, Q-Q plots for interaction term P values

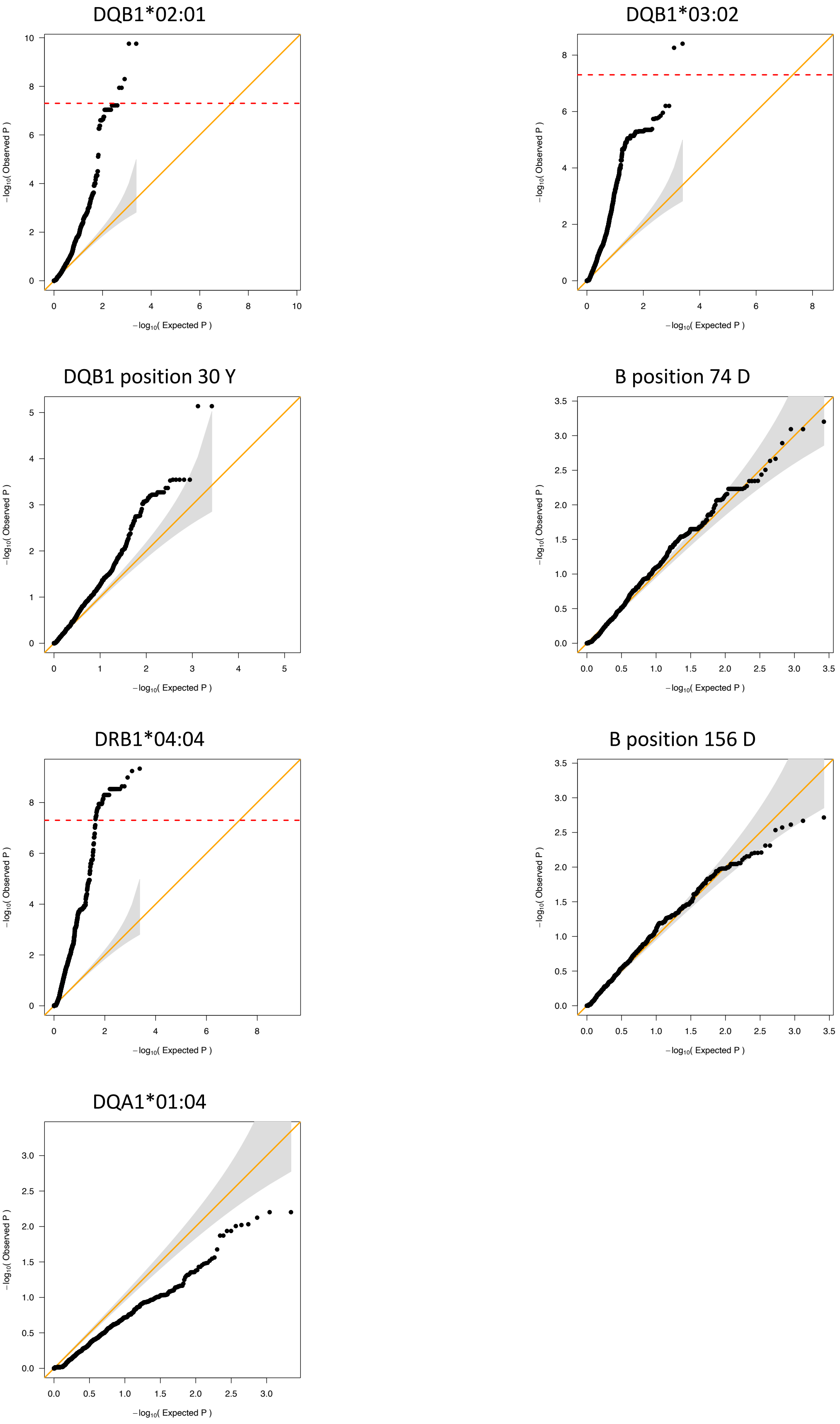

Supplementary References

1 Pruim, R. J. *et al.* LocusZoom: regional visualization of genome-wide association scan results. *Bioinformatics (Oxford, England)* **26**, 2336-2337, doi:10.1093/bioinformatics/btq419 (2010).

2 Staley, J. R. *et al.* PhenoScanner: a database of human genotype-phenotype associations. *Bioinformatics (Oxford, England)* **32**, 3207-3209, doi:10.1093/bioinformatics/btw373 (2016).

3 Kamat, M. A. *et al.* PhenoScanner V2: an expanded tool for searching human genotype-phenotype associations. *Bioinformatics (Oxford, England)* **35**, 4851-4853, doi:10.1093/bioinformatics/btz469 (2019).

4 Pers, T. H. *et al.* Biological interpretation of genome-wide association studies using predicted gene functions. *Nature communications* **6**, 5890, doi:10.1038/ncomms6890 (2015).

5 R: A Language and Environment for Statistical Computing (R Foundation for Statistical Computing, Vienna, Austria, 2019).

6 RStudio: Integrated Development Environment for R (RStudio, Inc., Boston, MA, 2015).

7 Wickham et al. Welcome to the tidyverse. *Journal of Open Source Software* **4**, 1686, doi:10.21105/joss.01686 (2019).

8 Wickham, H. Ggplot2 : elegant graphics for data analysis. (2009).

9 RColorBrewer: ColorBrewer Palettes v. 1.1-2 (2014).

10 scales: Scale Functions for Visualization v. 1.1.0 (2019).
