# Supplementary material for: GWAS for autoimmune Addison’s disease identifies multiple risk loci and highlights AIRE in disease susceptibility
Source: Nat Commun. 2021 Feb 11;12:959. doi: 10.1038/s41467-021-21015-8 (PMC7878795; doi:10.1038/s41467-021-21015-8)
Supplement: Supplementary file 3 — Reporting Summary [file 41467_2021_21015_MOESM3_ESM.pdf]

nature research

Corresponding author(s): Eystein Sverre Husebye

Last updated by author(s): 2020-11-XX

Reporting Summary

Nature Research wishes to improve the reproducibility of the work that we publish. This form provides structure for consistency and transparency in reporting. For further information on Nature Research policies, see our Editorial Policies and the Editorial Policy Checklist.

Statistics

For all statistical analyses, confirm that the following items are present in the figure legend, table legend, main text, or Methods section.

n/a	Confirmed	
☐	☒	The exact sample size (*n*) for each experimental group/condition, given as a discrete number and unit of measurement
☐	☒	A statement on whether measurements were taken from distinct samples or whether the same sample was measured repeatedly
☐	☒	The statistical test(s) used AND whether they are one- or two-sided<br>*Only common tests should be described solely by name; describe more complex techniques in the Methods section.*
☐	☒	A description of all covariates tested
☐	☒	A description of any assumptions or corrections, such as tests of normality and adjustment for multiple comparisons
☐	☒	A full description of the statistical parameters including central tendency (e.g. means) or other basic estimates (e.g. regression coefficient) AND variation (e.g. standard deviation) or associated estimates of uncertainty (e.g. confidence intervals)
☐	☒	For null hypothesis testing, the test statistic (e.g. *F*, *t*, *r*) with confidence intervals, effect sizes, degrees of freedom and *P* value noted<br>*Give P values as exact values whenever suitable.*
☐	☒	For Bayesian analysis, information on the choice of priors and Markov chain Monte Carlo settings
☐	☒	For hierarchical and complex designs, identification of the appropriate level for tests and full reporting of outcomes
☐	☒	Estimates of effect sizes (e.g. Cohen's *d*, Pearson's *r*), indicating how they were calculated

Our web collection on statistics for biologists contains articles on many of the points above.

Software and code

Policy information about availability of computer code

Data collection	No software was used for data collection.
Data analysis	Genome Studio 2.0.4, Plink 1.9, bcftools 1.8, Locus zoom 0.10.0, PhenoScanner 2, SHAPEIT 2.r837, perl 5.26.2, LASER 2.04, DEPICT 1.r173, LDHub 1.0.0, Sanger Imputation Server (PBWT) w. HRC r1.1, SNP2HLA 1.0.3, FINEMAP 1.4, PyMOL, Circos 0.69-6, SDS 2.3, Microsoft Excel 2016, R 3.5.0, RStudio 1.1.456, R packages: tidyverse 1.2.1, ggplot2 3.0.0 and 3.2.0, RColorBrewer 1.1-2, scales 0.5.0 and 1.0.0., GWASTools 1.26.1, data.table 1.11.4, openxlsx 4.1.0, gridExtra 2.3, reshape2 1.4.3, dplyr 0.7.6, grid 3.5.0, HIBAG 1.16.0, parallel 3.5.0, tidyr 0.8.1, plyr 1.8.4, stringr 1.3.1, R.utils 2.6.0, gplots 3.0.1

For manuscripts utilizing custom algorithms or software that are central to the research but not yet described in published literature, software must be made available to editors and reviewers. We strongly encourage code deposition in a community repository (e.g. GitHub). See the Nature Research guidelines for submitting code & software for further information.

Data

Policy information about availability of data

All manuscripts must include a data availability statement. This statement should provide the following information, where applicable:

- Accession codes, unique identifiers, or web links for publicly available datasets
- A list of figures that have associated raw data
- A description of any restrictions on data availability

Summary statistics can be accessed at www.ebi.ac.uk/gwas/, accession code GCST90011871. No figures include raw data. Genotype data is not publicly available due to ethics restrictions.

Field-specific reporting

Please select the one below that is the best fit for your research. If you are not sure, read the appropriate sections before making your selection.

- [x] Life sciences
- [ ] Behavioural & social sciences
- [ ] Ecological, evolutionary & environmental sciences

For a reference copy of the document with all sections, see nature.com/documents/nr-reporting-summary-flat.pdf

Life sciences study design

All studies must disclose on these points even when the disclosure is negative.

Sample size	The Swedish and Norwegian Addison Registries are by far the world's two largest collections of samples and clinical information from patients with primary adrenal failure. By including all available patient samples and five times as many controls, we have made the study as large as possible.
Data exclusions	Samples were excluded from further analyses if they met any of the following criteria: Genotype call rates < 98%, accumulated heterozygosity > 0.34, were closely related to other study samples (pi-hat > 0.1), were of non-European ancestry, had non-autoimmune Addison's disease or a known monogenic cause of autoimmune Addison's disease. All exclusion criteria were pre-established.
Replication	Given the rarity of the disease, replication of the GWAS in an independent cohort was not possible, but the main findings were consistent when the two subsets (Norwegian and Swedish groups) were analyzed separately. For the expression analyses, two experiments (each with three biological replicates) were performed, and their results exhibited the same trend .
Randomization	Not applicable: Case-control design.
Blinding	Not applicable: Case-control design.

Reporting for specific materials, systems and methods

We require information from authors about some types of materials, experimental systems and methods used in many studies. Here, indicate whether each material, system or method listed is relevant to your study. If you are not sure if a list item applies to your research, read the appropriate section before selecting a response.

Materials & experimental systems

n/a	Involved in the study	
x		Antibodies
	x	Eukaryotic cell lines
x		Palaeontology and archaeology
x		Animals and other organisms
	x	Human research participants
x		Clinical data
x		Dual use research of concern

Methods

n/a	Involved in the study	
x		ChIP-seq
x		Flow cytometry
x		MRI-based neuroimaging

Eukaryotic cell lines

Policy information about cell lines

Cell line source(s)	HEK293FT cells (Thermo Fisher, May 2015)
Authentication	The cell line used has not been authenticated.
Mycoplasma contamination	The cell line used tested negative for mycoplasma contamination.
Commonly misidentified lines (See ICLAC register)	No commonly misidentified cell lines were used in this study.

Human research participants

Policy information about studies involving human research participants

Population characteristics	All participants were over 18 years of age (age distribution otherwise unknown for controls), 48% females and 52% males, all cases were diagnosed as having Addison's disease and as such were receiving life-long hormone replacement therapy. Controls had no disclosed chronic disease. Sex and PCs (to account for residual population sub-structuring) were included as covariates in the analysis.
Recruitment	Cases were recruited from unbiased national registries of Addison's patients (ca. 65% of all diagnosed Norwegian patients, ca. 75% of all diagnosed Swedish patients), and the majority of registry patients consented to participate, and were therefore included, in the study. As controls were recruited on-site at blood donor centres, self-selection bias should be minimal (barring any that might prompt initial donation). We consider it unlikely that there is any recruitment bias that would materially affect our results.
Ethics oversight	The study was performed in accordance with the Declaration of Helsinki and approved by the Swedish Ethical Review Authority - Stockholm (dnr 2008/296-31/2), and the Norwegian Regional Committee for Medical and Health Research Ethics - West (biobank 2013-1504, project 2017-624).

Note that full information on the approval of the study protocol must also be provided in the manuscript.
