# Supplementary material for: GWAS for autoimmune Addison’s disease identifies multiple risk loci and highlights AIRE in disease susceptibility
Source: Nat Commun. 2021 Feb 11;12:959. doi: 10.1038/s41467-021-21015-8 (PMC7878795; doi:10.1038/s41467-021-21015-8)
Supplement: Supplementary file 4 — Description of Additional Supplementary Files [file 41467_2021_21015_MOESM4_ESM.pdf]

Description of Additional Supplementary Files

Supplementary Data 1
Fine-mapping
Credible configurations and sets from FINEMAP. k-max - the maximum number of causal loci permitted in the 1Mb window around the lead GWAS SNP; Locus_code - refers to a GWAS peak, one per chromosome, except for chromosome 21; Set - identifier for credible configurations, na for credible sets (where k-max=1); rsID - SNP ID where it exists; chr. – chromosome; pos. - hg19 chromosomal position; prob. - probability from FINEMAP; log10BF - log10 Bayes Factor from FINEMAP; Var_annot - variant type; Gene - gene in which the variant lies, if any; DICE_eQTL_gene - gene(s) whose regulation is correlated with given SNP's genotype state in the DICE database; DICE_eQTL_cell_type - the cell types in which the above gene is statistically significantly differentially expressed; T_all - all T cells, T_var - a subset of various T cells, T_helper_var - a subset of various helper T cells, naive_T - naive T cells (all assayed types); GTEx_gene - gene(s) whose regulation is correlated with given SNP's genotype state in the GTEx database;

GTEx_combined_eQTL - the tissue/cell types in which the above gene is statistically significantly differentially expressed, where more than two, given as var_tissues, WB - whole blood; Encode-cCREs - candidate cis-regulatory elements from ENCODE; H3K27Ac_7cellENCODE - general activating histone mark, from 7 cell lines (ENCODE); Txn_Factor_ChIP - UCSC track Transcription Factor ChIP-seq Clusters (161 factors) from ENCODE with Factorbook Motifs; only presence or absence given, but note that most identified signals were weak.
