# Supplementary material for: GWAS for autoimmune Addison’s disease identifies multiple risk loci and highlights AIRE in disease susceptibility
Source: Nat Commun. 2021 Feb 11;12:959. doi: 10.1038/s41467-021-21015-8 (PMC7878795; doi:10.1038/s41467-021-21015-8)
Supplement: Supplementary file 2 — Peer Review File [file 41467_2021_21015_MOESM2_ESM.pdf]

Reviewer #1 (Remarks to the Author):

This is a really well written and performed GWAS of Addison's Disease (AD). The study looks at just a subset of AD patients, excluding patients with APS1 (aka APACED), but leaving in patients with APS2 who make up ~2/3 of the cases. Key findings are common variant association with two variants of AIRE with AD, mutations of which cause APS1, and associations in 8 other genes, all known to be associated with a wide range of immune-mediated disorders, including particularly organ-specific autoimmune diseases. No difference in associations is seen with isolated AD and APS2.
The study is very neat and I have only a few comments/suggestions.
1. The HLA analysis is simplistic and could be improved. In some diseases imputation down to amino-acid level has proven able to identify key sequences associated with disease even on long haplotypes (e.g. (1, 2)). Including such an analysis would strengthen the study. The authors have performed a logistic regression analysis of HLA alleles themselves, but it's not clear to me from the description how many different association signals the authors claim at the MHC, and what their boundaries are. Would be helpful to clarify that in the paper. In Figure 3 it would be helpful to list the conditional steps taken between each figure.
2. Whilst I think AIRE is the likely candidate gene, particularly for the coding variant rs74203920, it lies next to other strong candidate genes including ICOSLG and DNMT3L, which show association with other diseases (e.g. see (3), showing association of this locus with AS, CD, UC and PSC, but implicating DNTM3L). The functional analysis presented in Supplementary Figure 4 does not show an effect of either rs74203920 or rs1800520 on AIRE dependent gene expression, something the authors ascribe to the variants been polymorphisms. Given the substantial odds ratio that rs74203920 in particular carries, the lack of an effect in this assay suggests other explanations, such as that the cell type studied is not a relevant cell type for AIRE (HEK293 cells, renal origin, used), or that other nearby genes may be involved (though this seems intuitively less likely). These alternate options should be mentioned and discussed in the manuscript.
3. This may be for a subsequent paper, but the effect sizes reported here for AIRE and HLA are large. Diagnosis of AD is often delayed, sometimes with fatal consequences. It would be nice to see an analysis of the utility of a genetic risk score for AD.
1. Leo PJ, Madeleine MM, Wang S, Schwartz SM, Newell F, Pettersson-Kymmer U, et al. Defining the genetic susceptibility to cervical neoplasia-A genome-wide association study. PLoS Genet. 2017;13(8):e1006866.
2. Han B, Diogo D, Eyre S, Kallberg H, Zhernakova A, Bowes J, et al. Fine mapping seronegative and seropositive rheumatoid arthritis to shared and distinct HLA alleles by adjusting for the effects of heterogeneity. Am J Hum Genet. 2014;94(4):522-32.
3. Ellinghaus D, Jostins L, Spain SL, Cortes A, Bethune J, Han B, et al. Analysis of five chronic inflammatory diseases identifies 27 new associations and highlights disease-specific patterns at shared loci. Nat Genet. 2016;48(5):510-8.

Reviewer #2 (Remarks to the Author):

This is an exceptionally interesting manuscript, describing the results of the first GWAS of Addison's disease (AAD), a rare but historically important autoimmune (AI) disease that constitutes the most common cause of primary adrenal failure. Given the rarity of AAD, this GWAS is of reasonable size to provide decent power, the work was very well performed (the scrupulous case selection is particularly impressive), the analyses seem sound (though a bit limited), the interpretations are generally reasonable, and the writing is excellent. The man findings are of genetic association of AAD with a number of genes that largely have been previously found associated with other AI diseases, many of which are epidemiologically associated with AAD. The only truly novel finding is of polygenic association with two coding variants in AIRE, mutations in which are causal for autosomal recessive APECED, a multiple AI disease that primarily includes AAD, and which have not previously been

associated with risk of other autoimmune diseases. The findings regarding AIRE constitute the main novel result, seem of considerable interest, but deserve much deeper analyses than are reported here.

Specific suggestions:

1. It seems to me the title is overstated. Given the findings of GWAS of other AI diseases, I think it can safely be said that many (if not most or all) AI diseases, including AAD, involve components of loss of both peripheral and central tolerance. The only real difference is the involvement of AIRE in AAD, which suggests a perhaps greater role for loss of central tolerance in AAD than in other AI diseases. Nevertheless, I do believe the authors should change the title to one more descriptive of their findings.
2. Regarding the reported associations with coding variants in AIRE, the authors describe rs74203920-T as "relatively rare in our Swedish and Norwegian controls (2.0%) and in thenon-Finnish European population (1.4% GnomAD v2.1.1)". However, that MAF is, by definition, not "rare". Rather than saying "relatively rare" I would suggest "uncommon". Unless I missed it somewhere, given that APECED is recessive, there should be a formal analysis of association of AAD with the two AIRE variants singly and together under both polygenic and recessive models, and a corresponding table of the results presented at least in the supplementary material. Furthermore, given that AIRE is on chr21, a sentence or two of speculation in the Discussion on the possible role of AIRE in the very high prevalence of AI diseases in patients with Down Syndrome would not be unwarranted.
3. The section, "Sub-significant loci…." reports nothing of interest and should be deleted in its entirety. That will make room for other analyses that would be of greater interest.
4. Similarly, the section, "Gene set enrichment analysis…" also reports nothing novel, which is not surprising as all of the loci that are discussed are well known in other AI diseases. This section should be reduced to literally a single sentence or two in the discussion.
5. Nevertheless, Fig. 4 and Supplementary Fig. 5 are of considerable interest; I believe that analysis should be expanded in the present report. It has long been noted that occurrence of other AI diseases is much more common in AAD cases than in other AI diseases; is evident even in Addison's original case study. Accordingly, a Supplementary Table reporting the nature and prevalence of other AI diseases in the cases used in this GWAS is absolutely required here. Second, it has been speculated that the rarity of AAD coupled with the exceptionally high epidemological association with other AI diseases suggests that these diseases are all genetically related, but that might be biologically "harder" to get AAD than these other diseases (in terms of a burden threshold). As twin heritability is close to 1, that suggests the effect is almost completely genetically determined. Might the authors be able to carry out an analysis of the genetic burden conferred by the non-AIRE AAD-associated alleles in AAD versus the other AI diseases to which these loci contribute? Alternatively, might it be that the AIRE variants associated here are essentially necessary but not sufficient for AAD? Could the authors partition their AAD cases into subsets with versus without the associated AIRE variants and test for differential association with the other loci they report? These types of questions would seen to warrant analysis and discussion, but are generally absent from the manuscript.
6. Along the same lines, the authors report a GCTA analysis using the GWAS data, but it is difficult to understand what they report. They state that previous studies report twin heritability for AAD of 97%. From the GCTA analysis and AAD prevalence studies, they report "a SNP heritability rate for AAD between 34% and 40%". I am not sure what they mean by "SNP heritability rate". Do they mean h2? Obviously, the key questions are what fraction of twin heritability is captured by h2 as measured by GCTA, which here included only common SNPs with MAF >/ 1% (the meaning of the last sentence of the abstract is especially unclear in this regard, and should be rewritten); what fraction of that is accounted for by the associated loci they report based on the GWAS results; and what fraction of that is accounted for by the two AIRE variants, which are novel to this study.

Reviewer #3 (Remarks to the Author):

The authors of this manuscript describe the first GWAS of AAD. While several studies have explored

candidate loci usually that had been associated with other autoimmune diseases prior to this work, only a few loci had surpassed genome-wide significance. While they were able to replicate these loci, they also found 4 novel associations. The authors go on to explore the signal near AIRE identifying a second independent signal for what had been previously reported. The HLA is the strongest association with AAD, and after classical allele imputation and stepwise regression, HLA DQB1*02:01 and DQB1*03:02 were the top signals in the region. The authors then show how the associated loci relate to other autoimmune diseases. This work is a very important step towards understanding the genetic determinants of AAD; however, further evaluation of the loci should be done in the loci outside of those previously implicated prior to this study. While the work in the HLA and AIRE are important, very little was done in the other regions to further our understanding of how they might contribute to AAD pathophysiology, particularly the 4 novel loci. In addition, here are some specific points:

1)The authors should use Bayesian statistics to identify creditable sets of variants for all loci identified in this study. Currently, the authors have only discussed conditional analysis on AIRE and stepwise with the HLA.

2)The authors should use this information from the creditable sets to evaluate each region for eQTLs, chromatin-chromatin iterations, evidence of altering protein biding motifs and/or ChIP-seq peaks, histone marks, etc. from bioinformatics dataset that widely available for immune cell populations. This can then help shorten the credible set further.

3)The plot of the PCA should be shown in the supplement. What was the rationale for using 5 PCs as covariates in the regression model?

4)The authors do not indicate which variants were imputed and which were genotyped. Was there association signals in each genome-wide significant region prior to imputation? The authors should present all variants tested in each of the intervals found to be genome-wide significant in this study in the supplement and indicate if each was genotyped or imputed.

5)When looking at the other autoimmune diseases, are the effect sizes similar across these loci? Are the same variants and/or haplotypes associated with AAD as the other traits?
6)The overall flow of the manuscript needs some attention. There is too much discussion of AIRE and HLA (as noted above) with little space dedicated to the 4 novel loci. The conditional work for AIRE should be with the subsection describing that gene. The heritability explained is at the end and disjointed from the summary of the GWAS at the start of the manuscript. Including a subsection for each associated region, with particular emphasis on the 4 novel loci, would greatly strengthen the impact of this work.

RESPONSES TO THE REVIEWERS COMMENTS

Reviewer #1 (Remarks to the Author):

This is a really well written and performed GWAS of Addison's Disease (AD). The study looks at just a subset of AD patients, excluding patients with APS1 (aka APACED), but leaving in patients with APS2 who make up ~2/3 of the cases. Key findings are common variant association with two variants of AIRE with AD, mutations of which cause APS1, and associations in 8 other genes, all known to be associated with a wide range of immune-mediated disorders, including particularly organ-specific autoimmune diseases. No difference in associations is seen with isolated AD and APS2.

The study is very neat and I have only a few comments/suggestions.

1. The HLA analysis is simplistic and could be improved. In some diseases imputation down to amino-acid level has proven able to identify key sequences associated with disease even on long haplotypes (e.g. (1, 2)). Including such an analysis would strengthen the study.

We have now added resolution down to amino acid level and recalculated all associations in the HLA-region accordingly. This improved the fit of the data in the model describing the HLA risk and amino acids have replaced a few of the correlated alleles as main effectors. Therefore, Figure 3 and Table 3, both describing the dissection of the HLA association have been updated with these new results. A new illustration (Figure 4) and a separate supplemental table (no. 6) describing the most important amino-acid associations have been added.

The authors have performed a logistic regression analysis of HLA alleles themselves, but it's not clear to me from the description how many different association signals the authors claim at the MHC, and what their boundaries are. Would be helpful to clarify that in the paper. In Figure 3 it would be helpful to list the conditional steps taken between each figure.

The results section have been updated to better describe the disease susceptibility that we attribute to variation in the HLA, along with the corresponding figure and legend. With the addition of amino acids, we explain the risk mediated by HLA with in total seven significant variables, which we now state explicitly. Figure 3 now shows the additional covariates added in each step of regression. The HLA section has also been linked to the supplementary note that describes the HLA associations and their dissection in greater detail (page 42 onwards).

In general, given the linkage disequilibrium between genes in the HLA, especially within class I and class II, respectively, as well as the correlation between some alleles and their unique amino acids, it is difficult, if at all possible, to pinpoint the exact causal variants by association. Hence, we have aimed at improving the transparency of the method and our discussion of its limitations. On the same note, the boundaries of the associations are

best described with Figures 3 and Supplementary Figure 3 which together visualise the extreme linkage disequilibrium and its behaviour under conditioning.

2. Whilst I think AIRE is the likely candidate gene, particularly for the coding variant rs74203920, it lies next to other strong candidate genes including ICOSLG and DNMT3L, which show association with other diseases (e.g. see (3), showing association of this locus with AS, CD, UC and PSC, but implicating DNTM3L). The functional analysis presented in Supplementary Figure 4 does not show an effect of either rs74203920 or rs1800520 on AIRE dependent gene expression, something the authors ascribe to the variants been polymorphisms. Given the substantial odds ratio that rs74203920 in particular carries, the lack of an effect in this assay suggests other explanations, such as that the cell type studied is not a relevant cell type for AIRE (HEK293 cells, renal origin, used), or that other nearby genes may be involved (though this seems intuitively less likely). These alternate options should be mentioned and discussed in the manuscript.

We agree with the comment and have added the suggested interpretations to the manuscript, and directly to the caption of the supplementary figure (no. 5) describing the results of the assay. In addition, the AIRE missense variant is prioritized in the newly added fine-mapping analysis (see below).

3. This may be for a subsequent paper, but the effect sizes reported here for AIRE and HLA are large. Diagnosis of AD is often delayed, sometimes with fatal consequences. It would be nice to see an analysis of the utility of a genetic risk score for AD.

We thank you for this excellent suggestion and agree that a comprehensive analysis of polygenic risk score requires a separate paper.

References

1. Leo PJ, Madeleine MM, Wang S, Schwartz SM, Newell F, Pettersson-Kymmer U, et al. Defining the genetic susceptibility to cervical neoplasia-A genome-wide association study. PLoS Genet. 2017;13(8):e1006866.

2. Han B, Diogo D, Eyre S, Kallberg H, Zhernakova A, Bowes J, et al. Fine mapping seronegative and seropositive rheumatoid arthritis to shared and distinct HLA alleles by adjusting for the effects of heterogeneity. Am J Hum Genet. 2014;94(4):522-32.

3. Ellinghaus D, Jostins L, Spain SL, Cortes A, Bethune J, Han B, et al. Analysis of five chronic inflammatory diseases identifies 27 new associations and highlights disease-specific patterns at shared loci. Nat Genet. 2016;48(5):510-8.

Reviewer #2 (Remarks to the Author):

This is an exceptionally interesting manuscript, describing the results of the first GWAS of Addison's disease (AAD), a rare but historically important autoimmune (AI) disease that constitutes the most common cause of primary adrenal failure. Given the rarity of AAD, this GWAS is of reasonable size to provide decent power, the work was very well performed (the scrupulous case selection is particularly impressive), the analyses seem sound (though a bit limited), the interpretations are generally reasonable, and the writing is excellent. The man findings are of genetic association of AAD with a number of genes that largely have been previously found associated with other AI diseases, many of which are epidemiologically associated with AAD. The only truly novel finding is of polygenic association with two coding variants in AIRE, mutations in which are causal for autosomal recessive APECED, a multiple AI disease that primarily includes AAD, and which have not previously been associated with risk of other autoimmune diseases. The findings regarding AIRE constitute the main novel result, seem of considerable interest, but deserve much deeper analyses than are reported here.

Specific suggestions:

1. It seems to me the title is overstated. Given the findings of GWAS of other AI diseases, I think it can safely be said that many (if not most or all) AI diseases, including AAD, involve components of loss of both peripheral and central tolerance. The only real difference is the involvement of AIRE in AAD, which suggests a perhaps greater role for loss of central tolerance in AAD than in other AI diseases. Nevertheless, I do believe the authors should change the title to one more descriptive of their findings.

We have now changed the title to a more descriptive one:

GWAS for autoimmune Addison's disease identifies multiple risk loci and highlights AIRE in disease susceptibility.

2. Regarding the reported associations with coding variants in AIRE, the authors describe rs74203920-T as "relatively rare in our Swedish and Norwegian controls (2.0%) and in thenon-Finnish European population (1.4% GnomAD v2.1.1)". However, that MAF is, by definition, not "rare". Rather than saying "relatively rare" I would suggest "uncommon".

We have changed the wording to "uncommon".

Unless I missed it somewhere, given that APECED is recessive, there should be a formal analysis of association of AAD with the two AIRE variants singly and together under both polygenic and recessive models, and a corresponding table of the results presented at least in the supplementary material.

The suggested analysis has been added with a corresponding table in the supplementary material (ST4). The associations with *AIRE* were best described by polygenic, additive models. As the second AIRE variant (rs2075876) has a very high risk allele frequency 95/90% in cases/controls, carriers of the risk allele for the first AIRE variant (rs74203920) almost always carry at least one risk allele for the second. Therefore, a recessive model of inheritance for AAD

related to compound heterozygotes for these variants is inconsistent with the prevalence of the disease.

Furthermore, given that AIRE is on chr21, a sentence or two of speculation in the Discussion on the possible role of AIRE in the very high prevalence of AI diseases in patients with Down Syndrome would not be unwarranted.

We now discuss the role of *AIRE* in Down Syndrome and include literature references (page 14).

3. The section, “Sub-significant loci….” reports nothing of interest and should be deleted in its entirety. That will make room for other analyses that would be of greater interest.

This section has now been deleted.

4. Similarly, the section, “Gene set enrichment analysis…” also reports nothing novel, which is not surprising as all of the loci that are discussed are well known in other AI diseases. This section should be reduced to literally a single sentence or two in the discussion.

This section has been substantially reduced and moved accordingly.

5. Nevertheless, Fig. 4 and Supplementary Fig. 5 are of considerable interest; I believe that analysis should be expanded in the present report.

We have expanded this section and moved Supplementary Figure 5 (*Circos plot*) from the supplementary material to the manuscript, now labelled Figure 5C. We have also added side-by-side comparisons of association statistics across autoimmune diseases, for all AAD risk loci (Suppl. Fig. 7), and a smaller panel in the manuscript as Figure 5D.

It has long been noted that occurrence of other AI diseases is much more common in AAD cases than in other AI diseases; is evident even in Addison’s original case study. Accordingly, a Supplementary Table reporting the nature and prevalence of other AI diseases in the cases used in this GWAS is absolutely required here.

The suggested table is present as Supplementary Table 1.

Second, it has been speculated that the rarity of AAD coupled with the exceptionally high epidemiological association with other AI diseases suggests that these diseases are all genetically related, but that might be biologically “harder” to get AAD than these other diseases (in terms of a burden threshold). As twin heritability is close to 1, that suggests the effect is almost completely genetically determined. Might the authors be able to carry out an analysis of the genetic burden conferred by the non-AIRE AAD-associated alleles in AAD versus the other AI diseases to which these loci contribute? Alternatively, might it be that

the AIRE variants associated here are essentially necessary but not sufficient for AAD? Could the authors partition their AAD cases into subsets with versus without the associated AIRE variants and test for differential association with the other loci they report? These types of questions would seen to warrant analysis and discussion, but are generally absent from the manuscript.

As suggested, we have partitioned the AAD cases into subsets "With" and "Without" the associated *AIRE* variants, respectively. To explore properties of *AIRE* risk allele carriers, we tested for association in (1) With versus Without, (2) With vs. Healthy controls, and (3) Without vs. Healthy controls, and have added the results to the supplementary material (Supplemental Tables 4 and 5). In short, we find no differences between the groups when it comes to frequencies of other risk alleles, or other autoimmune comorbidities. Taken together, the associated AIRE variants exert their risk effect independently from each other and from other risk loci.

Given a minor allele frequency of 6.5% in cases, only about 12.7% of cases have p.R471C, therefore it can not be considered necessary for AAD development, nor sufficient. The corresponding allele frequency for p.S278R is 90% in controls and hence not sufficient to explain why some individuals at risk of autoimmunity acquire AAD.

We are eager to compare the burden of risk alleles in AAD and other autoimmune diseases in a comprehensive way. It would, however, require individual level genotype data for all subjects in the comparative studies, which is not available to us at the present time. We instead investigate the overlap of associated risk loci, and the effect sizes linked to autoimmune diseases (please see response to point 5 from reviewer 3 below).

We also refer to initiative to estimate co-heritability across autoimmune diseases that circumvents the need for genotype data from individuals. Although this approach has other limitations, a recent epidemiological effort estimated co-heritability of organ-specific autoimmune diseases:

Skov, J., Eriksson, D., Kuja-Halkola, R., Höijer, J., Gudbjörnsdottir, S., Svensson, A., Magnusson, P., Ludvigsson, J., Kämpe, O., & Bensing, S. (2020). Co-aggregation and heritability of organ-specific autoimmunity: a population-based twin study, European Journal of Endocrinology, 182(5), 473-480.

6. Along the same lines, the authors report a GCTA analysis using the GWAS data, but it is difficult to understand what they report. They state that previous studies report twin heritability for AAD of 97%. From the GCTA analysis and AAD prevalence studies, they report "a SNP heritability rate for AAD between 34% and 40%". I am not sure what they mean by "SNP heritability rate". Do they mean h2? Obviously, the key questions are what fraction of twin heritability is captured by h2 as measured by GCTA, which here included only common SNPs with MAF >/ 1% (the meaning of the last sentence of the abstract is especially unclear in this regard, and should be rewritten); what fraction of that is accounted for by the associated loci they report based on the GWAS results; and what fraction of that is accounted for by the two AIRE variants, which are novel to this study.

Heritability explained by all SNPs covered by the GWAS: We have tried to clarify all descriptions of heritability explained in the abstract and manuscript. In brief, all SNPs covered by the GWAS explain 35-41% of the h2 estimated from twin studies (SNP heritability divided by h2. Hence, lower boundary 34%/97% = 35%; and upper boundary 40%/97% = 41%).

Heritability explained by SNPs associated to AAD in GWAS:
Since the associated SNPs are ascertained by p-value from GWAS analysis in the same sample, the GCTA estimate of variance explained by this subset of SNPs will inevitably be inflated due to the winners' curse issue, i.e. the selection creates a positive correlation between true SNP effects and estimation errors. Hence, a good estimate of variance explained by associated SNPs will require an independent sample. We have refrained from performing this analysis for the manuscript as of now, but wish to come back to this question in future work on polygenic risk estimates in AAD.

The last sentence in the abstract has also been amended.

Reviewer #3 (Remarks to the Author):

The authors of this manuscript describe the first GWAS of AAD. While several studies have explored candidate loci usually that had been associated with other autoimmune diseases prior to this work, only a few loci had surpassed genome-wide significance. While they were able to replicate these loci, they also found 4 novel associations. The authors go on to explore the signal near AIRE identifying a second independent signal for what had been previously reported. The HLA is the strongest association with AAD, and after classical allele imputation and stepwise regression, HLA DQB1*02:01 and DQB1*03:02 were the top signals in the region. The authors then show how the associated loci relate to other autoimmune diseases. This work is a very important step towards understanding the genetic determinants of AAD; however, further evaluation of the loci should be done in the loci outside of those previously implicated prior to this study. While the work in the HLA and AIRE are
important, very little was done in the other regions to further our understanding of how they might contribute to AAD pathophysiology, particularly the 4 novel loci. In addition, here are some specific points:

1) The authors should use Bayesian statistics to identify credible sets of variants for all loci identified in this study. Currently, the authors have only discussed conditional analysis on AIRE and stepwise with the HLA.

In response to this and point 2 below, we have added a Bayesian fine-mapping analysis, based on a shotgun stochastic search of 1Mb windows around the lead GWAS SNP, to identify credible sets and configurations, and evaluated the variants included for functional categories. Results are given in Supplementary Table 2, and discussed in the manuscript (Results and Discussion, see point 6 below).

2) The authors should use this information from the creditable sets to evaluate each region for eQTLs, chromatin-chromatin iterations, evidence of altering protein binding motifs and/or ChIP-seq peaks, histone marks, etc. from bioinformatics dataset that widely available for immune cell populations. This can then help shorten the credible set further.

See above.

3) The plot of the PCA should be shown in the supplement. What was the rationale for using 5 PCs as covariates in the regression model?

Several plots of the PCA dimensions have now been added to the supplement (Supplementary Figure 1). PCs 1-5 together explained 42% of the variance, with subsequent PCs explaining less than 4.5% each. Including PCs 1-5 as covariates should then adequately account for any residual population structure in the data, which had already been limited by the careful sampling strategy and exclusion non-Europeans based on the PCA.

4) The authors do not indicate which variants were imputed and which were genotyped. Was there association signals in each genome-wide significant region prior to imputation? The authors should present all variants tested in each of the intervals found to be genome-wide significant in this study in the supplement and indicate if each was genotyped or imputed.

We have added the requested plots in the supplement alongside the LocusZoom plots in Supplementary Figure 3. In all associated loci, the peaks contain associated genotyped markers. However, on chromosomes 12 and 19, the imputed variants are barely genome-wide significant, and the most significant genotyped markers have P = 7.8e-8 and P = 4.3e-7, respectively. For clarification, we have also added this information in the manuscript sections describing these loci.

5) When looking at the other autoimmune diseases, are the effect sizes similar across these loci? Are the same variants and/or haplotypes associated with AAD as the other traits?

The manuscript text section describing overlap with other autoimmune diseases, and the Figure 5 have been expanded to address these questions. Detailed plots for each locus and for each disease have been added to the supplementary information to enable direct comparison of association signals, together with an overview in Figure 4. Using PhenoScanner and GWAS catalog, we have also added the effect sizes for shared associations. Taken together, the variants, risk alleles, and their effects are strikingly often shared between diseases, in line with the high prevalence of autoimmune comorbidity in AAD (Supplementary Figures 7 and 8).

6) The overall flow of the manuscript needs some attention. There is too much discussion of AIRE and HLA (as noted above) with little space dedicated to the 4 novel loci. The conditional work for AIRE should be with the subsection describing that gene. The heritability explained is at the end and disjointed from the summary of the GWAS at the start of the manuscript. Including a subsection for each

associated region, with particular emphasis on the 4 novel loci, would greatly strengthen the impact of this work.

To complete and to balance the manuscript, sections describing the four novel loci have been added to the manuscript, along with additional supplementary plots aiding the interpretation of these results (Suppl. Fig 8). The conditional work on *AIRE* has been moved as suggested.

Reviewer #1 (Remarks to the Author):

I thank the authors for their constructive response to my suggestions. I look forward to seeing the PRS paper in due course. I have no further comments to add.

Reviewer #2 (Remarks to the Author):

The authors have been highly responsive to the previous critiques. I have no further concerns

Reviewer #3 (Remarks to the Author):

I have no further comments.
